# Supplementary figures and images for: COVID19 Sero-epidemiology and vaccine uptake in two Southern Nigerian clinics
Source: PLOS Glob Public Health. 2026 Mar 27;6(3):e0006126. doi: 10.1371/journal.pgph.0006126 (PMC13029786; doi:10.1371/journal.pgph.0006126)

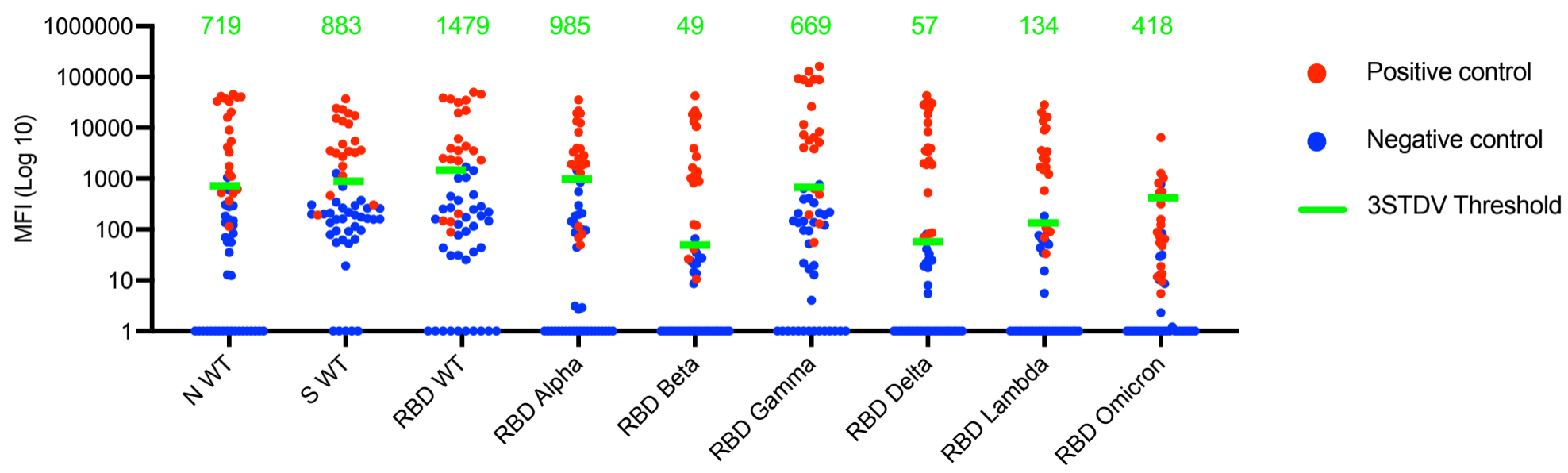

Supplement: S1 Fig — The graph shows raw median fluorescence intensity (MFI) outcomes of the positive controls (red dots) and the negative controls (blue dots) by SARS-CoV-2 variant. In green are the 3STDV thresholds (based on negative controls only), with respective values on top. Note, negative MFI values (artifact of the assay and not meaningful outcomes since there cannot be a negative value of antibodies in the samples) were set to 1 to plot the log(10) scale graph and better visualize the full spread of MFIs. MFI, median fluorescence intensity. S, Spike. RBD, receptor binding domain. N, Nucleoprotein. (PDF) [file pgph.0006126.s001.pdf]

A

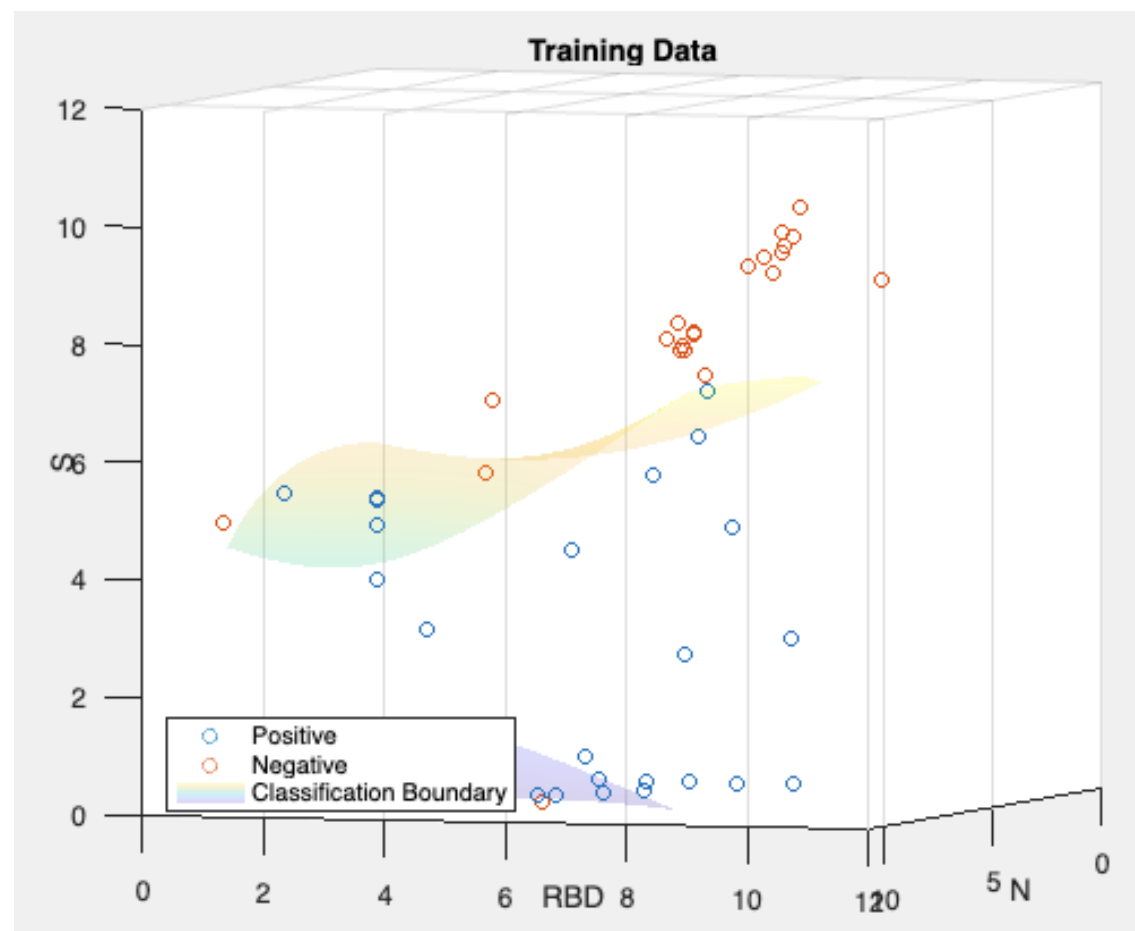

B

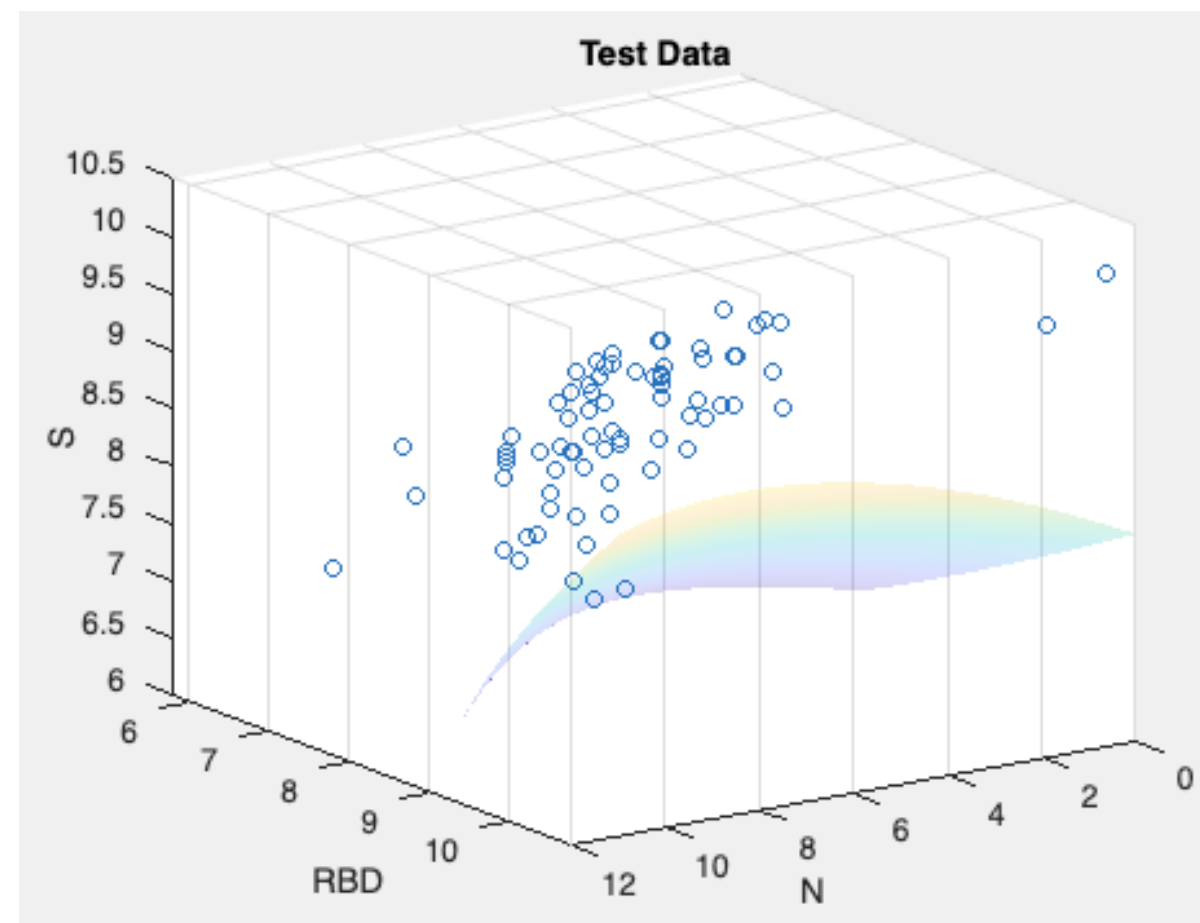

Supplement: S2 Fig — (A) This figure shows the classification boundary (i.e., three-dimensional threshold in rainbow colours) created by the SeroNIST fool for wildtype (WT) RBD, S, and N antibodies based on the training data (i.e., negative control in blue dots and positive controls in red dots). (B) This figure shows where the data points (blue dots) fall for an example data set of participants. In this case, all the data points fall above the classification boundary, resulting in an estimated SARS-CoV-2 seroprevalence of ~100% based on RBD, S, and N antibodies. (PDF) [file pgph.0006126.s002.pdf]
