# Supplementary material for: COVID19 Sero-epidemiology and vaccine uptake in two Southern Nigerian clinics
Source: PLOS Glob Public Health. 2026 Mar 27;6(3):e0006126. doi: 10.1371/journal.pgph.0006126 (PMC13029786; doi:10.1371/journal.pgph.0006126)
Supplement: Attachment — Submitted filename: PGPH-D-25-02354_Rev.pdf [file pgph.0006126.s020.pdf]

PLOS Global Public Health

COVID19 Sero-epidemiology and Vaccine Uptake in Two Southern Nigerian Clinics

--Manuscript Draft--

**Manuscript Number:**	PGPH-D-25-02354
**Article Type:**	Research Article
**Full Title:**	COVID19 Sero-epidemiology and Vaccine Uptake in Two Southern Nigerian Clinics
**Short Title:**	COVID-19 and Vaccine Uptake in Southern Nigeria
**Corresponding Author:**	Raquel A. Binder, PhD, MPH<br>University of Massachusetts Chan Medical School<br>Worcester, MA UNITED STATES OF AMERICA
**Order of Authors:**	Chinedu A. Ugwu
	Ooreofe Odebode
	Israel O. Ajayi
	Josiah Olubunmi
	Bejide Ifeoluwa
	Jolly A. Adole
	Kazeem Akano
	Johnson Okolie
	Philomena Eromon
	Imonikhe K. Kio
	Judith Amadi
	Precious F. Adebayo
	Iheanyi Mgbeokwere
	Akeem O. Lawal
	Olayemisi A. Adegoke
	Amoke O. Adeyemi
	Sampson Owhin
	Oladele O. Ayodeji
	Alebiosu Ahmed
	Etim Henshaw
	Oyejide E. Nicholas
	Abah A. Sylvester
	Abiola Omidele
	Kolade Emilola
	Olayiwola Funmilayo
	Adeboye Rufus Oyewale
	Oluwatuyi O. Olakunle
	Obaado Oluwaseun
	Angela M. Matta
	Prajakta Bedekar

Powered by Editorial Manager® and ProduXion Manager® from Aries Systems Corporation

	Latifeh Dahmash
	Catherine S. Forconi
	Ann M. Moormann
	Rhoel R. Dinglasan
	Christian T. Happi
	Raquel A. Binder, PhD, MPH
**Keywords:**	COVID-19, Nigeria, Serosurvey, Vaccine Uptake, Threshold method
**Abstract:**	Background: Africa has reported lower COVID-19 morbidity and mortality compared to other regions. Yet, the flat COVID-19 trajectory remains to be fully explained. Nigeria, Africa's most populous country, accounted for less than 1% of global COVID-19 cases. The present study evaluated SARS-CoV-2 seroprevalence, vaccine uptake, and methodological differences in serological threshold setting among febrile patients attending peri-urban and urban clinics in Southern Nigeria. Methods: Between October 2022 and February 2023, 745 febrile participants were recruited from Owo (Ondo State) and Ikorodu (Lagos State). Serum samples were tested for IgG antibodies against wild-type and variant SARS-CoV-2 antigens using a multiplex bead-based antibody assay. Seroprevalence was calculated using two classification boundary methods: (i) the conventional three standard deviations above the mean of negative controls (3STDV) and (ii) the open-source SeroNIST tool. Vaccine uptake and risk factors for exposure were assessed. Results: Vaccine uptake was 66.6% and significantly higher in peri-urban Owo (80.2%) than urban Ikorodu (52.6%, $p < 0.001$). Increasing age was positively associated with vaccination ($p < 0.001$). Among unvaccinated participants, the anti-RBD/S SARS-CoV-2 seroprevalence was remarkably high and ranged from 89.1% to 100%. No significant differences in seroprevalence were observed across sex, education, or religion. Anti-N seroprevalence was similar among the vaccinated and unvaccinated groups, suggesting that both groups were exposed equally to SARS-CoV-2 prior to our study period. Conclusions: SARS-CoV-2 exposure in Southern Nigeria was likely widespread and underrecognized, with high seroprevalence among symptomatic peri-urban and urban populations. Peri-urban communities demonstrated higher vaccine uptake than urban counterparts, and age-targeted campaigns likely drove uptake among older individuals. Standardized thresholding methods such as SeroNIST are critical for accurate seroprevalence estimates.
**Additional Information:**	
**Question**	**Response**
**Financial Disclosure**<br><br>Enter a financial disclosure statement that describes the sources of funding for the work included in this submission and the role the funder(s) played. This includes grants and any commercial funding of the work or authors.<br><br>This statement will be typeset if the manuscript is accepted for publication.<br><br>*Please review the submission guidelines and the instructions link below for detailed requirements and guidance.*	Funding was provided by (1) the Centers for Disease Control and Prevention (CDC) U01GH002338-01 grant titled "Seroepidemiological Insight into COVID-19 Transmission in Africa (SICA)", (2) the National Center for Advancing Translational Sciences KL2-TR001455 grant, and (3) the Bill and Melinda Gates Foundation GIISER Project (ARISE-INV-036304). The funders had no role in study design, data collection and analysis, decision to publish, or preparation of the manuscript.

Powered by Editorial Manager® and ProduXion Manager® from Aries Systems Corporation

**Competing Interests**<br><br>On behalf of all authors, disclose any competing interests that could be perceived to bias this work.<br><br>This statement will be typeset if the manuscript is accepted for publication.<br><br>*Please review the instructions link below and PLOS Global Public Health's competing interests policy to determine what information must be disclosed at submission.*	The authors have declared that no competing interests exist.
**Data Availability**<br><br>Before publication, Authors are required to make fully available and without restriction all data underlying their findings. Please see our PLOS Data Policy page for detailed information on this policy.<br><br>A **Data Availability Statement**, detailing where the data can be accessed, is required at first submission. Insert your Data Availability Statement in the box below.<br><br>Please see the data reporting section of our submission guidelines for instructions on what you need to include in your Data Availability Statement.<br><br>This statement will be typeset if the manuscript is accepted for publication.<br><br>*PLOS allows rare exemptions to address legal and ethical concerns. If you have legal or ethical restrictions, please use the box below to detail these in full sentences for the Journal team to consider.*	The full database has been submitted along with the manuscript and figures.

Manuscript

Click here to access/download;Manuscript;SICA ACEGID Manuscript_8 12 25.docx

COVID19 Sero-epidemiology and Vaccine Uptake in Two Southern Nigerian Clinics

Chinedu A. Ugwu[1,2], Ooreofe Odebode[1,2], Israel O. Ajayi[1,2], Josiah Olubunmim[1,2], Bejide Ifeoluwa[1,2], Jolly A. Adole[1,2], Kazeem Akano[1,2], Johnson Okolie[1], Philomena Eromon[1], Imonikhe K. Kio[1], Judith Amadi[1], Precious F. Adebayo[1], Iheanyi Mgbeokwere[1], Akeem O. Lawal[3], Olayemisi A. Adegoke[3], Amoke O. Adeyemi[1], Sampson Owhin[4], Oladele. O. Ayodeji[4], Alebiosu Ahmed[1], Etim Henshaw[1], Oyejide E. Nicholas[1], Abah A. Sylvester[1,4], Abiola Omidele[1,4], Kolade Emilola[1], Olàyiwolà Funmilayo[1], Adeboye Rufus Oyewale[1], Oluwatuyi O. Olakunle[1], Obaado Oluwaseun[1], Angela M. Matta[5], Prajakta Bedekar[6,7], Latifeh Dahmash[8], Catherine S. Forconi[5], Ann M. Moormann[5], Rhoel R. Dinglasan[8], Christian T. Happi[1,2], Raquel A. Binder[5*]

Affiliations

[1]The Africa Centre of Excellence for Genomics of Infectious Diseases (ACEGID), Redeemer's University Nigeria, Ede, Nigeria.

[2]Department of Biological Sciences, Faculty of Natural Sciences, Redeemer's University, Ede, Osun State Nigeria.

[3]Ikorodu General Hospital Ikorodu, Lagos State Nigeria

[4]Federal Medical Centre Owo, Ondo State Nigeria

[5]Department of Medicine, University of Massachusetts Chan Medical School, Worcester, MA, USA

[6]Department of Applied Mathematics and Statistics, Johns Hopkins University, Baltimore, MD, USA

[7]Department of Mathematics, BITS Pilani, Hyderabad, Telangana, India

[8]Department of Infectious Diseases & Immunology, College of Veterinary Medicine, University of Florida, Gainesville, FL, USA

***Corresponding author**: raquel.binder@umassmed.edu

Running title: COVID-19 and Vaccine Uptake in Southern Nigeria

Abstract word count: 269/300

Main text word count: 4,742

Abstract

Background: Africa has reported lower COVID-19 morbidity and mortality compared to other regions. Yet, the flat COVID-19 trajectory remains to be fully explained. Nigeria, Africa's most populous country, accounted for less than 1% of global COVID-19 cases. The present study evaluated SARS-CoV-2 seroprevalence, vaccine uptake, and methodological differences in serological threshold setting among febrile patients attending peri-urban and urban clinics in Southern Nigeria. Methods: Between October 2022 and February 2023, 745 febrile participants were recruited from Owo (Ondo State) and Ikorodu (Lagos State). Serum samples were tested for IgG antibodies against wild-type and variant SARS-CoV-2 antigens using a multiplex bead-based antibody assay. Seroprevalence was calculated using two classification boundary methods: (i) the conventional three standard deviations above the mean of negative controls (3STDV) and (ii) the open-source SeroNIST tool. Vaccine uptake and risk factors for exposure were assessed. Results: Vaccine uptake was 66.6% and significantly higher in peri-urban Owo (80.2%) than urban Ikorodu (52.6%, $p < 0.001$). Increasing age was positively associated with vaccination ($p < 0.001$). Among unvaccinated participants, the anti-RBD/S SARS-CoV-2 seroprevalence was remarkably high and ranged from 89.1% to 100%. No significant differences in seroprevalence were observed across sex, education, or religion. Anti-N seroprevalence was similar among the vaccinated and unvaccinated groups, suggesting that both groups were exposed equally to SARS-CoV-2 prior to our study period. Conclusions: SARS-CoV-2 exposure in Southern Nigeria was likely widespread and underrecognized, with high seroprevalence among symptomatic peri-urban and urban populations. Peri-urban communities demonstrated higher vaccine uptake than urban counterparts, and age-targeted campaigns likely drove uptake among older individuals.

Standardized thresholding methods such as SeroNIST are critical for accurate seroprevalence estimates.

Key words: COVID-19, Nigeria, Serosurvey, Vaccine Uptake, Threshold method

Introduction

Severe acute respiratory syndrome coronavirus 2 (SARS-CoV-2), causing coronavirus disease 2019 (COVID-19), was first identified in Wuhan, China, in December of 2019 and has since rapidly disseminated across the globe, causing the biggest pandemic of the 21st century (1). Close to 800 million cases and around 7 million deaths have been attributed to COVID-19, which is likely underestimating the true global burden (2). Surprisingly, Africa experienced a less catastrophic outbreak compared to the rest of the world, accounting for less than 2% of the global cases (3). Nigeria, the most populated country in Africa, accounted for just 0.003% of the global cases (2). At first, limited testing, a younger population, reduced transmission, preexisting immunity, genetic protective factors, early implementation of public health measures, and timely leadership were proposed to drive the flat COVID-19 trajectory (4,5). More recent evidence suggests that reduced viral transmission and poor surveillance were not the main reasons the reported COVID-19 morbidity and mortality were low (6). Instead, the population age structure and a reduced anti-SARS-CoV-2 inflammatory response due to specific immunoregulation or cross-reactive pre-pandemic immunity are likely the main protective factors (6). Similar factors were likely the reason a low number of COVID-19 cases and related deaths were reported in Nigeria (7,8). To better understand the extent of existing immunity to SARS-CoV-2 in Nigeria and risk factors of infection, we implemented a study measuring seroprevalence against different variants of SARS-CoV-2 between October 2022 and February 2023 in two Southern Nigerian clinics covering peri-urban and urban settings. Given the varying vaccine availability and acceptability in the region, we also evaluated the COVID-19 vaccine uptake. At the time of the study, seven vaccines were approved for emergency use in Nigeria: Vaxveria (Oxford/ AstraZeneca), Covishield (Serum Institute of

India), Comirnaty (Pfizer/BioNTech), Jcovden (Johnson and Johnson), Spikevax (Moderna), Sputnik V (Gamaleya), and Covilo (Sinopharm). By 2023, ~110 million doses of COVID-19 vaccines had been distributed and ~30% of the population had full vaccine coverage (9,10).

Sero-epidemiological studies (serosurveys) evaluate pathogen-specific antibodies, thus serving as a tool to evaluate the burden of infection and existing immunity among populations. Serosurveys are particularly valuable in contexts where clinical reporting and molecular testing for infectious diseases are constrained (11). There are limited SARS-CoV-2 serosurveys implemented in Nigeria, and most of them focused on health care workers or only assessed the ancestral SARS-CoV-2 strain (i.e., wildtype [WT] or Wuhan) (11–16). Between February 2020 and November 2022, Nigeria had experienced four major COVID-19 waves, which were dominated by the wildtype (WT)/ancestral, Alpha, Delta, Eta, and Omicron variants (17–20). Other important variants detected elsewhere, such as Gamma and Lambda, were not reported in Nigeria. Whether this means these variants did not circulate in Nigeria or whether they went unreported is unclear.

While serosurveys are essential public health tools, the applied downstream analysis methods to convert quantitative outcomes (e.g., median fluorescence Intensity [MFI] values from bead-based assays or optical density [OD] values from enzyme-linked immunosorbent assays [ELISAs]) into qualitative results (positive/negative) vary greatly, making metanalyses and comparisons of outcomes across studies and regions challenging (21,22). Here, we applied a validated weighted-standard curve across-plate normalization method (23) and compared two classification boundary methods for optimal qualitative serological assessments. (1) The traditionally applied three standard deviations above the mean of the negative controls (3STDV), and (2) an open-source machine learning-based tool that calculates sensitive and specific classification boundaries based on population-specific positive and negative controls, which has been termed SeroNIST (22–25).

In summary, we implemented a 2022/2023 SARS-CoV-2 serosurvey in Southern Nigeria based on standardized serological tools, determined the risk factors for infection, and evaluated the COVID-19 vaccine uptake among symptomatic individuals visiting local health

care centers. Understanding the sero-epidemiological dynamics of the disease and contrasting peri-urban and urban settings is crucial for assessing the population's exposure, immunity levels, and potential risk factors. By examining the SARS-CoV-2 seroprevalence in Southern Nigeria, this study bridged knowledge gaps about regional risk factors for infection and population-level exposures. Finally, assessing COVID-19 vaccine uptake allowed us to evaluate the potential impact of vaccine-associated reduced disease that can inform future public health strategies aiming to increase vaccine coverage.

Methods

Study Clinics

The study was conducted at two clinical sites in Southern Nigeria: (1) The Federal Medical Centre, Owo (FMC Owo), in Ondo State from October 19 to November 22, 2022, and (2) the General Hospital, Ikorodu (Ikorodu GH) in Lagos State from November 14, 2022, to February 15, 2023. These sites were selected based on their capacity to support surveillance research among febrile populations and their existing clinical diagnostic and research infrastructure. Both facilities serve large catchment populations and represent diverse epidemiological settings, covering both peri-urban (Owo) and urban (Ikorodu) sites and therefore offering a unique opportunity to assess SARS-CoV-2 exposure and COVID-19 vaccine uptake.

FMC Owo is a federal tertiary healthcare institution with peri-urban extension services and a fully functional laboratory run by the Africa Centre of Excellence for Genomics of Infectious Diseases (ACEGID). The facility supports a wide range of diagnostic capabilities and biological sample processing. Ikorodu GH is a high-volume secondary health facility serving an urban population just outside metropolitan Lagos, with clinical services and infrastructure well-suited to the project’s needs.

Both sites provided centralized coordination of participant enrolment, data collection, and sample processing. Field teams were trained in Good Clinical Practice (GCP), biosafety procedures, and the use of standardized data collection tools. Community engagements were carried out at both sites to ensure culturally appropriate and voluntary participation. The study

design ensured contextual relevance, operational feasibility, and scientific rigor for characterizing the SARS-CoV-2 epidemiology and COVID-19 vaccine uptake in distinct healthcare settings within southern Nigeria.

Human Subjects

The study protocol, informed consent procedures, and all associated study tools were reviewed and approved by the Nigerian National Health Research Ethics Committee (NHREC) and the Health Research Ethics Committees (HRECs) of Owo FMC (NHREC/01/01/2007-31/08/2021). Similarly, the University of Florida Institutional Review Board (IRB) reviewed and approved the study and associated documents (IRB202102308). Ethical oversight covered all aspects of study implementation, including participant recruitment, data collection, and biological sampling.

Inclusion criteria: febrile individuals across all ages seeking care at either study clinic. All participants provided written informed consent prior to enrolment. For participants under the age of 18, assent was obtained in conjunction with parental or guardian consent. Trained field personnel conducted the consent process using IRB-approved forms. The consent process clearly outlined the study objectives, procedures, potential risks, and the voluntary nature of participation. Participants were informed of their right to withdraw from the study at any time without penalty. Confidentiality was maintained throughout the study. Each participant was assigned a unique identifier, which was used to label all data and specimens. Electronic data were entered into encrypted REDCap databases hosted on password-protected devices. To mitigate risks, field staff were trained in infection prevention and control practices and used personal protective equipment during all participant interactions. These measures ensured ethical standards were upheld while maximizing participant safety and study integrity.

Sample and Data Collection

Participant recruitment occurred through the outpatient departments of both hospitals. Individuals presenting with acute febrile illness were screened for eligibility (i.e., consenting abilities) and invited to participate. Post-consent, a brief survey (capturing basic demographics and COVID-19 vaccine uptake) and a blood sample were collected. Each participant provided

finger-prick whole blood through an EDTA microtainer tube. Serum was separated onsite using portable centrifuges and stored under cold conditions. Each sample was labelled with a unique study identifier and linked to participant data through encrypted REDCap databases. Processed samples were transported to the ACEGID laboratory at Redeemer's University for bead-based multiplex serology (Luminex MagPix).

Serology - Multiplex Bead-based Antibody Assay

A bead-based IgG SARS-CoV-2 multiplex assay was implemented as previously described (23). Briefly, wild-type (WT; Wuhan) full-length spike (S), WT nucleocapsid (N), WT receptor-binding domain (RBD), RBD Alpha, RBD Beta, RBD Gamma, RBD Delta, RBD Lambda, and RBD Omicron SARS-CoV-2 antigens were coupled to MagPlex microspheres (Luminex Corporation). Bovine Serum Albumin (BSA) coupled beads were used to control for bead-specific background noise. All coupled beads were diluted in ABE buffer (PBS, BSA 0.1%, TWEEN 20%, Sodium Azide 0.05%) to make a master mix of all the coupled antigens (consistent concentration across all beads, 1,000,000 beads/mL). Then, the master mix was added to each well of a 96-well plate and placed on a magnet to allow the beads to settle and remove the ABE buffer. Subsequently, 50 µL of human serum samples diluted (1:100) in ABE buffer were added to the wells, followed by a 2-hour incubation period on a plate shaker (300 rpm). Next, the beads were washed three times with 125 µL ABE buffer while plate remained on the magnet. Then 50 µL of biotinylated anti-human secondary IgG (BD Pharmingen) diluted in ABE (1:1000) was added to each well and followed by a one-hour incubation at room temperature on a plate shaker (300 rpm). The beads were once again washed three times, and 50 µL of phycoerythrin-conjugated streptavidin (1:1000; BD Pharmingen catalog # 554061) diluted in ABE added to the wells. Next, the beads were set on the plate shaker at 300 rpm for 15 minutes. After the final incubation period, the beads were washed 3 times for the last time and resuspended in 125 µL of ABE. After resuspension, the plate was read on the Luminex MagPix instrument, where a median fluorescence intensity (MFI) value was generated for each sample and individual antigen with the corresponding bead count. Once the control MFIs (positive and negative controls), standards (dilution of positive control), and

bead counts (minimum 50 beads per antigen in each well) were validated, BSA was subtracted (including for the standards) to account for non-specific binding. Next, we applied an across-plate normalization factor based on the standards and transformed the MFIs into qualitative (positive and negative outcomes) as previously described (22–25). Previously collected de-identified banked blood/serum samples served as negative (n=36) and positive (n=21) controls (8).

Statistics

Statistical calculations and graphs were completed in R Studio (version 2024.09.0+375), GraphPad Prism (version 10.5.0), and the open-source SeroNIST tool (available at following link: https://github.com/SeroNISTPI/SeroNIST).

Frequency and percentages were used to describe the sociodemographic descriptors and vaccine uptake among the study participants (Table 1). The Pearson's Chi-squared test with Yates' continuity correction was used to assess differences in vaccine up-take based on sociodemographic variables and by region, while excluding missing data (assuming it was missing at random). To assess the association between age (as continuous variable) and vaccine uptake a logistic regression was applied, while excluding missing data (same, assuming it was missing at random). The resulting estimate for age was used to calculate Odds Ratios.

To visualize anti-SARS-CoV-2 WT and variant antibodies among vaccinated and unvaccinated study participants, raw median fluorescence intensity (MFI) and respective geometric mean and 95% CI were plotted (Figure 1 and Figure 2). The geometric mean (vs. standard average) was applied to minimize the effect of outliers. Differences between vaccinated and unvaccinated groups for each SARS-CoV-2 WT and variants were assessed with the unpaired nonparametric Mann–Whitney U test (a.k.a. Wilcoxon rank-sum test; Figure 1). Note, while the graph in Figure 1 is in Log(10) scale to visualize the MFI spread, the test was applied to raw/non-transformed numbers.

We utilized two methods to calculate the seroprevalence of SARS-CoV-2 WT and variants: (1) The first approach was a traditionally applied method where the mean and

standard deviation (STDV) of the negative controls is calculated and then the STDV multiplied by 3 and added to the mean (mean+3*STDV=3STDV threshold) and set as cut off. This method assumes the reads of the negative controls are normally distributed. Since the MFIs of our negative controls were skewed (i.e., not normally distributed data) we attempted to log transformed as previously described (22). However, log(10) transforming the MFI values of the negative controls for each antigen/isotype skewed the distribution even more, so the raw values were used to calculate the 3SDTV, likely underlining the inadequacy of this method for other data sets since negative controls will tend to accrue around zero or the lower limit of detection of the applied assay. Note, negative MFI values (artifact of the assay and not meaningful outcomes since there cannot be a negative value of antibodies in the samples) were set to 1. Similarly, values of zero (n=6) were set to 1 to be able to show the spread of the data in log(10). See Supplemental Figure 1 for the raw MFI of the negative and positive controls, along with the calculated 3STDV thresholds. (2) The second approach is a standardized open-source machine learning-based method that calculates 1-, 2-, and 3-dimensional classification boundaries based on confirmed positive and negative controls, as previously described (i.e., SeroNIST) (20–23). Each dimension corresponds to the number of antigens included, (i.e., equivalent to 1, 2, or 3 antigens). For example, 3 antigens covered WT RBD, N, and S to estimate SARS-CoV-2 seroprevalence (Table 4 and Supplemental Figure 2). The resulting classification boundary is used as threshold, same as for the 3STDV method. For the SeroNIST method, raw MFI values were used (no adjustment for negative values or zeros necessary).

Results

Study Population and Demographics

A total of 745 participants were recruited from Owo in Ondo State (n=375) from October to November 2022 and Ikorodu in Lagos State (n=370) from November 2022 to February 2023. About half of the participants were female (n=430, 57.7%), while 28.9% (n=215) were male, and 13.4% (n=100) did not report sex. Ages ranged from 0 to 84 years (average: 35.1 years,

STDV: 14.7 years), while the majority of participants were between 18 and 45 years of age (n=497, 66.7%). The majority of participants had a secondary education (n=389, 52.2%), followed by those with university education (n=164, 22.0%), and then those with primary school education (n=130, 17.4%). Christianity was the dominant self-reported religion among our participants (n=570, 76.5%), followed by Islam (n=138, 18.5%), and those who practised other religions (n=36, 4.8%). Only one person reported not practicing any religion (0.1%).

Vaccine Uptake

Among those who reported vaccine status (n=739), the majority (n=492, 66.6%) reported having received either a single or double dose of the COVID-19 vaccine (n=247, 33.4% not vaccinated). Note, six participants were missing vaccine response. The majority of the participants from Owo (peri-urban, Ondo State) were vaccinated (n=300, 80.2%, Table 1). The vaccine uptake in Ikorodu (urban, Lagos State) was also relatively high (n=192, 52.6%) but lower than in Owo. The difference in vaccine uptake by region was statistically significant (Pearson's Chi-squared test with Yates' continuity correction), where peri-urban study participants from Owo were more likely to be vaccinated than the urban participants from Ikorodu (Chi-square statistic=62.1, df=1, p value < 0.001). Among the individuals who reported sex, there was no statistically significant difference comparing the vaccinated and unvaccinated participants (Chi-square statistic = 3.1, df = 1, p value = 0.077). Among the individuals who reported education and religion, we found that there was a statistically significant difference comparing the vaccinated and unvaccinated participants, though marginally (education, Chi-square statistic = 8.5, df = 3, p value = 0.038); religion, Chi-square statistic = 8.0, df = 3, p value = 0.047). We then assessed whether there is an association between age and vaccine uptake. We found that, among our study population, increasing age was strongly associated with the likelihood of being vaccinated (logistic regression, age outcomes: β estimate = 0.047, p value < 0.001). For every 1-year increase in age, the odds of being vaccinated increased by ~4.8% (OR = 1.048, unadjusted for other factors). Note also that the percent of vaccinated individuals is higher in older age categories (Table 1). The

association between vaccine uptake and age also remained when adjusting for region (logistic regression, age outcomes: β estimate = 0.050, p value < 0.001). Since the COVID-19 vaccine campaigns covering our study period focused mostly on adults, we reran the analysis excluding children. The age-vaccine uptake association held true even when the analysis was restricted to those who were 18 years or older. Hence, increasing age was strongly associated with the likelihood of being vaccinated among adults (logistic regression, age outcomes: β estimate = 0.030, p value < 0.001), again not adjusting for other factors.

Comparing the presence of anti-SARS-CoV-2 antibodies between those who reported being vaccinated and those who were not vaccinated revealed that vaccinated participants had significantly more anti-RBD antibodies against the WT, Beta, Gamma, Lambda, and Omicron variants, though some differences were marginal (Figure 1). The most significant difference between the vaccinated and unvaccinated group was for RBD Beta (p=<0.0001). Interestingly, the anti-N WT antibody measurements were similar comparing the vaccinated and unvaccinated groups, suggesting that both groups had been exposed equally to natural infections prior to study begin (likely covering 1-2 years prior to study based on previously published antibody decay estimates (26,27).

Table 1. Percent and count of study participants by vaccination status in each sociodemographic and regional stratum.

	Category**	% Vaccinated (vacc*/total in strata)	p-value
Total Vacc*		66.6% (492/739)	
Region	Owo	80.2% (300/374)	<0.001
	Ikorodu	52.6% (192/365)	
Age	0-17 years	18.2% (12/66)	<0.001
	18-30 years	62.3% (137/220)	
	31-45 years	71.7% (198/276)	
	>45 years	82.4% (145/176)	
Sex	Male	58.9% (126/214)	0.077
	Female	66.4% (282/425)	
Education	No Education	100.0% (7/7)	0.038
	Primary	64.3% (83/129)	
	Secondary	65.2% (251/385)	
	University	74.4% (122/164)	
Religion	None	100.0% (1/1)	0.047
	Christianity	69.0% (391/567)	
	Islam	60.3% (82/136)	
	Other Religion	51.4% (18/35)	

*Vacc, vaccinated individuals (self-reported).

**Missing values: Region (n=6) missing data points for vaccine status. Age (n=7) missing data points for age or vaccine status. Sex (n=106) missing data points for sex or vaccine status.

Religion (n=6) missing data points for vaccine status. Education (n=60) missing data points for education or vaccine status.

Figure 1. **Anti-SARS-CoV-2 variant antibodies among vaccinated and unvaccinated study participants.** The graph shows raw median fluorescence intensity (MFI) outcomes by variant and the respective geometric means and 95% confidence intervals (CI). Vaccinated participants had significantly higher antibody levels against the wildtype (WT) RBD (p=0.013), RBD Beta (p=<0.0001), RBD Gamma (p=0.019), RBD Lambda (p=0.0007), and RBD Omicron (p=0.004) variants, but not RBD Alpha, RBD Delta, and WT Spike (S). There was no difference between vaccinated and unvaccinated study participants for the anti-Nucleoprotein (N) wildtype (WT, Wuhan) antibodies. Note, negative MFI values (artifact of the assay and not meaningful outcomes since there cannot be a negative value of antibodies in the samples) were set to 1 to plot the log(10) scale graph and better visualize the full spread of MFIs. Similarly, the few outcomes that ended up being 0 (n=3 study samples, Omicron only) were set to 1 for the same reasons. Vax, Vaccinated. MFI, median fluorescence intensity. RBD, receptor binding domain. Ns, not significant.

CoV Seroprevalence among Unvaccinated Study Participants

We utilized two methods to calculate the seroprevalence of SARS-CoV-2 variants in Owo (Ondo) and Ikorodu (Lagos) in Nigeria. Thus, we compared two different strategies to calculate thresholds and determine which samples are considered positive or negative according to the calculated cut-offs: (1) The first approach was a traditionally applied method where the threshold is set at three standard deviations above the mean of the negative controls (3STDV). (2) The second approach was based on a standardized open-source machine learning-based method that calculates sensitive and specific classification boundaries based on population-specific pre-validated positive and negative controls and gives individual and group-based uncertainty estimates for the seroprevalence outcomes (i.e., SeroNIST) (22–25).

Overall, the SARS-CoV-2 seroprevalence for anti-S and anti-RBD antibodies across all variants, except Omicron, was high (ranging from 89.1% to 100.0%, Table 2) and similar comparing the two methods among the unvaccinated study population. The seroprevalence for anti-RBD antibodies for the Omicron variant resulted in the most significant discrepancy and resulted in 82.2% (+/- 10.2 95% CI) for the SeroNIST method and 10.5% for the 3STDV method. Note that the SeroNIST method calculates thresholds by utilizing measurements from positive and negative controls (whereas the 3STDV approach is based on only the negative controls) and the positive controls were cases captured before Omicron circulated in the local population (see discussion for more details). The seroprevalence for anti-N WT antibodies resulted in 78.9% (+/- 7.7 95% CI) for the SeroNIST method and 66.4% for the 3STDV method.

Figure 2. Anti-SARS-CoV-2 variant antibodies among unvaccinated study participants (n=247). The graph shows raw median fluorescence intensity (MFI) outcomes by variant and the respective geometric means and 95% confidence intervals (CI). Note, negative MFI values (artifact of the assay and not meaningful outcomes since there cannot be a negative value of antibodies in the samples) were set to 1 to plot the log(10) scale graph and better visualize the full spread of MFIs. Similarly, the few outcomes that ended up being 0 (n=3 study samples, Omicron only) were set to 1 for the same reasons. MFI, median fluorescence intensity. S, Spike. RBD, receptor binding domain. N, Nucleoprotein.

Table 2. SARS-CoV-2 Seroprevalence among unvaccinated participants (n=247) by threshold assessment method (SeroNIST vs. 3STDV above the mean of negatives).

	SeroNIST	3STDV
	Seroprevalence % (95% CI)	Seroprevalence %
N WT	78.9 (+/- 7.7)	66.4
S WT	100 (+0/-3.4)	91.9
RBD WT	100 (+0/-8.9)	95.1
RBD Alpha	100 (+0/-7.0)	89.1
RBD Beta	100 (+0/-3.4)	96.0
RBD Gamma	100 (+0/-5.6)	97.2
RBD Delta	99.6 (+/- 3.2)	99.6
RBD Lambda	100 (+0/-2.8)	98.0
RBD Omicron	82.2 (+/- 10.2)	10.5

When breaking down N- and RBD WT-based seroprevalence by sociodemographic variables among unvaccinated study participants (using the SeroNIST method), the outcomes were similar comparing age groups, sex, education levels, and religious affiliation, see Table 3. Hence, the risk of infection (reflected by N- and RBD WT-based seroprevalence among unvaccinated individuals) seemed even and high across the evaluated sociodemographic variables.

Table 3. N- and RBD-based SARS-CoV-2 seroprevalence among unvaccinated study participants (n=247) by age, sex, education, and religion.

Category	Sub-group	Seroprevalence % (95 CI)	
		N WT	RBD WT
Age group	0-17 years	88.4 (+/- 15.0)	100.0 (+0/- 20.6)
	18-30 years	75.0 (+/- 13.7)	100.0 (+0/- 14.9)
	31-45 years	76.0 (+/- 14.0)	100.0 (+0/- 16.0)
	>45 years	79.5 (+/- 21.6)	100.0 (+0/- 25.4)
Sex	Male	70.2 (+/- 13.6)	100.0 (+0/- 15.1)
	Female	82.1 (+/- 9.8)	100.0 (+0/- 11.8)
Education	Primary	80.5 (+/- 17.6)	100.0 (+0/- 20.9)
	Secondary	78.0 (+/- 10.5)	100.0 (+0/- 12.2)
	University	91.8 (+/- 16.2)	100.0 (+0/- 22.1)
Religion	Christianity	82.6 (+/- 8.8)	100.0 (+0/- 10.7)
	Islam	73.5 (+/- 17.1)	100.0 (+0/- 19.2)

Note: The sample size was too small for missing categories, and "other religion" so they were omitted for this table/analysis.

When comparing the two cities based on the SeroNIST method, the SARS-CoV-2 seroprevalence for anti-RBD WT was 100% for both (Table 4). For the anti-N WT (single antigen) and anti-RBD/S/N WT (three antigens combined) antibodies, the seroprevalence was higher in Owo (96.1% and 100.0% respectively) compared to Ikorodu (71.6% and 95.5% respectively).

Table 4. SARS-CoV-2 seroprevalence among unvaccinated individuals by city with SeroNIST method.

	Seroprevalence % (95 CI)	
	Ikorodu	Owo
N WT (1D)	71.6 (+/- 9.7)	96.1 (+/- 11.3)
RBD WT (1D)	100.0 (+0/-10.8)	100.0. (+0/-16.4)
RBD S N WT (3D)	95.5 (+/- 7.5)	100.0 (+0/-5.2)

Discussion

The COVID-19 pandemic, caused by SARS-CoV-2, rapidly spread across the globe in 2019/2020, resulting in millions of deaths, overwhelming healthcare systems, disrupting economies, and exposing vulnerabilities in global public health (28,29). Surprisingly, the number of cases and fatalities in Africa was low compared to the rest of the world (30). Nigeria, Africa's most populous country, accounted for less than 1% of the global cases (2). Whether these numbers reflect the true burden of SARS-CoV-2 is yet to be elucidated. The present study evaluated serosurvey-based SARS-CoV-2 exposure, compared serological threshold assessment approaches, determined the risk factors for SARS-CoV-2 infection, and evaluated the COVID-19 vaccine uptake among febrile Southern Nigerian study participants across urban and peri-urban settings in 2022/2023. These study results offer insights into sociodemographic and regional risk factors for SARS-CoV-2 infection, strategies to optimize serological study design, and approaches to improve COVID-19 vaccine uptake in West Africa.

Study Population

We found slightly higher female involvement and most of the participants to fall into the young/middle-aged group (18–45 years), which aligns with previous studies showing greater engagement in public health research by women and socially active populations (31,32). This was also reflected in the educational status of the participants, where most people had received post-primary formal education.

Vaccine Uptake

Among those who reported vaccine status, the majority (66.6%) reported receiving a single or double dose of the COVID-19 vaccine, which is above the previously reported COVID-19 vaccine uptake in Nigeria, ranging from 20%-58% (33). While prior research indicates that sociodemographic factors significantly influence vaccine uptake (34,35)(36,37), we did not find sex, education, or religion to be strong indicators for COVID-19 vaccine uptake in our study

population. However, we found that peri-urban study participants from Owo were more likely to be vaccinated compared to urban counterparts from Ikorodu, indicating differential COVID-19 vaccine uptake across urban gradients in our study area. Since basic socioeconomic factors were not significant determinants of vaccine uptake in our study population, it may have been due to variation in COVID-19 vaccine distribution and accessibility or other factors our study did not capture. Furthermore, we found that older age was strongly associated with the likelihood of being vaccinated, likely reflecting the local vaccine campaign efforts during the study period, which focused on 60+ adults and 50+ adults with co-morbidities.

SARS-CoV-2 Serosurvey

Vaccinated participants had significantly more anti-RBD antibodies for the WT, Beta, Gamma, Lambda, and Omicron variants (Figure 1). Detecting more anti-S/RBD antibodies in the vaccinated group likely validated the self-reported vaccination status since (i) being vaccinated has been described to elicit a stronger anti-RBD/S antibody response than natural infection, and (ii) being both vaccinated and infected is likely to induce a higher antibody response than either alone (8,38). However, antibody decay should be taken into account (39,40), and we did not have information on time since vaccination or infection. Interestingly, the anti-N WT antibody measurements were similar comparing the vaccinated and unvaccinated groups, suggesting that both groups were exposed equally to natural infections prior to our study.

SARS-CoV-2 Serosurvey Among Unvaccinated Study Participants

We assessed SARS-CoV-2 seroprevalence among unvaccinated study participants to evaluate SARS-CoV-2 exposure and distinguish between vaccine- and natural infection-induced anti-RBD/S antibodies. Thus, the S/RBD and N-based seroprevalences likely represent natural infections since the analysis only included participants who reported not getting vaccinated. Our study revealed high S/RBD-based seroprevalences for all variants except Omicron among the unvaccinated participants (ranging from 89.1% to 100%),

indicating that the level of SARS-CoV-2 exposure was likely underestimated in Nigeria and confirming that reduced viral transmission was not likely the main reason the reported COVID-19 morbidity and mortality were low in Nigeria and the region (6). This is the highest seroprevalence rate for SARS-CoV-2 reported in Nigeria to date (11–16). The variation may reflect differences in sampling period and testing approach, advocating standardized serological methods. While most of the prior serosurveys were implemented between 2020 and mid-2022, our study happened later in the pandemic, between the end of 2022 and beginning of 2023. A similar high seroprevalence of over 90% was reported in Yemen in 2023 (41).

Furthermore, Nigerians may have been exposed to SARS-CoV-2 variants that were not previously reported in the country. Nigeria experienced four major SARS-CoV-2 waves dominated by the ancestral/WT, Alpha, Delta, Eta, and Omicron variants (17–20). Other important variants detected elsewhere, such as Gamma and Lambda variants, were not reported in Nigeria (42), but we detected relatively high levels of anti-RBD antibody for both variants among our study population, although some of the signal may have been due to cross-reactivity. While the affinity between the specific variant antigens (i.e., antigens coupled to the beads in our serological assay) and respective human antibodies should be the highest/tightest, we could not preclude cross-reactivity between SARS-CoV-2 variants and also with endemic human CoVs (e.g., HKU1, NL63, OC43, and 229E) due to lack of positive controls (i.e., individuals who were exposed only to specific SARS-CoV-2 variants, HKU1, NL63, OC43, or 229E). The low seroprevalence and antibody levels for Omicron RBD detected in our study may have been due to low transmission or due to a distinct serological footprint (43). Further genomic epidemiological approaches and proactive serosurveillance will help unravel the true burden of all SARS-CoV-2 variants in Nigeria.

As for risk factors for SARS-CoV-2 infection, we did not find high-risk sociodemographic subpopulations based on age, sex, educational status, or religion, similar to previous studies (11,31). When comparing RBD/S-based SARS-CoV-2 seroprevalence between Ikorodu and Owo, we also did not find a difference. When comparing N-based SARS-

CoV-2 seroprevalences, however, we found higher seroprevalence for Owo (96.1%) compared to Ikorodu (71.6%), which may reflect the timing of the most recent wave prior to the study beginning since the anti-N antibodies have a shorter half-life than RBD/S (44). Hence, since the RBD/S- and overall RBD/S/N-based seroprevalence is similar in both cities, there is likely no real difference in risk of exposure to SARS-CoV-2. Since the N-based seroprevalence is higher in Owo, that region may have experienced a more recent wave compared to Ikorodu, where the anti-N-based antibodies may already have decayed below the detection threshold for parts of the local population when we collected the samples.

Serological Threshold Assessment

Our study compared two threshold calculation methods to evaluate the SARS-CoV-2 seroprevalence. There was concordance between the two approaches, except for anti-RBD Omicron and anti-N antibodies. The difference between the N-based seroprevalence was within ~10% when considering the 95%CI, but the Omicron-based seroprevalence differed widely (82.2% vs. 10.5%). This was likely due to the positive controls, which were collected pre-Omicron, so they were not good controls to determine the SeroNIST-based thresholds for Omicron, which is based on positive and negative controls (whereas the 3STDV is based only on negative controls). Overall, the two methods ended up resulting in similar seroprevalence outcomes (except for Omicron, where no appropriate positive controls were available), and the SeroNIST method represents a standardized approach based on an open-source tool that provides uncertainty estimates for population-based and individual serological outcomes. Using a standardized approach across serosurveys (independent of the pathogen of interest) will make results comparable and suitable for metanalysis, increasing the public health impact of serosurveys in general.

Limitations

Our study results should be viewed considering a few limitations. For example, while we were able to enroll 745 study participants, increasing the limited sample size and including

additional socioeconomic variables would have given a more nuanced picture of risk factors for SARS-CoV-2 exposure and COVID-19 vaccine uptake. Furthermore, our study only covered symptomatic participants who visited local healthcare centers. Hence, it does not represent the general population. Further, individuals who visit health care centers when sick may be more open to engage with the public health system and therefore may be more likely to get vaccinated compared to the general population. The main limitation of our study was the inability to account for potential serological cross-reactivity between SARS-CoV-2 variants and potentially other CoVs due to the lack of positive controls for each CoV subtype.

Conclusion and Recommendations

(i) The results of this study suggest that the SARS-CoV-2 exposure in Southern Nigeria was higher than previously reported. Hence, lack of SARS-CoV-2 transmission was not likely the reason that Nigeria, and potentially the region, experienced less COVID-19 disease and related deaths. Still, genomic surveillance is needed to track variance-based exposure patterns (45). (ii) The COVID-19 vaccine uptake was high, with peri-urban participants showing greater coverage than those in urban areas, and increasing age was a strong predictor of vaccination. Hence, the local vaccine uptake strategy implemented prior to 2022/2023 proved effective, resulting in high coverage rates and representing a significant public health success. Ongoing public health interventions should focus on improving vaccine uptake in urban populations and younger age groups while leveraging the observed success of peri-urban vaccine campaigns. (iii) The lack of standardized serological thresholding methods highlights the need for standardized analytical approaches. While there was concordance between the two approaches, prioritizing the use of validated and standardized thresholding methods such as SeroNIST will enable reliable comparisons of serosurveys across studies and time, thereby increasing the public health impact of serosurveys.

Acknowledgements

We thank the study participants and their families for their valuable time and participation. Without them, this study would not have been possible.

Financial Support

Funding was provided by (1) the Centers for Disease Control and Prevention (CDC) U01GH002338-01 grant titled “Seroepidemiological Insight into COVID-19 Transmission in Africa (SICA)”, (2) the National Center for Advancing Translational Sciences KL2-TR001455 grant, and (3) the Bill and Melinda Gates Foundation GIISER Project (ARISE-INV-036304). The funders had no role in study design, data collection and analysis, decision to publish, or preparation of the manuscript.

Conflicts of Interest

The authors have declared that no competing interests exist.

References

1. Karcıoğlu O, Yüksel A, Baha A, Er AB, Esendağlı D, Gülhan PY, et al. Covid-19: The Biggest Threat of the 21st Century: In Respectful Memory of the Warriors All Over the World. Turk Thorac J. 2020 Dec 11;21(6):409–18.
2. WHO. COVID-19 Epidemiological Update. WHO; 2024.
3. Cabore JW, Karamagi HC, Kipruto HK, Mungatu JK, Asamani JA, Droti B, et al. COVID-19 in the 47 countries of the WHO African region: a modelling analysis of past trends and future patterns. Lancet Glob Health. 2022 Aug;10(8):e1099–114.
4. Maeda JM, Nkengasong JN. The puzzle of the COVID-19 pandemic in Africa. Science. 2021 Jan;371(6524):27–8.
5. Happi CT, Nkengasong JN. Two years of COVID-19 in Africa: lessons for the world.

6. Bejon P, Agweyu A, Oyier I, Hamaluba M, Kamuya D, Kinyanjui S, et al. Rethinking the Evidence on COVID in Africa. Lancet Infect Dis [Internet]. 2025 Apr 25 [cited 2025 June 21]; Available from: https://doi.org/10.1016/ S1473-3099(25)00071-4
7. Adams J, MacKenzie MJ, Amegah AK, Ezeh A, Gadanya MA, Omigbodun A, et al. The Conundrum of Low COVID-19 Mortality Burden in sub-Saharan Africa: Myth or Reality? Glob Health Sci Pract. 2021 Sept 30;9(3):433–43.
8. Ugwu CA, Alao O, John OG, Akinnawo B, Ajayi I, Odebode O, et al. Immunological insights into COVID-19 in Southern Nigeria. Front Immunol. 2024 Jan 23;15:1305586.
9. Ojo TO, Ojo AO, Ojo OE, Akinwalere BO, Akinwumi AF. Determinants of COVID-19 vaccine uptake among Nigerians: evidence from a cross-sectional national survey. Arch Public Health. 2023 May 26;81(1):95.
10. Ojeniyi T, Eguavoen A, Chinye-Nwoko F. Moving the needle for COVID-19 vaccinations in Nigeria through leadership, accountability, and transparency. Front Public Health. 2023 Sept 21;11:1199481.
11. Audu RA, Stafford KA, Steinhardt L, Musa ZA, Iriemenam N, Ilori E, et al. Seroprevalence of SARS-CoV-2 in four states of Nigeria in October 2020: A population-based household survey. Nelson MI, editor. PLOS Glob Public Health. 2022 June 17;2(6):e0000363.
12. Chechet GD, Kwaga JKP, Yahaya J, Noyes H, MacLeod A, Adamson WE. SARS-CoV-2 seroprevalence at urban and rural sites in Kaduna State, Nigeria, during October/November 2021, immediately prior to detection of the Omicron variant. Int J Epidemiol. 2022 Oct 13;51(5):1361–70.
13. Ige FA, Ohihoin GA, Osuolale K, Dada A, Onyia N, Johnson A, et al. Seroprevalence of SARS-CoV-2 IgG among healthcare workers in Lagos, Nigeria. Olu OO, editor. PLOS ONE. 2023 Oct 5;18(10):e0292440.
14. Kolawole OM, Tomori O, Agbonlahor D, Ekanem E, Bakare R, Abdulsalam N, et al. SARS CoV-2 Seroprevalence in Selected States of High and Low Disease Burden in Nigeria. JAMA Netw Open. 2022 Oct 11;5(10):e2236053.

15. Olayanju O, Bamidele O, Edem F, Eseile B, Amoo A, Nwaokenye J, et al. SARS-CoV-2 Seropositivity in Asymptomatic Frontline Health Workers in Ibadan, Nigeria. Am J Trop Med Hyg. 2021 Jan 6;104(1):91–4.
16. Akanmu S, Herrera BB, Chaplin B, Ogunsola S, Osibogun A, Onawoga F, et al. High SARS-CoV-2 seroprevalence in Lagos, Nigeria with robust antibody and cellular immune responses. J Clin Virol Plus. 2023 Aug;3(3):100156.
17. Olawoye IB, Oluniyi PE, Oguzie JU, Uwanibe JN, Kayode TA, Olumade TJ, et al. Emergence and spread of two SARS-CoV-2 variants of interest in Nigeria. Nat Commun. 2023 Feb 13;14(1):811.
18. Olorunfemi AB, Suliman SAR, Tran TT, Ayorinde B, Fowora MA, Iwalokun BA, et al. Whole genome sequencing and phylogenetic analysis of SARS-CoV-2 strains isolated during the COVID-19 pandemic in Nigeria. IJID Reg. 2024 Mar;10:174–8.
19. Kolawole DB, Okeke MI. Phylogenetic and genome-wide mutational analysis of SARS-CoV-2 strains circulating in Nigeria: no implications for attenuated COVID-19 outcomes. Osong Public Health Res Perspect. 2022 Apr 30;13(2):101–13.
20. Akande OW, Elimian KO, Igumbor E, Dunkwu L, Kaduru C, Olopha OO, et al. Epidemiological comparison of the first and second waves of the COVID-19 pandemic in Nigeria, February 2020–April 2021. BMJ Glob Health. 2021 Nov;6(11):e007076.
21. McConnell D, Hickey C, Bargary N, Trela-Larsen L, Walsh C, Barry M, et al. Understanding the Challenges and Uncertainties of Seroprevalence Studies for SARS-CoV-2. Int J Environ Res Public Health. 2021 Apr 27;18(9):4640.
22. Binder RA, Fujimor GF, Forconi CS, Reed GW, Silva LS, Lakshm P, et al. SARS-CoV-2 Serosurveys: How Antigen, Isotype and Threshold Choices Affect the Outcome. J Infect Dis. 2022 Oct 31;2023(227):371–80.
23. Binder RA, Matta AM, Forconi CS, Oduor CI, Bedekar P, Patrone PN, et al. Minding the margins: Evaluating the impact of COVID-19 among Latinx and Black communities with optimal qualitative serological assessment tools. Egorov AI, editor. PLOS ONE. 2024 July 25;19(7):e0307568.

24. Patrone PN, Binder RA, Forconi CS, Moormann AM, Kearsley AJ. Analysis of Diagnostics (Part I): Prevalence, Uncertainty Quantification, and Machine Learning [Internet]. arXiv; 2024 [cited 2025 June 21]. Available from: http://arxiv.org/abs/2309.00645
25. Patrone PN, Binder RA, Forconi CS, Moormann AM, Kearsley AJ. Analysis of Diagnostics (Part II): Prevalence, Linear Independence, and Unsupervised Learning [Internet]. arXiv; 2024 [cited 2025 June 21]. Available from: http://arxiv.org/abs/2408.16035
26. Terpos E, Stellas D, Rosati M, Sergentanis TN, Hu X, Politou M, et al. SARS-CoV-2 antibody kinetics eight months from COVID-19 onset: Persistence of spike antibodies but loss of neutralizing antibodies in 24% of convalescent plasma donors. Eur J Intern Med. 2021 July;89:87–96.
27. Xia W, Li M, Wang Y, Kazis LE, Berlo K, Melikechi N, et al. Longitudinal analysis of antibody decay in convalescent COVID-19 patients. Sci Rep. 2021 Aug 18;11(1):16796.
28. Faramarzi A, Norouzi S, Dehdarirad H, Aghlmand S, Yusefzadeh H, Javan-Noughabi J. The global economic burden of COVID-19 disease: a comprehensive systematic review and meta-analysis. Syst Rev. 2024 Feb 16;13(1):68.
29. Nicola M, Alsafi Z, Sohrabi C, Kerwan A, Al-Jabir A, Iosifidis C, et al. The socio-economic implications of the coronavirus pandemic (COVID-19): A review. Int J Surg. 2020 June;78:185–93.
30. Joseph S, Kutty Narayanan A. Covid 19-the 21st Century Pandemic: The Novel Coronavirus Outbreak and the Treatment Strategies. Adv Pharm Bull. 2021 Jan 30;1.
31. Onifade AA, Fowotade A, Rahamon SK, Edem VF, Yaqub SA, Akande OK, et al. Seroprevalence of anti-SARS-CoV-2 specific antibodies in vaccinated and vaccine naïve adult Nigerians. Bhattacharya D, editor. PLOS ONE. 2023 Jan 23;18(1):e0280276.
32. Wu C, Qian Y. The gender peak effect: Women are most vulnerable to infections during COVID-19 peaks. Front Public Health. 2022 Aug 9;10:937179.

33. Olu-Abiodun O, Abiodun O, Okafor N. COVID-19 vaccination in Nigeria: A rapid review of vaccine acceptance rate and the associated factors. Elelu N, editor. PLOS ONE. 2022 May 11;17(5):e0267691.

34. Tchiasso D, Mendjime P, Njuwa Fai K, Nana Wandji BS, Yuya F, Youm É, et al. Dynamic factors associated with COVID-19 vaccine uptake in Cameroon between 2021 and 2022. J Public Health Afr [Internet]. 2024 Oct 24 [cited 2024 Dec 8];15(1). Available from: https://publichealthinafrica.org/index.php/jphia/article/view/578

35. White TM, Wyka K, Rabin K, El-Mohandes A. Trust in the science behind COVID-19 vaccines as a driver of vaccine acceptance in the United States, 2021–2023. Vaccine X. 2024 Dec;21:100576.

36. Costa JC, Weber AM, Darmstadt GL, Abdalla S, Victora CG. Religious affiliation and immunization coverage in 15 countries in Sub-Saharan Africa. Vaccine. 2020 Jan;38(5):1160–9.

37. Santos TM, Cata-Preta BO, Wendt A, Arroyave L, Hogan DR, Mengistu T, et al. Religious affiliation as a driver of immunization coverage: Analyses of zero-dose vaccine prevalence in 66 low- and middle-income countries. Front Public Health. 2022 Oct 25;10:977512.

38. Kaplonek P, Deng Y, Shih-Lu Lee J, Zar HJ, Zavadska D, Johnson M, et al. Hybrid immunity expands the functional humoral footprint of both mRNA and vector-based SARS-CoV-2 vaccines. Cell Rep Med. 2023 May;4(5):101048.

39. Van Elslande J, Oyaert M, Lorent N, Vande Weygaerde Y, Van Pottelbergh G, Godderis L, et al. Lower persistence of anti-nucleocapsid compared to anti-spike antibodies up to one year after SARS-CoV-2 infection. Diagn Microbiol Infect Dis. 2022 May;103(1):115659.

40. Gallais F, Gantner P, Bruel T, Velay A, Planas D, Wendling MJ, et al. Evolution of antibody responses up to 13 months after SARS-CoV-2 infection and risk of reinfection. eBioMedicine. 2021 Sept;71:103561.

41. Taher WT, Bawazir AA, Sallam TA, Alsurimi K. Seroprevalence and factors associated with SARS-CoV-2 infection among healthcare workers: cross-sectional study. BMC Infect Dis. 2023 Nov 6;23(1):761.
42. Gozzi N, Chinazzi M, Davis JT, Mu K, Pastore Y Piontti A, Ajelli M, et al. Real-time estimates of the emergence and dynamics of SARS-CoV-2 variants of concern: A modeling approach. Epidemics. 2024 Dec;49:100805.
43. Qu P, Faraone JN, Evans JP, Zheng YM, Carlin C, Anghelina M, et al. Enhanced evasion of neutralizing antibody response by Omicron XBB.1.5, CH.1.1, and CA.3.1 variants. Cell Rep. 2023 May;42(5):112443.
44. Brlić PK, Pavletić M, Lerga M, Krstanović F, Matešić MP, Miklić K, et al. SARS-CoV-2 Spike and Nucleocapsid Antibody Response in Vaccinated Croatian Healthcare Workers and Infected Hospitalized Patients: A Single Center Cohort Study. Viruses. 2022 Sept 4;14(9):1966.
45. Happi CT, Nkengasong JN. Two years of COVID-19 in Africa: lessons for the world.

Supplemental Figure 1. Controls and 3STDV Threshold. The graph shows raw median fluorescence intensity (MFI) outcomes of the positive controls (red dots) and the negative controls (blue dots) by SARS-CoV-2 variant. In green are the 3STDV thresholds (based on negative controls only), with respective values on top. Note, negative MFI values (artifact of the assay and not meaningful outcomes since there cannot be a negative value of antibodies in the samples) were set to 1 to plot the log(10) scale graph and better visualize the full spread of MFIs. MFI, median fluorescence intensity. S, Spike. RBD, receptor binding domain. N, Nucleoprotein.

Supplemental Figure 2. SeroNIST Classification Boundary based on three Antibodies. (A) This figure shows the classification boundary (i.e., three-dimensional threshold in rainbow

colours) created by the SeroNIST fool for wildtype (WT) RBD, S, and N antibodies based on the training data (i.e., negative control in blue dots and positive controls in red dots). (B) This figure shows where the data points (blue dots) fall for an example data set of participants. In this case, all the data points fall above the classification boundary, resulting in an estimated SARS-CoV-2 seroprevalence of ~100% based on RBD, S, and N antibodies.

Click here to access/download;Figure;MFI by Vacc Status_Figure 1.pdf

Figure 1

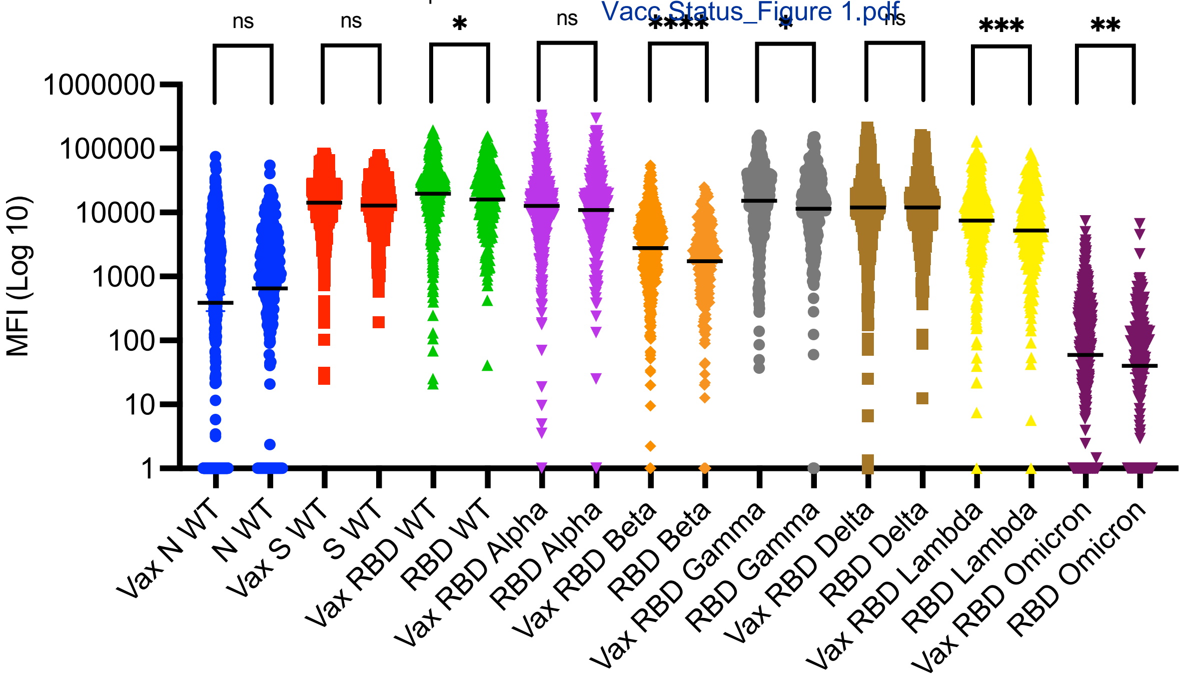

Figure 2

Click here to access/download;Figure;MFI NonVacc_Figure 2.pdf

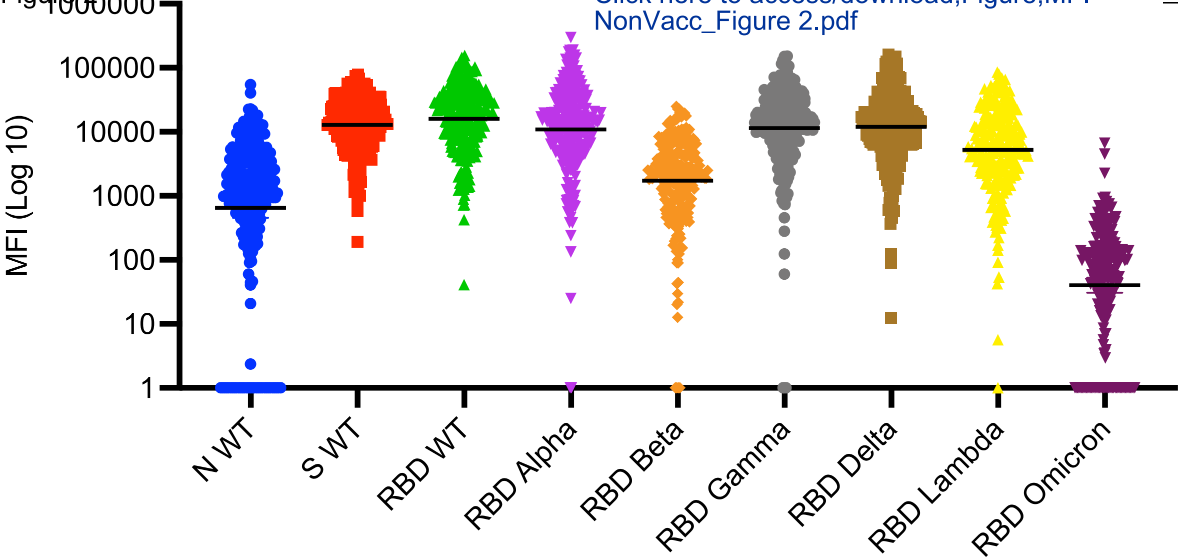

Supplemental Figure 1

Click here to access/download
Supporting Information
Controls 3STDV_Supplemental Figure 1.pdf

Supplemental Figure 2

Click here to access/download
Supporting Information
SeroNIST_Supplemental Figure 2.pdf

Database

Click here to access/download
Supporting Information
COVID Nigeria Dataset_Final_Aug 4 2025.xlsx
