# Supplementary material for: COVID19 Sero-epidemiology and vaccine uptake in two Southern Nigerian clinics
Source: PLOS Glob Public Health. 2026 Mar 27;6(3):e0006126. doi: 10.1371/journal.pgph.0006126 (PMC13029786; doi:10.1371/journal.pgph.0006126)
Supplement: Attachment — Submitted filename: Review Comments_PGPH-D-25-02354.pdf [file pgph.0006126.s021.pdf]

Reviewer comments: (Geofrey Basalirwa)

- **Comment 1:** In the results, improve the paragraph flow, clarity, and consistency to separate the total number of participants and where they we recruited with total in line 247 - 248.

- **Comment 2:** Correct the phrasing in line 249, (n=430, 57.7%) is not about half. That's more than half or a clear majority.

- **Comment 3:** Also correct the n totals for both Female and Male as indicated in Table 1, line 149

- **Comment 4:** The n total for missing sex (unreported is not 100), correct the n and the percentage on line 250

- **Comment 5:** Please state the reported average Age as mean line 250; also, both mean and SD can be reflected in Table 1 as well for clarity.

- **Comment 6:** Instead of reporting this **"while the majority of participants were between 18 and 45 years of age (n=497, 66.7%)"**. For more clarity to this paper, you could re-categorize Age as Minors, Young, Middle-aged aged and Elderly per the WHO standardization. Also, correct the n total (497) line 252.

- **Comment 7:** Correct the statistics for Religion for all categories as indicated in Table 1, ie, Christianity, Islam, etc., line 255.

- **Comment 8:** Paraphrase the wording in line 259 for improved flow and clarity.

- **Comment 9:** Categorize the age variable and be consistent with the categories across the narrative, ie, line 412 has **"young/middle-aged group (18–45 years)",** which is not consistent with the earlier write-up. Follow guidance like in the earlier comment

- **Comment 10:** Pair p-values with effect size (if possible) instead of only saying "statistically significant ($p < 0.05$)", you can include odds ratios (OR) or percentage point differences where applicable. (ie. Line 265 - 266, you would say, *"Participants from Owo were more likely to be vaccinated than those from Ikorodu (80.2% vs. 52.6%, $\chi^2 = 62.1$, df = 1, $p < 0.001$)."*

- **Comment 11:** Correct the result section, use "p = 0.038" instead of "p value = 0.038" for consistency with journal conventions, but not both interchangeably.

- **Comment 12:** Keep consistent decimal formatting across line 289, 314.

- **Comment 13:** Some ORs are reported in the narrative but have no originality. Please add the Odds ratios in the Table and in narratives.

- **Comment 14:** Bold line 120, 142, 162, 172, 201, 246, 257, 324, 410, 417, 432, 443, 487, 503, 515 as a subheading

- **Comment 15:** Please don't indent paragraphs. Kindly correct lines 86, 97, 108, 129, 135, 150, 205, 213, 285, 334, 459, 474. Consider triple spacing for new paragraphs.

- **Comment 16:** Have the figures be displayed visibly in the write-up up than having links to figures.

Comments and Suggestions for Authors

Dear Authors,

The study COVID-19 Sero-epidemiology and Vaccine Uptake in Two Southern Nigerian Clinics is certainly relevant and important. However, I have a few suggestions:

1. In the results, improve the paragraph flow, clarity, and consistency to separate the total number of participants and where they we recruited with the total in line 247 - 248.

2. Correct the phrasing in line 249, (n=430, 57.7%) is not about half. That's more than half or a clear majority.

3. Also correct the n totals for both Female and Male as indicated in Table 1, line 149.

4. The n total for missing sex (unreported is not 100), correct the n and the percentage on line 250.

5. Please state the reported average Age as mean line 250; also, both mean and SD can be reflected in Table 1 as well for clarity.

6. Instead of reporting this, ***"while the majority of participants were between 18 and 45 years of age (n=497, 66.7%)".*** For more clarity to this paper, you could re-categorize Age as Minors, Young, Middle-aged aged and Elderly per the WHO standardization. Also, correct the n total (497) line 252.

7. Correct the statistics for Religion for all categories as indicated in Table 1, ie, Christianity, Islam, etc., line 255.

8. Paraphrase the wording in line 259 for improved flow and clarity.

9. Categorize the age variable and be consistent with the categories across the narrative, ie, line 412 has "young/middle-aged group (18–45 years)", which is not consistent with the earlier write-up. Follow guidance like in the earlier comment.

10. Pair p-values with effect size (if possible) instead of only saying "statistically significant ($p < 0.05$)"; you can include odds ratios (OR) or percentage point differences where applicable. (i.e., Line 265 - 266, you would say, "Participants

from Owo were more likely to be vaccinated than those from Ikorodu (80.2% vs. 52.6%, $\chi^2 = 62.1$, df = 1, $p < 0.001$)."

11. Correct the result section, use "p = 0.038" instead of "p value = 0.038" for consistency with journal conventions, but not both interchangeably.

12. Keep consistent decimal formatting across line 289, 314.

13. Some ORs are reported in the narrative but have no originality. Please add the Odds ratios in the Table and in narratives.

14. Bold line 120, 142, 162, 172, 201, 246, 257, 324, 410, 417, 432, 443, 487, 503, 515 as a subheading.

15. Please don't indent paragraphs. Kindly correct lines 86, 97, 108, 129, 135, 150, 205, 213, 285, 334, 459, 474. Consider triple spacing for new paragraphs.

16. Have the figures be displayed visibly in the write-up up than having links to figures.

My main comment: Please ensure that the statistics reported in the results narrative correspond exactly to those in the tables. Use totals that exclude missing values (e.g., Table 1), and avoid referencing any statistics not presented in the tables. This consistency is critical for data accuracy.

Date of this review
20 OCT 2025
