# Supplementary material for: COVID19 Sero-epidemiology and vaccine uptake in two Southern Nigerian clinics
Source: PLOS Glob Public Health. 2026 Mar 27;6(3):e0006126. doi: 10.1371/journal.pgph.0006126 (PMC13029786; doi:10.1371/journal.pgph.0006126)
Supplement: Attachment — Submitted filename: PGPH-D-25-02354_R1_reviewer.pdf [file pgph.0006126.s023.pdf]

PLOS Global Public Health

COVID19 Sero-epidemiology and Vaccine Uptake in Two Southern Nigerian Clinics

--Manuscript Draft--

**Manuscript Number:**	PGPH-D-25-02354R1
**Article Type:**	Research Article
**Full Title:**	COVID19 Sero-epidemiology and Vaccine Uptake in Two Southern Nigerian Clinics
**Short Title:**	COVID-19 and Vaccine Uptake in Southern Nigeria
**Corresponding Author:**	Raquel A. Binder, PhD, MPH<br>University of Massachusetts Chan Medical School<br>Worcester, MA UNITED STATES OF AMERICA
**Order of Authors:**	Chinedu A. Ugwu
	Ooreofe Odebode
	Israel O. Ajayi
	Josiah Olubunmi
	Bejide Ifeoluwa
	Jolly A. Adole
	Kazeem Akano
	Johnson Okolie
	Philomena Eromon
	Imonikhe K. Kio
	Judith Amadi
	Precious F. Adebayo
	Iheanyi Mgbeokwere
	Akeem O. Lawal
	Olayemisi A. Adegoke
	Amoke O. Adeyemi
	Sampson Owhin
	Oladele O. Ayodeji
	Alebiosu Ahmed
	Etim Henshaw
	Oyejide E. Nicholas
	Abah A. Sylvester
	Abiola Omidele
	Kolade Emilola
	Olayiwola Funmilayo
	Adeboye Rufus Oyewale
	Oluwatuyi O. Olakunle
	Obaado Oluwaseun
	Angela M. Matta
	Prajakta Bedekar

Powered by Editorial Manager® and ProduXion Manager® from Aries Systems Corporation

	Latifeh Dahmash
	Catherine S. Forconi
	Ann M. Moormann
	Rhoel R. Dinglasan
	Christian T. Happi
	Raquel A. Binder, PhD, MPH
**Keywords:**	COVID-19, Nigeria, Serosurvey, Vaccine Uptake, Threshold method
**Abstract:**	Background: Africa has reported lower COVID-19 morbidity and mortality compared to other regions. Yet, the flat COVID-19 trajectory remains to be fully explained. Nigeria, Africa's most populous country, accounted for less than 1% of global COVID-19 cases. The present study evaluated SARS-CoV-2 seroprevalence, vaccine uptake, and methodological differences in serological threshold setting among febrile patients attending peri-urban and urban clinics in Southern Nigeria. Methods: Between October 2022 and February 2023, 745 febrile participants were recruited from Owo (Ondo State) and Ikorodu (Lagos State). Serum samples were tested for IgG antibodies against wild-type and variant SARS-CoV-2 antigens using a multiplex bead-based antibody assay. Seroprevalence was calculated using two classification boundary methods: (i) the conventional three standard deviations above the mean of negative controls (3STDV) and (ii) the open-source SeroNIST tool. Vaccine uptake and risk factors for exposure were assessed. Results: Vaccine uptake was 66.0% and significantly higher in peri-urban Owo (80.2%) than urban Ikorodu (52.6%, p-value < 0.0001). Increasing age was positively associated with vaccination (p-value < 0.0001). Sex, education, and religion did not influence vaccine uptake. Vaccinated individuals had significantly lower odds of being positive for anti-N SARS-CoV-2 antibodies (p-value = 0.001), indicating a potential protective effect of the vaccine. Age, education, and religion did not influence anti-N SARS-CoV-2 seropositivity. Overall, our study revealed high S/RBD-based seroprevalences for all variants except Omicron among the unvaccinated participants (range: 89.1% - 100%). Conclusions: SARS-CoV-2 exposure in Southern Nigeria was likely widespread and underrecognized, with high seroprevalence among symptomatic peri-urban and urban populations. Peri-urban communities demonstrated higher vaccine uptake than urban counterparts, and age-targeted campaigns likely drove uptake among older individuals. Further, our study detected a potential protective effect of the vaccine among the study population. Finally, standardized thresholding methods such as SeroNIST are critical for accurate seroprevalence estimates.
**Additional Information:**	
**Question**	**Response**
**Financial Disclosure**<br><br>Enter a financial disclosure statement that describes the sources of funding for the work included in this submission and the role the funder(s) played. This includes grants and any commercial funding of the work or authors.<br><br>This statement will be typeset if the manuscript is accepted for publication.<br><br>*Please review the submission guidelines*	Funding was provided by (1) the Centers for Disease Control and Prevention (CDC) through the U01GH002338-01 grant (RRD, CTH, and AMM) titled "Seroepidemiological Insight into COVID-19 Transmission in Africa (SICA)", (2) the National Institute of Health (NIH) through the National Center for Advancing Translational Sciences KL2-TR001455 grant (RAB), and (3) the Bill and Melinda Gates Foundation through the GIISER Project ARISE-INV-036304 (CTH and CAU). The funders had no role in study design, data collection and analysis, decision to publish, or preparation of the manuscript.

Powered by Editorial Manager® and ProduXion Manager® from Aries Systems Corporation

and the instructions link below for detailed requirements and guidance.	
**Competing Interests**<br><br>On behalf of all authors, disclose any competing interests that could be perceived to bias this work.<br><br>This statement will be typeset if the manuscript is accepted for publication.<br><br>*Please review the instructions link below and PLOS Global Public Health's competing interests policy to determine what information must be disclosed at submission.*	The authors have declared that no competing interests exist.
**Data Availability**<br><br>Before publication, Authors are required to make fully available and without restriction all data underlying their findings. Please see our PLOS Data Policy page for detailed information on this policy.<br><br>A **Data Availability Statement**, detailing where the data can be accessed, is required at first submission. Insert your Data Availability Statement in the box below.<br><br>Please see the data reporting section of our submission guidelines for instructions on what you need to include in your Data Availability Statement.<br><br>This statement will be typeset if the manuscript is accepted for publication.<br><br>*PLOS allows rare exemptions to address legal and ethical concerns. If you have legal or ethical restrictions, please use the*	The full database has been submitted.

Powered by Editorial Manager® and ProduXion Manager® from Aries Systems Corporation

box below to detail these in full sentences for the Journal team to consider.	

Manuscript

Click here to access/download;Manuscript;SICA ACEGID Manuscript_12 17 25_clean.docx

COVID19 Sero-epidemiology and Vaccine Uptake in Two Southern Nigerian Clinics

Chinedu A. Ugwu[1,2], Ooreofe Odebode[1,2], Israel O. Ajayi[1,2], Josiah Olubunmim[1,2], Bejide Ifeoluwa[1,2], Jolly A. Adole[1,2], Kazeem Akano[1,2], Johnson Okolie[1], Philomena Eromon[1], Imonikhe K. Kio[1], Judith Amadi[1], Precious F. Adebayo[1], Iheanyi Mgbeokwere[1], Akeem O. Lawal[3], Olayemisi A. Adegoke[3], Amoke O. Adeyemi[1], Sampson Owhin[4], Oladele. O. Ayodeji[4], Alebiosu Ahmed[1], Etim Henshaw[1], Oyejide E. Nicholas[1], Abah A. Sylvester[1,4], Abiola Omidele[1,4], Kolade Emilola[1], Olàyiwolà Funmilayo[1], Adeboye Rufus Oyewale[1], Oluwatuyi O. Olakunle[1], Obaado Oluwaseun[1], Angela M. Matta[5], Prajakta Bedekar[6,7], Latifeh Dahmash[8], Catherine S. Forconi[5], Ann M. Moormann[5], Rhoel R. Dinglasan[8], Christian T. Happi[1,2], Raquel A. Binder[5*]

Affiliations

[1]The Africa Centre of Excellence for Genomics of Infectious Diseases (ACEGID), Redeemer's University Nigeria, Ede, Nigeria.

[2]Department of Biological Sciences, Faculty of Natural Sciences, Redeemer's University, Ede, Osun State Nigeria.

[3]Ikorodu General Hospital Ikorodu, Lagos State Nigeria

[4]Federal Medical Centre Owo, Ondo State Nigeria

[5]Department of Medicine, University of Massachusetts Chan Medical School, Worcester, MA, USA

[6]Department of Applied Mathematics and Statistics, Johns Hopkins University, Baltimore, MD, USA

[7]Department of Mathematics, BITS Pilani, Hyderabad, Telangana, India

[8]Department of Infectious Diseases & Immunology, College of Veterinary Medicine, University of Florida, Gainesville, FL, USA

***Corresponding author**: raquel.binder@umassmed.edu

Running title: COVID-19 and Vaccine Uptake in Southern Nigeria

Abstract word count: 298/300

Main text word count: 5,321

Abstract

Background: Africa has reported lower COVID-19 morbidity and mortality compared to other regions. Yet, the flat COVID-19 trajectory remains to be fully explained. Nigeria, Africa's most populous country, accounted for less than 1% of global COVID-19 cases. The present study evaluated SARS-CoV-2 seroprevalence, vaccine uptake, and methodological differences in serological threshold setting among febrile patients attending peri-urban and urban clinics in Southern Nigeria. Methods: Between October 2022 and February 2023, 745 febrile participants were recruited from Owo (Ondo State) and Ikorodu (Lagos State). Serum samples were tested for IgG antibodies against wild-type and variant SARS-CoV-2 antigens using a multiplex bead-based antibody assay. Seroprevalence was calculated using two classification boundary methods: (i) the conventional three standard deviations above the mean of negative controls (3STDV) and (ii) the open-source SeroNIST tool. Vaccine uptake and risk factors for exposure were assessed. Results: Vaccine uptake was 66.0% and significantly higher in peri-urban Owo (80.2%) than urban Ikorodu (52.6%, p-value < 0.0001). Increasing age was positively associated with vaccination (p-value < 0.0001). Sex, education, and religion did not influence vaccine uptake. Vaccinated individuals had significantly lower odds of being positive for anti-N SARS-CoV-2 antibodies (p-value = 0.001), indicating a potential protective effect of the vaccine. Age, education, and religion did not influence anti-N SARS-CoV-2 seropositivity. Overall, our study revealed high S/RBD-based seroprevalences for all variants except Omicron among the unvaccinated participants (range: 89.1% - 100%). Conclusions: SARS-CoV-2 exposure in Southern Nigeria was likely widespread and underrecognized, with high seroprevalence among symptomatic peri-urban and urban populations. Peri-urban communities demonstrated higher vaccine uptake than urban counterparts, and age-targeted

campaigns likely drove uptake among older individuals. Further, our study detected a potential protective effect of the vaccine among the study population. Finally, standardized thresholding methods such as SeroNIST are critical for accurate seroprevalence estimates.

Key words: COVID-19, Nigeria, Serosurvey, Vaccine Uptake, Threshold method

Introduction

Severe acute respiratory syndrome coronavirus 2 (SARS-CoV-2), causing coronavirus disease 2019 (COVID-19), was first identified in Wuhan, China, in December of 2019 and has since rapidly disseminated across the globe, causing the biggest pandemic of the 21st century (1). Close to 800 million cases and around 7 million deaths have been attributed to COVID-19, which is likely underestimating the true global burden (2). Surprisingly, Africa experienced a less catastrophic outbreak compared to the rest of the world, accounting for less than 2% of the global cases (3). Nigeria, the most populated country in Africa, accounted for just 0.003% of the global cases (2). At first, limited testing, a younger population, reduced transmission, preexisting immunity, genetic protective factors, early implementation of public health measures, and timely leadership were proposed to drive the flat COVID-19 trajectory (4,5). More recent evidence suggests that reduced viral transmission and poor surveillance were not the main reasons the reported COVID-19 morbidity and mortality were low (6). Instead, the population age structure and a reduced anti-SARS-CoV-2 inflammatory response due to specific immunoregulation or cross-reactive pre-pandemic immunity are likely the main protective factors (6). Similar factors were likely the reason a low number of COVID-19 cases and related deaths were reported in Nigeria (7,8). To better understand the extent of existing immunity to SARS-CoV-2 in Nigeria and risk factors of infection, we implemented a study measuring seroprevalence against different variants of SARS-CoV-2 between October 2022 and February 2023 in two Southern Nigerian clinics covering peri-urban and urban settings. Given the varying vaccine availability and acceptability in the region, we also evaluated the COVID-19 vaccine uptake. At the time of the study, the following vaccines were approved for

emergency use in Nigeria and available in our study population: Vaxveria (Oxford/ AstraZeneca), Covishield (Serum Institute of India), Comirnaty (Pfizer/BioNTech), Jcovden (Johnson and Johnson), Spikevax (Moderna), Sputnik V (Gamaleya). Note, Covilo (Sinopharm) was approved but not used in our study population. By 2023, ~110 million doses of COVID-19 vaccines had been distributed and ~30% of the population had full vaccine coverage (9,10).

Sero-epidemiological studies (serosurveys) evaluate pathogen-specific antibodies, thus serving as a tool to evaluate the burden of infection and existing immunity among populations. Serosurveys are particularly valuable in contexts where clinical reporting and molecular testing for infectious diseases are constrained (11). There are limited SARS-CoV-2 serosurveys implemented in Nigeria, and most of them focused on health care workers or only assessed the ancestral SARS-CoV-2 strain (i.e., wildtype [WT] or Wuhan) (11–16). Between February 2020 and November 2022, Nigeria had experienced four major COVID-19 waves, which were dominated by the wildtype (WT)/ancestral, Alpha, Delta, Eta, and Omicron variants (17–20). Other important variants detected elsewhere, such as Gamma and Lambda, were not reported in Nigeria. Whether this means these variants did not circulate in Nigeria or whether they went unreported is unclear.

While serosurveys are essential public health tools, the applied downstream analysis methods to convert quantitative outcomes (e.g., median fluorescence Intensity [MFI] values from bead-based assays or optical density [OD] values from enzyme-linked immunosorbent assays [ELISAs]) into qualitative results (positive/negative) vary greatly, making metanalyses and comparisons of outcomes across studies and regions challenging (21,22). Here, we applied a validated weighted-standard curve across-plate normalization method (23) and compared two classification boundary methods for optimal qualitative serological assessments: (1) The traditionally applied three standard deviations above the mean of the negative controls (3STDV), and (2) an open-source machine learning-based tool that calculates sensitive and

specific classification boundaries based on population-specific positive and negative controls, which has been termed SeroNIST (22–25).

In summary, we implemented a 2022/2023 SARS-CoV-2 serosurvey in Southern Nigeria based on standardized serological tools, determined the risk factors for infection, and evaluated the COVID-19 vaccine uptake among symptomatic individuals visiting local health care centers. Understanding the sero-epidemiological dynamics of the disease and contrasting peri-urban and urban settings is crucial for assessing the population's exposure, immunity levels, and potential risk factors. By examining the SARS-CoV-2 seroprevalence in Southern Nigeria, this study bridged knowledge gaps about regional risk factors for infection and population-level exposures. Finally, assessing COVID-19 vaccine uptake allowed us to evaluate coverage and inform future public health strategies aiming to increase vaccine uptake.

Methods

Study Clinics

The study was conducted at two clinical sites in Southern Nigeria: (1) The Federal Medical Centre, Owo (FMC Owo), in Ondo State from October 19 to November 22, 2022, and (2) the General Hospital, Ikorodu (Ikorodu GH) in Lagos State from November 14, 2022, to February 15, 2023. These sites were selected based on their capacity to support surveillance research among febrile populations and their existing clinical diagnostic and research infrastructure. Both facilities serve large catchment populations and represent diverse epidemiological settings, covering both peri-urban (Owo) and urban (Ikorodu) sites and therefore offering a unique opportunity to assess SARS-CoV-2 exposure and COVID-19 vaccine uptake. Additional information regarding the ethical, cultural, and scientific considerations specific to inclusivity in global research is included in the Supporting Information, see Supplemental Checklist.

FMC Owo is a federal tertiary healthcare institution with peri-urban extension services and a fully functional laboratory run by the Africa Centre of Excellence for Genomics of Infectious Diseases (ACEGID). The facility supports a wide range of diagnostic capabilities and biological sample processing. Ikorodu GH is a high-volume secondary health facility serving an urban population just outside metropolitan Lagos, with clinical services and infrastructure well-suited to the project's needs.

Both sites provided centralized coordination of participant enrolment, data collection, and sample processing. Field teams were trained in Good Clinical Practice (GCP), biosafety procedures, and the use of standardized data collection tools. Community engagements were carried out at both sites to ensure culturally appropriate and voluntary participation. The study design ensured contextual relevance, operational feasibility, and scientific rigor for characterizing the SARS-CoV-2 epidemiology and COVID-19 vaccine uptake in distinct healthcare settings within southern Nigeria.

Human Subjects

The study protocol, informed consent procedures, and all associated study tools were reviewed and approved by the Nigerian National Health Research Ethics Committee (NHREC) and the Health Research Ethics Committees (HRECs) of Owo FMC (NHREC/01/01/2007-31/08/2021). Similarly, the University of Florida Institutional Review Board (IRB) reviewed and approved the study and associated documents (IRB202102308). Ethical oversight covered all aspects of study implementation, including participant recruitment, data collection, and biological sampling.

Inclusion criteria: febrile individuals across all ages seeking care at either study clinic. All participants provided written informed consent prior to enrolment. For participants under the age of 18, assent was obtained in conjunction with parental or guardian consent. Trained field personnel conducted the consent process using IRB-approved forms. The consent process

clearly outlined the study objectives, procedures, potential risks, and the voluntary nature of participation. Participants were informed of their right to withdraw from the study at any time without penalty. Confidentiality was maintained throughout the study. Each participant was assigned a unique identifier, which was used to label all data and specimens. Electronic data were entered into encrypted REDCap databases hosted on password-protected devices. To mitigate risks, field staff were trained in infection prevention and control practices and used personal protective equipment during all participant interactions. These measures ensured ethical standards were upheld while maximizing participant safety and study integrity.

Sample and Data Collection

Participant recruitment occurred through the outpatient departments of both hospitals. Individuals presenting with acute febrile illness were screened for eligibility (i.e., consenting abilities) and invited to participate. Post-consent, a brief survey (capturing basic demographics and COVID-19 vaccine uptake) and a blood sample were collected. Each participant provided finger-prick whole blood through an EDTA microtainer tube. Serum was separated onsite using portable centrifuges and stored -20 °C under cold-chain conditions. Each sample was labelled with a unique study identifier and linked to participant data through encrypted REDCap databases. Stored and processed samples were transported from the site using a dry shipper (pre-charged to -20 °C) to the ACEGID laboratory at Redeemer's University for bead-based multiplex serology (Luminex MagPix).

***Serology* - Multiplex Bead-based Antibody Assay**

A bead-based IgG SARS-CoV-2 multiplex assay was implemented as previously described (23). Briefly, wild-type (WT; Wuhan) full-length spike (S), WT nucleocapsid (N), WT receptor-binding domain (RBD), RBD Alpha, RBD Beta, RBD Gamma, RBD Delta, RBD Lambda, and RBD Omicron SARS-CoV-2 antigens were coupled to MagPlex microspheres (Luminex Corporation). Bovine Serum Albumin (BSA) coupled beads were used to control for bead-specific background noise. All coupled beads were diluted in ABE buffer (PBS, BSA 0.1%,

TWEEN 20%, Sodium Azide 0.05%) to make a master mix of all the coupled antigens (consistent concentration across all beads, 1,000,000 beads/mL). Then, the master mix was added to each well of a 96-well plate and placed on a magnet to allow the beads to settle and remove the ABE buffer. Subsequently, 50 µL of human serum samples diluted (1:100) in ABE buffer were added to the wells, followed by a 2-hour incubation period on a plate shaker (300 rpm). Next, the beads were washed three times with 125 µL ABE buffer while plate remained on the magnet. Then 50 µL of biotinylated anti-human secondary IgG (BD Pharmingen) diluted in ABE (1:1000) was added to each well and followed by a one-hour incubation at room temperature on a plate shaker (300 rpm). The beads were once again washed three times, and 50 µL of phycoerythrin-conjugated streptavidin (1:1000; BD Pharmingen catalog # 554061) diluted in ABE added to the wells. Next, the beads were set on the plate shaker at 300 rpm for 15 minutes. After the final incubation period, the beads were washed 3 times for the last time and resuspended in 125 µL of ABE. After resuspension, the plate was read on the Luminex MagPix instrument, where a median fluorescence intensity (MFI) value was generated for each sample and individual antigen with the corresponding bead count. Once the control MFIs (positive and negative controls), standards (dilution of positive control), and bead counts (minimum 50 beads per antigen in each well) were validated, BSA was subtracted (including for the standards) to account for non-specific binding. Next, we applied an across-plate normalization factor based on the standards and transformed the MFIs into qualitative (positive and negative outcomes) as previously described (22–25). Previously collected de-identified banked blood/serum samples served as negative (n=36) and positive (n=21) controls (8).

Statistics

Statistical calculations and graphs were completed in R Studio (version 2024.09.0+375), GraphPad Prism (version 10.5.0), and the open-source SeroNIST tool (available at following link: https://github.com/SeroNISTPI/SeroNIST).

Frequency and percentages were used to describe the sociodemographic descriptors and vaccine uptake among the study participants. The Pearson's Chi-squared test with Yates' continuity correction was used to assess differences in vaccine up-take based on sociodemographic variables and by region, while excluding missing data. The multivariable analysis/logistic regression for COVID-19 vaccine uptake and serology (positive or negative for anti-N SARS-CoV-2 antibodies) were run in R Studio with the glm function (26). For the vaccine uptake analysis, the missing data points for vaccine uptake (n=6, 0.8%) were excluded since a binary logistic regression requires an outcome/dependent variable with binary outcomes. For the serological analysis, the missing data points for vaccine uptake (n=6, 0.8%) were included. The following variables also had missing data: sex (n=100, 13.4%) and education (n=54, 7.2%), which were included as separate categories in all the regression analyses. Age had a single missing data point (n=1), which was not included in any of the models due to the small sample size. The resulting estimates were used to calculate adjusted Odds Ratios (aOR) and 95% confidence intervals (95% CI) which are all listed in the Supplemental Tables.

To visualize anti-SARS-CoV-2 WT and variant antibodies among vaccinated and unvaccinated study participants, raw median fluorescence intensity (MFI) and respective geometric mean and 95% CI were plotted. The geometric mean (vs. standard average) was applied to minimize the effect of outliers. Differences between vaccinated and unvaccinated groups for each SARS-CoV-2 WT and variants were assessed with the unpaired nonparametric Mann–Whitney U test (a.k.a. Wilcoxon rank-sum test). Note, while the graph in Figure 2 is in Log(10) scale to visualize the MFI spread, the statistical test was applied to raw/non-transformed numbers.

We utilized two methods to calculate the seroprevalence of SARS-CoV-2 WT and variants: (1) The first approach was a traditionally applied method where the mean and standard deviation

(STDV) of the negative controls is calculated and then the STDV multiplied by 3 and added to the mean (mean+3*STDV=3STDV threshold) and set as cut off. This method assumes the reads of the negative controls are normally distributed. Since the MFIs of our negative controls were skewed (i.e., not normally distributed data) we attempted to log transformed as previously described (22). However, log(10) transforming the MFI values of the negative controls for each antigen/isotype skewed the distribution even more, so the raw values were used to calculate the 3SDTV, likely underlining the inadequacy of this method for other data sets since negative controls will tend to accrue around zero or the lower limit of detection of the applied assay. Note, negative MFI values (artifact of the assay and not meaningful outcomes since there cannot be a negative value of antibodies in the samples) were set to 1. Similarly, values of zero (n=6) were set to 1 to be able to show the spread of the data in log(10). See Supplemental Figure 1 for the raw MFI of the negative and positive controls, along with the calculated 3STDV thresholds. (2) The second approach is a standardized open-source machine learning-based method that calculates 1-, 2-, and 3-dimensional classification boundaries based on confirmed positive and negative controls, as previously described (i.e., SeroNIST) (20–23). Each dimension corresponds to the number of antigens included, (i.e., equivalent to 1, 2, or 3 antigens). For example, 3 antigens covered WT RBD, N, and S to estimate SARS-CoV-2 seroprevalence, see Supplemental Figure 2. The resulting classification boundary is used as threshold, same as for the 3STDV method. For the SeroNIST method, raw MFI values were used (no adjustment for negative values or zeros necessary). The serological platform was evaluated, validated, and used for SARS-CoV-2 seroprevalence studies prior to this, see following references (23–25).

Results

Study Population and Demographics

A total of 745 participants were recruited from Owo in Ondo State (n=375) from October to November 2022 and from Ikorodu in Lagos State (n=370) from November 2022 to February 2023. Over half of the participants were female (n=430, 57.7%), while 28.9% (n=215) were

male, and 13.4% (n=100) did not report sex. Ages ranged from 0 to 84 years (mean: 35.1 years, STDV: 14.7 years). The majority of participants had a secondary education (n=389, 52.2%), followed by those with university education (n=164, 22.0%), and then those with primary school education (n=130, 17.4%). A total of 54 individuals (7.2%) did not report education. Christianity was the dominant self-reported religion among our participants (n=570, 76.5%), followed by Islam (n=138, 18.5%), and those who practised other religions (n=36, 4.8%). Only one person reported not practicing any religion (0.1%). These sociodemographic variables are also described in Supplemental Table 1 through Supplemental Table 5.

Vaccine Uptake

The majority of participants reported having received either a single or double dose of the COVID-19 vaccine (n=492, 66.0%), while 247 (33.2%) reported not being vaccinated, and six participants (0.8%) opted out of reporting COVID-19 vaccine uptake, see Supplemental Table 6. The majority of the participants from Owo (peri-urban, Ondo State) were vaccinated (n=300, 80.2%, Supplemental Table 7). The vaccine uptake in Ikorodu (urban, Lagos State) was also relatively high (n=192, 52.6%) but lower than in Owo. The difference in regional vaccine uptake was statistically significant (Pearson's Chi-squared test with Yates' continuity correction), where peri-urban study participants from Owo were more likely to be vaccinated than the urban participants from Ikorodu (Chi-square statistic=62.1, df=1, p-value < 0.001). To determine whether sex, age, education, religion, state of residency, or being positive for anti-N SARS-CoV-2 antibodies independently increased or decreased the odds of having received a COVID-19 vaccine, we ran a multivariable analysis (i.e., logistic regression). To converge the model, religion and education categories with very small sample sizes (n=1 for no religion and n=8 for no formal education) were collapsed into larger categories for this analysis, see Supplemental Table 8 and Supplemental Table 9. Residency (Owo), age, and being positive for anti-N WT SARS-CoV-2 antibodies were all independently statistically significant and thus associated with vaccination status, see Figure 1 and Supplemental Table 10. Hence,

individuals from Owo (aOR = 3.9, p-value < 0.0001) and older study participants (aOR = 1.1, p-value < 0.0001) had significantly higher odds of being vaccinated. Participants positive for anti-N SARS-CoV-2 antibodies (aOR = 0.5, p-value < 0.002) were much less likely to be vaccinated than participants negative for anti-N WT SARS-CoV-2 antibodies. We reran the analysis including age as categorial variable to confirm these outcomes, see Supplemental Table 11. Residency (Owo), increased age, and being positive for anti-N WT SARS-CoV-2 antibodies were statistically significant again, see Supplemental Table 12. Individuals who were missing sex descriptors were also statistically significant, but the effect was barely significant (p-value = 0.043) and only observed when age was included as categorical variable. Thus, in summary, residents from Owo and study participants of older age had much higher odds of being vaccinated than residents from Ikorodu and study participants below the age 15. Further, individuals who had anti-N SARS-CoV-2 antibodies (reflecting natural infection) had lower odds of being vaccinated than to those who were negative for anti-N SARS-CoV-2 antibodies. Finally, sex, education, and religion did not influence vaccine uptake in our analysis.

Figure 1. Adjusted odds ratios for predictors of COVID-19 vaccine uptake. The figure shows adjusted odds ratios (aORs) for vaccine uptake in log scale by evaluated predictors (state, age, sex, education, religion, and anti-N SARS-CoV-2 seropositivity). Owo residency and age were strongly associated with higher odds of vaccination (p-value < 0.0001), while seropositivity had lower odds of vaccination (p-value = 0.002). Sex, education, and religion were not significantly associated with vaccine uptake.

Comparing the presence of anti-RBD SARS-CoV-2 antibodies between those who reported being vaccinated and those who were not vaccinated revealed that vaccinated participants had significantly more anti-RBD antibodies against the WT, Beta, Gamma, Lambda, and

Omicron variants (Figure 2). The most significant difference between the vaccinated and unvaccinated group was for RBD Beta (p-value < 0.0001).

Figure 2. **Anti-SARS-CoV-2 variant antibodies among vaccinated and unvaccinated study participants.** The graph shows raw median fluorescence intensity (MFI) outcomes by variant and the respective geometric means and 95% confidence intervals (CI). Vaccinated participants had significantly higher antibody levels against the wildtype (WT) RBD (p-value = 0.013), RBD Beta (p-value < 0.0001), RBD Gamma (p-value = 0.019), RBD Lambda (p-value = 0.001), and RBD Omicron (p-value = 0.004) variants, but not RBD Alpha, RBD Delta, and WT Spike (S). Note, negative MFI values (artifact of the assay and not meaningful outcomes since there cannot be a negative value of antibodies in the samples) were set to 1 to plot the log(10) scale graph and better visualize the full spread of MFIs. Similarly, the few outcomes that ended up being 0 (n=3 study samples, Omicron only) were set to 1 for the same reasons. Vax, Vaccinated. MFI, median fluorescence intensity. RBD, receptor binding domain. Ns, not significant.

SARS-CoV-2 Seroprevalence among Unvaccinated Study Participants

Next, we determined the SARS-CoV-2 seroprevalence among unvaccinated study participants using two different methods. Thus, we compared two different strategies to calculate thresholds and determine which samples are considered positive or negative according to the calculated cut-offs: (1) The first approach was a traditionally applied method where the threshold is set at three standard deviations above the mean of the negative controls (3STDV). (2) The second approach was based on a standardized open-source machine learning-based method that calculates sensitive and specific classification boundaries based on population-specific pre-validated positive and negative controls and gives individual and group-based uncertainty estimates for the seroprevalence outcomes (i.e., SeroNIST) (22–25). We focused on calculating SARS-CoV-2 seroprevalence among the unvaccinated study population

because we aimed to determine the proportion of participants who had been infected by SARS-CoV-2 (i.e., harbour infection-induced antibodies and not vaccine-induced antibodies).

Overall, the SARS-CoV-2 seroprevalence for anti-S and anti-RBD antibodies across all variants, except Omicron, was high (ranging from 89.1% to 100.0%, Table 1) and similar comparing the two methods among the unvaccinated study population. The seroprevalence for anti-RBD antibodies for the Omicron variant resulted in the most significant discrepancy and resulted in 82.2% (+/- 10.2 95% CI) for the SeroNIST method and 10.5% for the 3STDV method.

Table 1. SARS-CoV-2 Seroprevalence among unvaccinated participants (n=247) by threshold assessment method (3STDV vs. SeroNIST).

	3STDV	SeroNIST		
	Seroprevalence %	Seroprevalence % (95% CI)	Sensitivity (95% CI)	Specificity (95% CI)
N WT	66.4	78.9 (+/- 7.7)	100.0 (98.8, 100.0)	72.2 (59.6, 84.2)
S WT	91.9	100.0 (+0/-3.4)	100.0 (98.8, 100.0)	NA
RBD WT	95.1	100.0 (+0/-8.9)	100.0 (98.3, 100.0)	NA
RBD Alpha	89.1	100.0 (+0/-7.0)	100.0 (98.8, 100.0)	NA
RBD Beta	96.0	100.0 (+0/-3.4)	100.0 (98.8, 100.0)	NA
RBD Gamma	97.2	100.0 (+0/-5.6)	100.0 (98.8, 100.0)	NA
RBD Delta	99.6	99.6 (+/- 3.2)	100.0 (98.8, 100.0)	NA
RBD Lambda	98.0	100.0 (+0/-2.8)	100.0 (98.8, 100.0)	NA
RBD Omicron	10.5	82.2 (+/- 10.2)	100.0 (98.8, 100.0)	80.6 (68.2, 91.5)

NA, not applicable because there were too few negative outcomes to evaluate specificity for this analysis.

To determine whether sex, age, education, religion, city of residence, or vaccination status independently increased or decreased the odds of being positive for anti-N WT SARS-CoV-2 antibodies, we ran a multivariable analysis (i.e., logistic regression with binary seropositivity outcomes, see Supplemental Table 8, Supplemental Table 9, and Supplemental Table 13). We focused on the N-based seroprevalence because all the COVID-19 vaccines distributed in Nigeria covered S/RBD but not N antigens. Thus, the presence of anti-N SARS-CoV-2

antibodies were predictive of natural SARS-CoV-2 infection and not vaccination in this setting. Further, the RBD- and S-based seroprevalences were very high (ranged from 82.2% to 100%) and preclude a meaningful analysis (i.e., not enough negative outcomes, see Table 1).

When running the multivariable analysis with age as continuous variable, being from Owo (aOR = 1.5, p-value = 0.045), being female (aOR = 1.4, p-value = 0.049), and being vaccinated (aOR = 0.5, p-value = 0.001) were independently statistically significant and were therefore associated with N WT SARS-CoV-2 serology, see Figure 3 and Supplemental Table 14. Thus, residents from Owo and female study participants had higher odds of being anti-N WT SARS-CoV-2 antibody positive, though those effects were barely statistically significant. Vaccinated individuals were much less likely to be N WT SARS-CoV-2 antibody positive (i.e., of being infected with SARS-CoV-2), and that effect was strong. There was also a strong and association between N WT SARS-CoV-2 seropositivity and not reporting sex (aOR = 7.0, p-value < 0.0001). We reran the analysis including age as categorial variable to confirm these outcomes. Residency (Owo), being female, being vaccinated, and missing a sex descriptor were statistically significant again, see Supplemental Table 15. Thus, in summary, vaccinated individuals had much lower odds of being SARS-CoV-2 positive (likely indicating a protective effect of the vaccine), while there was a mild association between SARS-CoV-2 seropositivity and living in Owo or being female. Age, education, and religion did not influence serological positivity in our analysis. Finally, those who did not report sex were much more likely to be positive for anti-N WT SARS-CoV-2 antibodies, indicating a non-random pattern in missing data.

Figure 3. Adjusted odds ratios for predictors of anti-N SARS-CoV-2 seropositivity. The figure shows adjusted odds ratios (aORs) for anti-N wildtype (WT) SARS-CoV-2 seropositivity in log scale by evaluated predictors (state, age, sex, education, religion, and COVID-19 vaccine uptake). Vaccinated individuals had much lower odds of being positive (p-value = 0.001). Owo residency (p-value = 0.045) and being female (p-value = 0.049) were associated with higher odds of vaccination, though the effects were barely significant. Those who did not

report sex were much more likely to be positive for anti-N WT SARS-CoV-2 antibodies (p-value < 0.0001). Age, education, and religion did not influence serological positivity. Age, education, and religion did not influence serological positivity.

Discussion

The COVID-19 pandemic, caused by SARS-CoV-2, rapidly spread across the globe in 2019/2020, resulting in millions of deaths, overwhelming healthcare systems, disrupting economies, and exposing vulnerabilities in global public health (27,28). Surprisingly, the number of cases and fatalities in Africa was low compared to the rest of the world (29). Nigeria, Africa's most populous country, accounted for less than 1% of the global cases (2). Whether these numbers reflect the true burden of SARS-CoV-2 is yet to be elucidated. The present study evaluated serosurvey-based SARS-CoV-2 exposure, compared serological threshold assessment approaches, determined the risk factors for SARS-CoV-2 infection, and evaluated the COVID-19 vaccine uptake among febrile Southern Nigerian study participants across urban and peri-urban settings in 2022/2023. These study results offer insights into sociodemographic and regional risk factors for SARS-CoV-2 infection, strategies to optimize serological study design, and approaches to improve COVID-19 vaccine uptake in West Africa.

Study Population

We found slightly higher female involvement and most of the participants to be between 25 and 44 years old, which aligns with previous studies showing greater engagement in public health research by women and socially active populations (30,31). This was also reflected in the educational status of the participants, where most people had received post-primary formal education.

Vaccine Uptake

The majority of study participants (66.0%) reported receiving a single or double dose of the COVID-19 vaccine, which is above the previously reported COVID-19 vaccine uptake in

Nigeria, ranging from 20%-58% (32). While prior research indicates that sociodemographic factors significantly influence vaccine uptake (33,34)(35,36), we did not find sex, education, or religion to be strong indicators for COVID-19 vaccine uptake in our study population. However, we found that peri-urban study participants from Owo were more likely to be vaccinated compared to urban counterparts from Ikorodu, indicating differential COVID-19 vaccine uptake across urban gradients in our study area. Since basic socioeconomic factors were not significant determinants of vaccine uptake in our study population, it may have been due to variation in COVID-19 vaccine distribution and accessibility or other factors our study did not capture. Furthermore, we found that older age was strongly associated with the likelihood of being vaccinated, likely reflecting the local vaccine campaign efforts during the study period, which focused on 60+ adults and 50+ adults with co-morbidities(37).

Interestingly, study participants who were anti-N SARS-CoV-2 antibody positive (reflecting natural infection) had much lower odds of being vaccinated. The statistically significant relationship between vaccine uptake and anti-N SARS-CoV-2 antibody positivity was confirmed through subsequent analyses (serology as dependent variable), where vaccinated individuals were much less likely to be N SARS-CoV-2 antibody positive. The effect in both models was strong and may indicate a protective effect of the vaccine, supported by finding higher anti-RBD WT antibodies among vaccinated study participants. The effect could also reflect or be compounded by behavioural modifications where cautious individuals were both more likely to get vaccinated and better at social distancing and hand-washing protocols, therefore preventing SARS-CoV-2 infection during the study period.

SARS-CoV-2 Serosurvey

Vaccinated participants had significantly more anti-RBD antibodies for the WT, Beta, Gamma, Lambda, and Omicron variants (Figure 2). Detecting more anti-S/RBD antibodies in the vaccinated group likely validated the self-reported vaccination status since (i) being vaccinated has been described to elicit a stronger anti-RBD/S antibody response than natural infection,

and (ii) being both vaccinated and infected is likely to induce a higher antibody response than either alone (8,38). However, antibody decay should be taken into account (39,40), and we did not have information on time since vaccination or infection.

SARS-CoV-2 Serosurvey Among Unvaccinated Study Participants

We assessed SARS-CoV-2 seroprevalence among unvaccinated study participants to evaluate SARS-CoV-2 exposure (vs. vaccination). Our study revealed high S/RBD-based seroprevalences for all variants except Omicron among the unvaccinated participants (ranging from 89.1% to 100%), indicating that the level of SARS-CoV-2 exposure was likely underestimated in Nigeria and confirming that reduced viral transmission was not likely the main reason the reported COVID-19 morbidity and mortality were low in Nigeria and the region (6). This is the highest seroprevalence rate for SARS-CoV-2 reported in Nigeria to date (11–16). The variation may reflect differences in sampling period and testing approach, advocating standardized serological methods. While most of the prior serosurveys were implemented between 2020 and mid-2022, our study happened later in the pandemic, between the end of 2022 and beginning of 2023. A similar high seroprevalence of over 90% was reported in Yemen in 2023 (41).

Furthermore, Nigerians may have been exposed to SARS-CoV-2 variants that were not previously reported in the country. Nigeria experienced four major SARS-CoV-2 waves dominated by the ancestral/WT, Alpha, Delta, Eta, and Omicron variants (17–20). Other important variants detected elsewhere, such as Gamma and Lambda variants, were not reported in Nigeria (42), but we detected relatively high levels of anti-RBD antibody for both variants among our study population, although some of the signal may have been due to cross-reactivity. While the affinity between the specific variant antigens (i.e., antigens coupled to the beads in our serological assay) and respective human antibodies should be the highest/tightest, we could not preclude cross-reactivity between SARS-CoV-2 variants and

also with endemic human CoVs (e.g., HKU1, NL63, OC43, and 229E) due to lack of positive controls (i.e., individuals who were exposed only to specific SARS-CoV-2 variants, HKU1, NL63, OC43, or 229E). The low seroprevalence and antibody levels for Omicron RBD detected in our study may have been due to low transmission or due to a distinct serological footprint (43). Further genomic epidemiological approaches and proactive serosurveillance will help unravel the true burden of all SARS-CoV-2 variants in Nigeria.

Risk Factors for SARS-CoV-2 Infection

We evaluated risk factors for SARS-CoV-2 infection through an anti-N WT SARS-CoV-2 serosurvey-based multivariable analysis across all study participants (vaccinated and unvaccinated). Since vaccines distributed in Nigeria during the study period covered S/RBD but not N antigens), we focused on the N-based seroprevalence to estimate risk factors for natural SARS-CoV-2 infection (vs. vaccination). Further, the RBD- and S-based seroprevalences where high and ranged from 82.2% to 100.0%, precluding a meaningful risk factor analysis.

As mentioned above, vaccinated individuals were much less likely to be N WT SARS-CoV-2 antibody positive (p-value = 0.001). Female study participants had higher odds of being positive for anti-N WT SARS-CoV-2 antibodies, though the effect was barely statistically significant (p-value = 0.049). Age, education, and religion did not influence the serological outcome. Thus, we did not find compelling high-risk sociodemographic subpopulations based on age, sex, educational status, or religion, similar to previous studies (11,30). Finally, Owo residents were more likely to be N WT SARS-CoV-2 positive. Though the effect was barely statistically significant (p-value = 0.045), study participants living in peri-urban settings were at higher risk of SARS-CoV-2 infection compared to urban settings (Ikorodu).

Serological Threshold Assessment

Our study compared two threshold calculation methods to evaluate the SARS-CoV-2 seroprevalence. There was concordance between the two approaches, except for anti-RBD Omicron and anti-N WT antibodies. The difference between the N-based seroprevalence was within ~10% when considering the 95%CI, but the Omicron-based seroprevalence differed widely (82.2% vs. 10.5%). The SeroNIST method calculates thresholds by utilizing measurements from positive and negative controls (whereas the 3STDV approach is based on only the negative controls) and the positive controls were cases captured before Omicron circulated in the local population. Hence, they were not good positive controls to determine the SeroNIST-based thresholds for Omicron. Overall, the two methods estimated similar seroprevalence outcomes (except for Omicron, where no appropriate positive controls were available). However, the SeroNIST method represents a more rigorous and standardized approach. It automatically calculates seroprevalence and the associated uncertainty estimates (sensitivity, specificity, and accuracy) for the population-based and individual serological outcomes. Using an open source, standardized, and automated approach across serosurveys (independent of the pathogen of interest) will make the seroprevalence calculations quicker, more informative, and comparable across studies, making the outcomes more suitable for metanalysis and increasing the public health impact of serosurveys overall. Hence, we considered the SeroNIST method superior to the 3SDTV approach.

Limitations

Our study results should be viewed considering a few limitations.

While we were able to enroll 745 study participants, increasing the limited sample size, including additional socioeconomic variables, and being able to access vaccination dates would have given a more nuanced picture of risk factors for SARS-CoV-2 exposure and COVID-19 vaccine uptake. Unfortunately, that information was not available to us. Further, the socio-demographic information and vaccine uptake were self-reported which may lead to recall bias or misrepresentations.

We had missing data for vaccine uptake (n=6, 0.8%), sex (n=100, 13.4%), education (n=54, 7.2%), and age (n=1/275). All the missing data was included as separate categories in the regression analyses, except for age. There were no strong and significant outcomes for the missing data categories except for sex. Those who did not report sex were much more likely to be positive for anti-N SARS-CoV-2 antibodies, indicating a non-random pattern in missing data. Since anti-N SARS-CoV-2 antibody positivity was not a self-reported outcome however, it was not likely to be directly related.

Importantly, our study only covered symptomatic participants who visited local healthcare centers. Hence, it does not represent the general population. Further, individuals who visit health care centers when sick may be more open to engage with the public health system and therefore may be more likely to get vaccinated compared to the general population.

Finally, the main methodological limitation of our study was the inability to account for potential serological cross-reactivity between SARS-CoV-2 variants and potentially other CoVs due to the lack of positive controls for each CoV subtype.

Conclusion and Recommendations

(i) The results of this study suggest that the SARS-CoV-2 exposure in Southern Nigeria was higher than previously reported. Hence, lack of SARS-CoV-2 transmission was not likely the reason that Nigeria, and potentially the region, experienced less COVID-19 disease and related deaths. Still, genomic surveillance is needed to track variance-based exposure patterns (44). (ii) The COVID-19 vaccine uptake was high, with peri-urban participants showing greater coverage than those in urban areas, and increasing age was a strong predictor of vaccination. Hence, the local vaccine uptake strategy implemented prior to 2022/2023 proved effective, resulting in high coverage rates and representing a significant public health success. Interestingly, our study found a strong correlation between being vaccinated and having lower odds of being positive for positive for anti-N WT SARS-CoV-2 antibodies (proxy for SARS-CoV-2 infection). Therefore, the vaccine may have protected study participants from SARS-CoV-2 infection prior to the study. Overall, ongoing public health

interventions should focus on improving vaccine uptake in urban populations and younger age groups while leveraging the observed success of peri-urban vaccine campaigns. (iii) The lack of standardized serological thresholding methods highlights the need for robust analytical approaches. While there was concordance between the two approaches, prioritizing the use of validated, standardized, and automated thresholding methods such as SeroNIST will enable reliable comparisons of serosurveys across studies and time, thereby increasing the public health impact of serosurveys.

Acknowledgements

We thank the study participants and their families for their valuable time and participation. Without them, this study would not have been possible.

Financial Support

Funding was provided by (1) the Centers for Disease Control and Prevention (CDC) through the U01GH002338-01 grant (RRD, CTH, and AMM) titled "Seroepidemiological Insight into COVID-19 Transmission in Africa (SICA)", (2) the National Institute of Health (NIH) through the National Center for Advancing Translational Sciences KL2-TR001455 grant (RAB), and (3) the Bill and Melinda Gates Foundation through the GIISER Project ARISE-INV-036304 (CTH and CAU). The funders had no role in study design, data collection and analysis, decision to publish, or preparation of the manuscript.

Conflicts of Interest

The authors have declared that no competing interests exist.

References

1. Karcıoğlu O, Yüksel A, Baha A, Er AB, Esendağlı D, Gülhan PY, et al. Covid-19: The Biggest Threat of the 21st Century: In Respectful Memory of the Warriors All Over the World. Turk Thorac J. 2020 Dec 11;21(6):409–18.
2. WHO. COVID-19 Epidemiological Update. WHO; 2024.
3. Cabore JW, Karamagi HC, Kipruto HK, Mungatu JK, Asamani JA, Droti B, et al. COVID-19 in the 47 countries of the WHO African region: a modelling analysis of past trends and future patterns. Lancet Glob Health. 2022 Aug;10(8):e1099–114.
4. Maeda JM, Nkengasong JN. The puzzle of the COVID-19 pandemic in Africa. Science. 2021 Jan;371(6524):27–8.
5. Happi CT, Nkengasong JN. Two years of COVID-19 in Africa: lessons for the world.
6. Bejon P, Agweyu A, Oyier I, Hamaluba M, Kamuya D, Kinyanjui S, et al. Rethinking the Evidence on COVID in Africa. Lancet Infect Dis [Internet]. 2025 Apr 25 [cited 2025 June 21]; Available from: https://doi.org/10.1016/ S1473-3099(25)00071-4
7. Adams J, MacKenzie MJ, Amegah AK, Ezeh A, Gadanya MA, Omigbodun A, et al. The Conundrum of Low COVID-19 Mortality Burden in sub-Saharan Africa: Myth or Reality? Glob Health Sci Pract. 2021 Sept 30;9(3):433–43.
8. Ugwu CA, Alao O, John OG, Akinnawo B, Ajayi I, Odebode O, et al. Immunological insights into COVID-19 in Southern Nigeria. Front Immunol. 2024 Jan 23;15:1305586.
9. Ojo TO, Ojo AO, Ojo OE, Akinwalere BO, Akinwumi AF. Determinants of COVID-19 vaccine uptake among Nigerians: evidence from a cross-sectional national survey. Arch Public Health. 2023 May 26;81(1):95.

10. Ojeniyi T, Eguavoen A, Chinye-Nwoko F. Moving the needle for COVID-19 vaccinations in Nigeria through leadership, accountability, and transparency. Front Public Health. 2023 Sept 21;11:1199481.

11. Audu RA, Stafford KA, Steinhardt L, Musa ZA, Iriemenam N, Ilori E, et al. Seroprevalence of SARS-CoV-2 in four states of Nigeria in October 2020: A population-based household survey. Nelson MI, editor. PLOS Glob Public Health. 2022 June 17;2(6):e0000363.

12. Chechet GD, Kwaga JKP, Yahaya J, Noyes H, MacLeod A, Adamson WE. SARS-CoV-2 seroprevalence at urban and rural sites in Kaduna State, Nigeria, during October/November 2021, immediately prior to detection of the Omicron variant. Int J Epidemiol. 2022 Oct 13;51(5):1361–70.

13. Ige FA, Ohihoin GA, Osuolale K, Dada A, Onyia N, Johnson A, et al. Seroprevalence of SARS-CoV-2 IgG among healthcare workers in Lagos, Nigeria. Olu OO, editor. PLOS ONE. 2023 Oct 5;18(10):e0292440.

14. Kolawole OM, Tomori O, Agbonlahor D, Ekanem E, Bakare R, Abdulsalam N, et al. SARS CoV-2 Seroprevalence in Selected States of High and Low Disease Burden in Nigeria. JAMA Netw Open. 2022 Oct 11;5(10):e2236053.

15. Olayanju O, Bamidele O, Edem F, Eseile B, Amoo A, Nwaokenye J, et al. SARS-CoV-2 Seropositivity in Asymptomatic Frontline Health Workers in Ibadan, Nigeria. Am J Trop Med Hyg. 2021 Jan 6;104(1):91–4.

16. Akanmu S, Herrera BB, Chaplin B, Ogunsola S, Osibogun A, Onawoga F, et al. High SARS-CoV-2 seroprevalence in Lagos, Nigeria with robust antibody and cellular immune responses. J Clin Virol Plus. 2023 Aug;3(3):100156.

17. Olawoye IB, Oluniyi PE, Oguzie JU, Uwanibe JN, Kayode TA, Olumade TJ, et al. Emergence and spread of two SARS-CoV-2 variants of interest in Nigeria. Nat Commun. 2023 Feb 13;14(1):811.

18. Olorunfemi AB, Suliman SAR, Tran TT, Ayorinde B, Fowora MA, Iwalokun BA, et al. Whole genome sequencing and phylogenetic analysis of SARS-CoV-2 strains isolated during the COVID-19 pandemic in Nigeria. IJID Reg. 2024 Mar;10:174–8.

19. Kolawole DB, Okeke MI. Phylogenetic and genome-wide mutational analysis of SARS-CoV-2 strains circulating in Nigeria: no implications for attenuated COVID-19 outcomes. Osong Public Health Res Perspect. 2022 Apr 30;13(2):101–13.

20. Akande OW, Elimian KO, Igumbor E, Dunkwu L, Kaduru C, Olopha OO, et al. Epidemiological comparison of the first and second waves of the COVID-19 pandemic in Nigeria, February 2020–April 2021. BMJ Glob Health. 2021 Nov;6(11):e007076.

21. McConnell D, Hickey C, Bargary N, Trela-Larsen L, Walsh C, Barry M, et al. Understanding the Challenges and Uncertainties of Seroprevalence Studies for SARS-CoV-2. Int J Environ Res Public Health. 2021 Apr 27;18(9):4640.

22. Binder RA, Fujimor GF, Forconi CS, Reed GW, Silva LS, Lakshm P, et al. SARS-CoV-2 Serosurveys: How Antigen, Isotype and Threshold Choices Affect the Outcome. J Infect Dis. 2022 Oct 31;2023(227):371–80.

23. Binder RA, Matta AM, Forconi CS, Oduor CI, Bedekar P, Patrone PN, et al. Minding the margins: Evaluating the impact of COVID-19 among Latinx and Black communities with optimal qualitative serological assessment tools. Egorov AI, editor. PLOS ONE. 2024 July 25;19(7):e0307568.

24. Patrone PN, Binder RA, Forconi CS, Moormann AM, Kearsley AJ. Analysis of Diagnostics (Part I): Prevalence, Uncertainty Quantification, and Machine Learning

[Internet]. arXiv; 2024 [cited 2025 June 21]. Available from: http://arxiv.org/abs/2309.00645

25. Patrone PN, Binder RA, Forconi CS, Moormann AM, Kearsley AJ. Analysis of Diagnostics (Part II): Prevalence, Linear Independence, and Unsupervised Learning [Internet]. arXiv; 2024 [cited 2025 June 21]. Available from: http://arxiv.org/abs/2408.16035

26. Soetewey A. Binary logistic regression in R. Stats and R [Internet]. Stats and R. 2024. Available from: https://statsandr.com/blog/binary-logistic-regression-in-r/

27. Faramarzi A, Norouzi S, Dehdarirad H, Aghlmand S, Yusefzadeh H, Javan-Noughabi J. The global economic burden of COVID-19 disease: a comprehensive systematic review and meta-analysis. Syst Rev. 2024 Feb 16;13(1):68.

28. Nicola M, Alsafi Z, Sohrabi C, Kerwan A, Al-Jabir A, Iosifidis C, et al. The socio-economic implications of the coronavirus pandemic (COVID-19): A review. Int J Surg. 2020 June;78:185–93.

29. Joseph S, Kutty Narayanan A. Covid 19-the 21st Century Pandemic: The Novel Coronavirus Outbreak and the Treatment Strategies. Adv Pharm Bull. 2021 Jan 30;1.

30. Onifade AA, Fowotade A, Rahamon SK, Edem VF, Yaqub SA, Akande OK, et al. Seroprevalence of anti-SARS-CoV-2 specific antibodies in vaccinated and vaccine naïve adult Nigerians. Bhattacharya D, editor. PLOS ONE. 2023 Jan 23;18(1):e0280276.

31. Wu C, Qian Y. The gender peak effect: Women are most vulnerable to infections during COVID-19 peaks. Front Public Health. 2022 Aug 9;10:937179.

32. Olu-Abiodun O, Abiodun O, Okafor N. COVID-19 vaccination in Nigeria: A rapid review of vaccine acceptance rate and the associated factors. Elelu N, editor. PLOS ONE. 2022 May 11;17(5):e0267691.

33. Tchiasso D, Mendjime P, Njuwa Fai K, Nana Wandji BS, Yuya F, Youm É, et al. Dynamic factors associated with COVID-19 vaccine uptake in Cameroon between 2021 and 2022. J Public Health Afr [Internet]. 2024 Oct 24 [cited 2024 Dec 8];15(1). Available from: https://publichealthinafrica.org/index.php/jphia/article/view/578

34. White TM, Wyka K, Rabin K, El-Mohandes A. Trust in the science behind COVID-19 vaccines as a driver of vaccine acceptance in the United States, 2021–2023. Vaccine X. 2024 Dec;21:100576.

35. Costa JC, Weber AM, Darmstadt GL, Abdalla S, Victora CG. Religious affiliation and immunization coverage in 15 countries in Sub-Saharan Africa. Vaccine. 2020 Jan;38(5):1160–9.

36. Santos TM, Cata-Preta BO, Wendt A, Arroyave L, Hogan DR, Mengistu T, et al. Religious affiliation as a driver of immunization coverage: Analyses of zero-dose vaccine prevalence in 66 low- and middle-income countries. Front Public Health. 2022 Oct 25;10:977512.

37. Nomhwange T, Wariri O, Nkereuwem E, Olanrewaju S, Nwosu N, Adamu U, et al. COVID-19 vaccine hesitancy amongst healthcare workers: An assessment of its magnitude and determinants during the initial phase of national vaccine deployment in Nigeria. eClinicalMedicine. 2022 Aug;50:101499.

38. Kaplonek P, Deng Y, Shih-Lu Lee J, Zar HJ, Zavadska D, Johnson M, et al. Hybrid immunity expands the functional humoral footprint of both mRNA and vector-based SARS-CoV-2 vaccines. Cell Rep Med. 2023 May;4(5):101048.

39. Van Elslande J, Oyaert M, Lorent N, Vande Weygaerde Y, Van Pottelbergh G, Godderis L, et al. Lower persistence of anti-nucleocapsid compared to anti-spike antibodies up to one year after SARS-CoV-2 infection. Diagn Microbiol Infect Dis. 2022 May;103(1):115659.

40. Gallais F, Gantner P, Bruel T, Velay A, Planas D, Wendling MJ, et al. Evolution of antibody responses up to 13 months after SARS-CoV-2 infection and risk of reinfection. eBioMedicine. 2021 Sept;71:103561.

41. Taher WT, Bawazir AA, Sallam TA, Alsurimi K. Seroprevalence and factors associated with SARS-CoV-2 infection among healthcare workers: cross-sectional study. BMC Infect Dis. 2023 Nov 6;23(1):761.

42. Gozzi N, Chinazzi M, Davis JT, Mu K, Pastore Y Piontti A, Ajelli M, et al. Real-time estimates of the emergence and dynamics of SARS-CoV-2 variants of concern: A modeling approach. Epidemics. 2024 Dec;49:100805.

43. Qu P, Faraone JN, Evans JP, Zheng YM, Carlin C, Anghelina M, et al. Enhanced evasion of neutralizing antibody response by Omicron XBB.1.5, CH.1.1, and CA.3.1 variants. Cell Rep. 2023 May;42(5):112443.

44. Happi CT, Nkengasong JN. Two years of COVID-19 in Africa: lessons for the world.

Supplemental Figure 1. Controls and 3STDV Threshold. The graph shows raw median fluorescence intensity (MFI) outcomes of the positive controls (red dots) and the negative controls (blue dots) by SARS-CoV-2 variant. In green are the 3STDV thresholds (based on negative controls only), with respective values on top. Note, negative MFI values (artifact of the assay and not meaningful outcomes since there cannot be a negative value of antibodies in the samples) were set to 1 to plot the log(10) scale graph and better visualize the full spread of MFIs. MFI, median fluorescence intensity. S, Spike. RBD, receptor binding domain. N, Nucleoprotein.

Supplemental Figure 2. SeroNIST Classification Boundary based on three Antibodies. (A) This figure shows the classification boundary (i.e., three-dimensional threshold in rainbow

colours) created by the SeroNIST fool for wildtype (WT) RBD, S, and N antibodies based on the training data (i.e., negative control in blue dots and positive controls in red dots). (B) This figure shows where the data points (blue dots) fall for an example data set of participants. In this case, all the data points fall above the classification boundary, resulting in an estimated SARS-CoV-2 seroprevalence of ~100% based on RBD, S, and N antibodies.

Supplemental Table 1. Residency of study participants (n=745).

Supplemental Table 2. Sex distribution among study participants (n=745).

Supplemental Table 3. Age of study participants (by category, n=745).

Supplemental Table 4. Educational level of study participants (n=745).

Supplemental Table 5. Religion among study participants (n=745).

Supplemental Table 6. Vaccine uptake among study participants (n=745).

Supplemental Table 7. Vaccine uptake by region/state.

Supplemental Table 8. Education variable for multivariable analysis. No formal education (n=8) and primay education (n=130) were combined (n=138).

Supplemental Table 9. Religion variable for multivariable analysis. No religion (n=1) and Other (n=36) were combined (n=37).

Supplemental Table 10. Model outputs for vaccine uptake multivariable analysis (age as continuous variable).

Supplemental Table 11. Categorical age variable for vaccine uptake multivariable analysis.

Supplemental Table 12. Model outputs for vaccine uptake multivariable analysis (age as categorical variable).

Supplemental Table 13. N WT SARS-CoV-2 seropositivity among study participants.

Supplemental Table 14. Model outputs for N seropositivity multivariable analysis (age as continuous variable).

Supplemental Table 15. Model outputs for N seropositivity multivariable analysis (age as categorical variable).

Click here to access/download;Figure;Figure3.tif

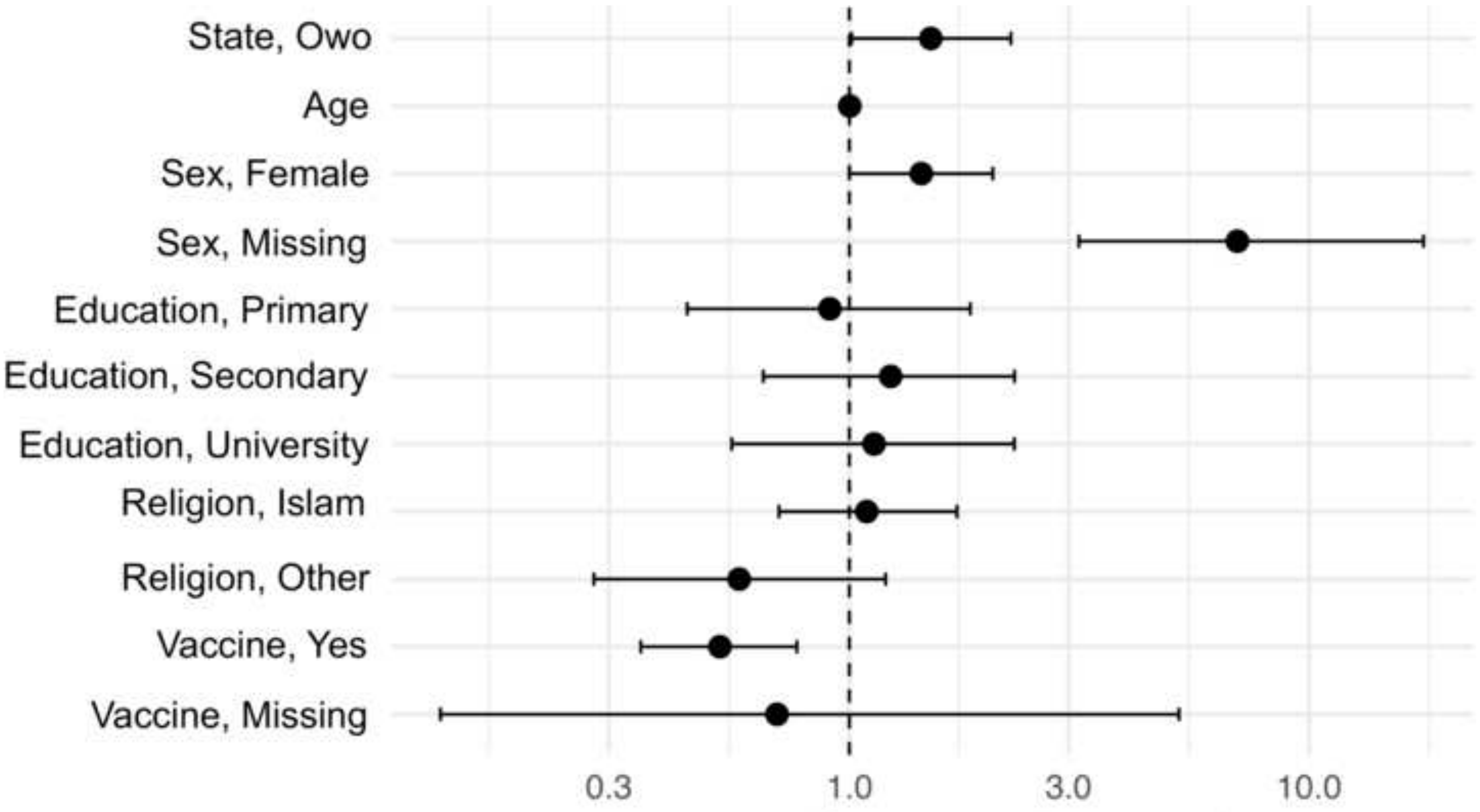

Figure 2

Click here to access/download;Figure;Figure2.tif

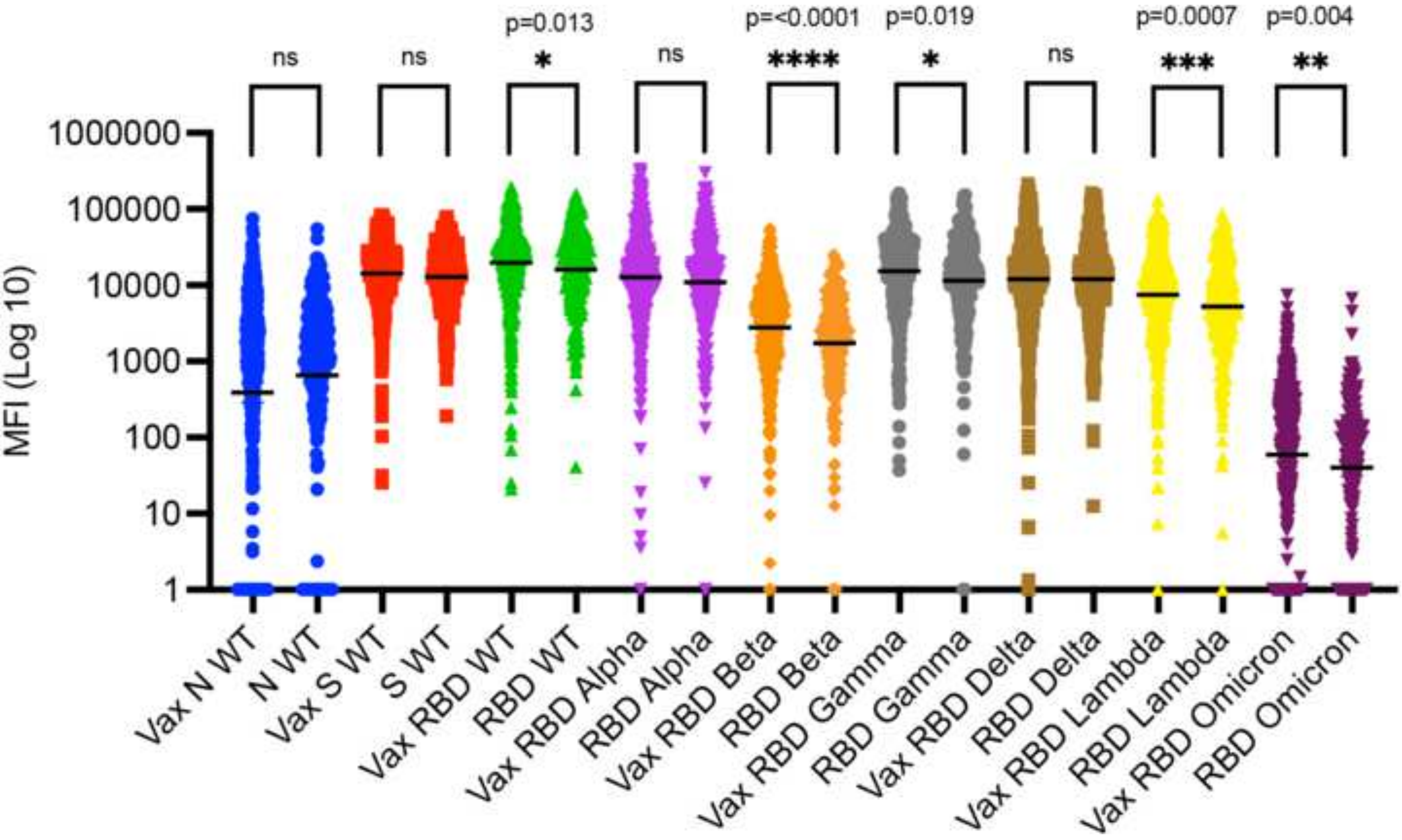

Figure 1

Click here to access/download;Figure;Figure1.tif

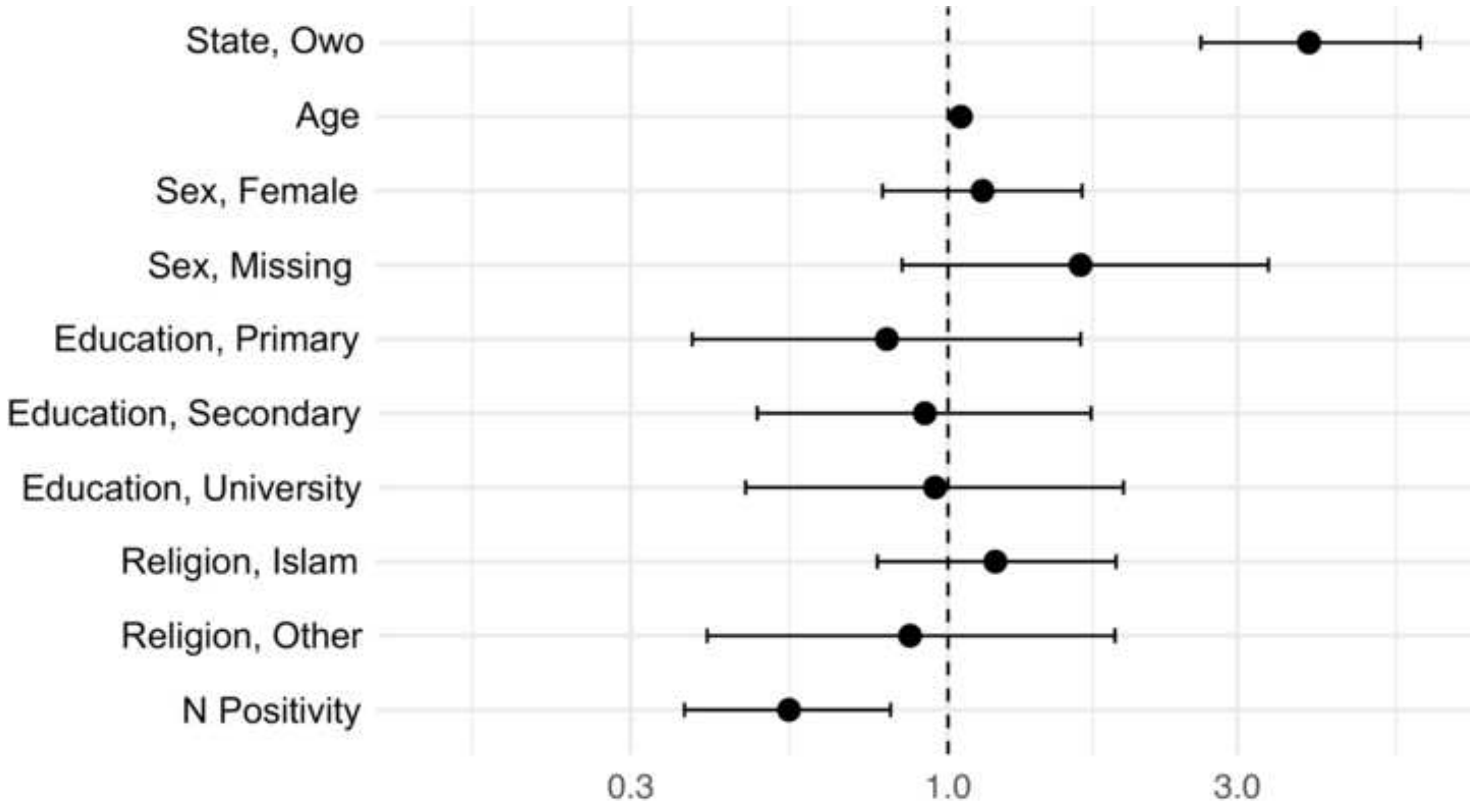

Click here to access/download
Supporting Information
Supplemental Tables_Final.xlsx

Click here to access/download
Supporting Information
Inclusivity questionnaire_PGPH-D-25-02354.docx

Revised Manuscript with Track Changes

COVID19 Sero-epidemiology and Vaccine Uptake in Two Southern Nigerian Clinics

Chinedu A. Ugwu[1,2], Ooreofe Odebode[1,2], Israel O. Ajayi[1,2], Josiah Olubunmim[1,2], Bejide Ifeoluwa[1,2], Jolly A. Adole[1,2], Kazeem Akano[1,2], Johnson Okolie[1], Philomena Eromon[1], Imonikhe K. Kio[1], Judith Amadi[1], Precious F. Adebayo[1], Iheanyi Mgbeokwere[1], Akeem O. Lawal[3], Olayemisi A. Adegoke[3], Amoke O. Adeyemi[1], Sampson Owhin[4], Oladele. O. Ayodeji[4], Alebiosu Ahmed[1], Etim Henshaw[1], Oyejide E. Nicholas[1], Abah A. Sylvester[1,4], Abiola Omidele[1,4], Kolade Emilola[1], Olàyiwolà Funmilayo[1], Adeboye Rufus Oyewale[1], Oluwatuyi O. Olakunle[1], Obaado Oluwaseun[1], Angela M. Matta[5], Prajakta Bedekar[6,7], Latifeh Dahmash[8], Catherine S. Forconi[5], Ann M. Moormann[5], Rhoel R. Dinglasan[8], Christian T. Happi[1,2], Raquel A. Binder[5*]

Affiliations

[1]The Africa Centre of Excellence for Genomics of Infectious Diseases (ACEGID), Redeemer's University Nigeria, Ede, Nigeria.

[2]Department of Biological Sciences, Faculty of Natural Sciences, Redeemer's University, Ede, Osun State Nigeria.

[3]Ikorodu General Hospital Ikorodu, Lagos State Nigeria

[4]Federal Medical Centre Owo, Ondo State Nigeria

[5]Department of Medicine, University of Massachusetts Chan Medical School, Worcester, MA, USA

[6]Department of Applied Mathematics and Statistics, Johns Hopkins University, Baltimore, MD, USA

[7]Department of Mathematics, BITS Pilani, Hyderabad, Telangana, India

[8]Department of Infectious Diseases & Immunology, College of Veterinary Medicine, University of Florida, Gainesville, FL, USA

***Corresponding author**: raquel.binder@umassmed.edu

Running title: COVID-19 and Vaccine Uptake in Southern Nigeria

Abstract word count: 2698/300

Main text word count: 4,7425,321

Abstract

Background: Africa has reported lower COVID-19 morbidity and mortality compared to other regions. Yet, the flat COVID-19 trajectory remains to be fully explained. Nigeria, Africa's most populous country, accounted for less than 1% of global COVID-19 cases. The present study evaluated SARS-CoV-2 seroprevalence, vaccine uptake, and methodological differences in serological threshold setting among febrile patients attending peri-urban and urban clinics in Southern Nigeria. Methods: Between October 2022 and February 2023, 745 febrile participants were recruited from Owo (Ondo State) and Ikorodu (Lagos State). Serum samples were tested for IgG antibodies against wild-type and variant SARS-CoV-2 antigens using a multiplex bead-based antibody assay. Seroprevalence was calculated using two classification boundary methods: (i) the conventional three standard deviations above the mean of negative controls (3STDV) and (ii) the open-source SeroNIST tool. Vaccine uptake and risk factors for exposure were assessed. Results: Results: Vaccine uptake was 66.0% and significantly higher in peri-urban Owo (80.2%) than urban Ikorodu (52.6%, p-value < 0.0001). Increasing age was positively associated with vaccination (p-value < 0.0001). Sex, education, and religion did not influence vaccine uptake. Vaccinated individuals had significantly lower odds of being positive for anti-N SARS-CoV-2 antibodies (p-value = 0.001), indicating a potential protective effect of the vaccine. Age, education, and religion did not influence anti-N SARS-CoV-2 seropositivity. Overall, our study revealed high S/RBD-based seroprevalences for all variants except Omicron among the unvaccinated participants (range: 89.1% - 100%). Vaccine uptake was 66.6% and significantly higher in peri-urban Owo (80.2%) than urban Ikorodu (52.6%, p < 0.001). Increasing age was positively associated with vaccination (p < 0.001). Among unvaccinated participants, the anti-RBD/S SARS-CoV-2 seroprevalence was remarkably high

Formatted: Pattern: Clear (Background 1)

Formatted: Pattern: Clear (Background 1), Highlight

Formatted: Pattern: Clear (Background 1)

Formatted: Underline

Formatted: Pattern: Clear (Background 1)

~~and ranged from 89.1% to 100%. No significant differences in seroprevalence were observed across sex, education, or religion. Anti-N seroprevalence was similar among the vaccinated and unvaccinated groups, suggesting that both groups were exposed equally to SARS-CoV-2 prior to our study period.~~ ~~Conclusions:~~ Conclusions: SARS-CoV-2 exposure in Southern Nigeria was likely widespread and underrecognized, with high seroprevalence among symptomatic peri-urban and urban populations. Peri-urban communities demonstrated higher vaccine uptake than urban counterparts, and age-targeted campaigns likely drove uptake among older individuals. Further, our study detected a potential protective effect of the vaccine among the study population. Finally, s~~S~~tandardized thresholding methods such as SeroNIST are critical for accurate seroprevalence estimates.

Key words: COVID-19, Nigeria, Serosurvey, Vaccine Uptake, Threshold method

Introduction

Severe acute respiratory syndrome coronavirus 2 (SARS-CoV-2), causing coronavirus disease 2019 (COVID-19), was first identified in Wuhan, China, in December of 2019 and has since rapidly disseminated across the globe, causing the biggest pandemic of the 21st century (1). Close to 800 million cases and around 7 million deaths have been attributed to COVID-19, which is likely underestimating the true global burden (2). Surprisingly, Africa experienced a less catastrophic outbreak compared to the rest of the world, accounting for less than 2% of the global cases (3). Nigeria, the most populated country in Africa, accounted for just 0.003% of the global cases (2). At first, limited testing, a younger population, reduced transmission, preexisting immunity, genetic protective factors, early implementation of public health measures, and timely leadership were proposed to drive the flat COVID-19 trajectory (4,5). More recent evidence suggests that reduced viral transmission and poor surveillance were not the main reasons the reported COVID-19 morbidity and mortality were low (6). Instead, the population age structure and a reduced anti-SARS-CoV-2 inflammatory response due to specific immunoregulation or cross-reactive pre-pandemic immunity are likely the main

Formatted: Pattern: Clear (Background 1), Highlight

Formatted: Pattern: Clear (Background 1)

Formatted: Underline

Formatted: Pattern: Clear (Background 1)

protective factors (6). Similar factors were likely the reason a low number of COVID-19 cases and related deaths were reported in Nigeria (7,8). ~~To better understand the extent of existing~~ To better understand the extent of existing immunity to SARS-CoV-2 in Nigeria and risk factors of infection, we implemented a study measuring seroprevalence against different variants of SARS-CoV-2 between October 2022 and February 2023 in two Southern Nigerian clinics covering peri-urban and urban settings. ~~Given the varying vaccine availability and acceptability in the region,~~ Given the varying vaccine availability and acceptability in the region, we also evaluated the COVID-19 vaccine uptake. At the time of the study, ~~seven~~ the following vaccines were approved for emergency use in Nigeria and available in our study population: Vaxveria (Oxford/ AstraZeneca), Covishield (Serum Institute of India), Comirnaty (Pfizer/BioNTech), Jcovden (Johnson and Johnson), Spikevax (Moderna), Sputnik V (Gamaleya). Note, Covilo (Sinopharm) was approved but not used in our study population. ~~, and Covilo (Sinopharm).~~ By 2023, ~110 million doses of COVID-19 vaccines had been distributed and ~30% of the population had full vaccine coverage (9,10).

~~Sero-epidemiological studies (serosurveys)~~ Sero-epidemiological studies (serosurveys) evaluate pathogen-specific antibodies, thus serving as a tool to evaluate the burden of infection and existing immunity among populations. Serosurveys are particularly valuable in contexts where clinical reporting and molecular testing for infectious diseases are constrained (11). There are limited SARS-CoV-2 serosurveys implemented in Nigeria, and most of them focused on health care workers or only assessed the ancestral SARS-CoV-2 strain (i.e., wildtype [WT] or Wuhan) (11–16). Between February 2020 and November 2022, Nigeria had experienced four major COVID-19 waves, which were dominated by the wildtype (WT)/ancestral, Alpha, Delta, Eta, and Omicron variants (17–20). Other important variants detected elsewhere, such as Gamma and Lambda, were not reported in Nigeria. Whether this means these variants did not circulate in Nigeria or whether they went unreported is unclear.

Formatted: Pattern: Clear (Background 1)

Formatted: Pattern: Clear (Background 1)

Formatted: Highlight

Formatted: Pattern: Clear (Background 1)

Formatted: Pattern: Clear (Background 1)

Formatted: Pattern: Clear (Background 1), Highlight

Formatted: Pattern: Clear (Background 1)

Formatted: Highlight

Formatted: Highlight

While serosurveys are essential public health tools, the applied downstream analysis methods to convert quantitative outcomes (e.g., median fluorescence Intensity [MFI] values from bead-based assays or optical density [OD] values from enzyme-linked immunosorbent assays [ELISAs]) into qualitative results (positive/negative) vary greatly, making metanalyses and comparisons of outcomes across studies and regions challenging (21,22). Here, we applied a validated weighted-standard curve across-plate normalization method (23) and compared two classification boundary methods for optimal qualitative serological assessments: (~~for optimal qualitative serological assessments. (~~1) The traditionally applied three standard deviations above the mean of the negative controls (3STDV), and (2) an open-source machine learning-based tool that calculates sensitive and specific classification boundaries based on population-specific positive and negative controls, which has been termed SeroNIST (22–25).

In summary, we implemented a 2022/2023 SARS-CoV-2 serosurvey in Southern Nigeria based on standardized serological tools, determined the risk factors for infection, and evaluated the COVID-19 vaccine uptake among symptomatic individuals visiting local health care centers. Understanding the sero-epidemiological dynamics of the disease and contrasting peri-urban and urban settings is crucial for assessing the population's exposure, immunity levels, and potential risk factors. By examining the SARS-CoV-2 seroprevalence in Southern Nigeria, this study bridged knowledge gaps about regional risk factors for infection and population-level exposures. Finally, assessing COVID-19 vaccine uptake allowed us to evaluate ~~the potential impact of vaccine-associated reduced disease that can~~coverage and inform future public health strategies aiming to increase vaccine ~~coverage~~uptake.

Methods

Study Clinics

The study was conducted at two clinical sites in Southern Nigeria: (1) The Federal Medical Centre, Owo (FMC Owo), in Ondo State from October 19 to November 22, 2022, and (2) the

Formatted: Indent: First line: 0"

Formatted: Highlight

Formatted: Indent: First line: 0"

Formatted: Font: Bold

General Hospital, Ikorodu (Ikorodu GH) in Lagos State from November 14, 2022, to February 15, 2023. These sites were selected based on their capacity to support surveillance research among febrile populations and their existing clinical diagnostic and research infrastructure. Both facilities serve large catchment populations and represent diverse epidemiological settings, covering both peri-urban (Owo) and urban (Ikorodu) sites and therefore offering a unique opportunity to assess SARS-CoV-2 exposure and COVID-19 vaccine uptake. Additional information regarding the ethical, cultural, and scientific considerations specific to inclusivity in global research is included in the Supporting Information, see Supplemental Checklist.

FMC Owo is a federal tertiary healthcare institution with peri-urban extension services and a fully functional laboratory run by the Africa Centre of Excellence for Genomics of Infectious Diseases (ACEGID). The facility supports a wide range of diagnostic capabilities and biological sample processing. Ikorodu GH is a high-volume secondary health facility serving an urban population just outside metropolitan Lagos, with clinical services and infrastructure well-suited to the project's needs.

Formatted: Indent: First line: 0"

Both sites provided centralized coordination of participant enrolment, data collection, and sample processing. Field teams were trained in Good Clinical Practice (GCP), biosafety procedures, and the use of standardized data collection tools. Community engagements were carried out at both sites to ensure culturally appropriate and voluntary participation. The study design ensured contextual relevance, operational feasibility, and scientific rigor for characterizing the SARS-CoV-2 epidemiology and COVID-19 vaccine uptake in distinct healthcare settings within southern Nigeria.

Formatted: Indent: First line: 0"

Human Subjects

Formatted: Font: Bold

The study protocol, informed consent procedures, and all associated study tools were reviewed and approved by the Nigerian National Health Research Ethics Committee (NHREC)

and the Health Research Ethics Committees (HRECs) of Owo FMC (NHREC/01/01/2007-31/08/2021). Similarly, the University of Florida Institutional Review Board (IRB) reviewed and approved the study and associated documents (IRB202102308). Ethical oversight covered all aspects of study implementation, including participant recruitment, data collection, and biological sampling.

Inclusion criteria: febrile individuals across all ages seeking care at either study clinic. All participants provided written informed consent prior to enrolment. For participants under the age of 18, assent was obtained in conjunction with parental or guardian consent. Trained field personnel conducted the consent process using IRB-approved forms. The consent process clearly outlined the study objectives, procedures, potential risks, and the voluntary nature of participation. Participants were informed of their right to withdraw from the study at any time without penalty. Confidentiality was maintained throughout the study. Each participant was assigned a unique identifier, which was used to label all data and specimens. Electronic data were entered into encrypted REDCap databases hosted on password-protected devices. To mitigate risks, field staff were trained in infection prevention and control practices and used personal protective equipment during all participant interactions. These measures ensured ethical standards were upheld while maximizing participant safety and study integrity.

Sample and Data Collection

Participant recruitment occurred through the outpatient departments of both hospitals. Individuals presenting with acute febrile illness were screened for eligibility (i.e., consenting abilities) and invited to participate. Post-consent, a brief survey (capturing basic demographics and COVID-19 vaccine uptake) and a blood sample were collected. Each participant provided finger-prick whole blood through an EDTA microtainer tube. Serum was separated onsite using portable centrifuges and stored -20 °C under cold-chain conditions. Each sample was labelled with a unique study identifier and linked to participant data through encrypted REDCap databases. Stored and processed samples were transported from the site using a dry shipper

Formatted: Indent: First line: 0"

Formatted: Font: Bold

(pre-charged to -20 °C) to the ACEGID laboratory at Redeemer's University for bead-based multiplex serology (Luminex MagPix). ~~Serum was separated onsite using portable centrifuges and stored under cold conditions. Each sample was labelled with a unique study identifier and linked to participant data through encrypted REDCap databases. Processed samples were transported to the ACEGID laboratory at Redeemer's University for bead-based multiplex serology (Luminex MagPix).~~

***Serology* - Multiplex Bead-based Antibody Assay**

A bead-based IgG SARS-CoV-2 multiplex assay was implemented as previously described (23). Briefly, wild-type (WT; Wuhan) full-length spike (S), WT nucleocapsid (N), WT receptor-binding domain (RBD), RBD Alpha, RBD Beta, RBD Gamma, RBD Delta, RBD Lambda, and RBD Omicron SARS-CoV-2 antigens were coupled to MagPlex microspheres (Luminex Corporation). Bovine Serum Albumin (BSA) coupled beads were used to control for bead-specific background noise. All coupled beads were diluted in ABE buffer (PBS, BSA 0.1%, TWEEN 20%, Sodium Azide 0.05%) to make a master mix of all the coupled antigens (consistent concentration across all beads, 1,000,000 beads/mL). Then, the master mix was added to each well of a 96-well plate and placed on a magnet to allow the beads to settle and remove the ABE buffer. Subsequently, 50 µL of human serum samples diluted (1:100) in ABE buffer were added to the wells, followed by a 2-hour incubation period on a plate shaker (300 rpm). Next, the beads were washed three times with 125 µL ABE buffer while plate remained on the magnet. Then 50 µL of biotinylated anti-human secondary IgG (BD Pharmingen) diluted in ABE (1:1000) was added to each well and followed by a one-hour incubation at room temperature on a plate shaker (300 rpm). The beads were once again washed three times, and 50 µL of phycoerythrin-conjugated streptavidin (1:1000; BD Pharmingen catalog # 554061) diluted in ABE added to the wells. Next, the beads were set on the plate shaker at 300 rpm for 15 minutes. After the final incubation period, the beads were washed 3 times for the last time and resuspended in 125 µL of ABE. After resuspension, the plate was read on the Luminex MagPix instrument, where a median fluorescence intensity (MFI) value was

Formatted: Font: Bold

generated for each sample and individual antigen with the corresponding bead count. Once the control MFIs (positive and negative controls), standards (dilution of positive control), and bead counts (minimum 50 beads per antigen in each well) were validated, BSA was subtracted (including for the standards) to account for non-specific binding. Next, we applied an across-plate normalization factor based on the standards and transformed the MFIs into qualitative (positive and negative outcomes) as previously described (22–25). Previously collected de-identified banked blood/serum samples served as negative (n=36) and positive (n=21) controls (8).

Statistics

Formatted: Font: Bold

Statistical calculations and graphs were completed in R Studio (version 2024.09.0+375), GraphPad Prism (version 10.5.0), and the open-source SeroNIST tool (available at following link: https://github.com/SeroNISTPI/SeroNIST).

Frequency and percentages were used to describe the sociodemographic descriptors and vaccine uptake among the study participants. ~~(Table 1).~~ The Pearson's Chi-squared test with Yates' continuity correction was used to assess differences in vaccine up-take based on sociodemographic variables and by region, while excluding missing data. ~~(assuming it was missing at random). To assess the association between age (as continuous variable) and vaccine uptake a logistic regression was applied, while excluding missing data (same, assuming it was missing at random). The resulting estimate for age was used to calculate Odds Ratios.~~The multivariable analysis/logistic regression for COVID-19 vaccine uptake and serology (positive or negative for anti-N SARS-CoV-2 antibodies) were run in R Studio with the glm function (26)~~(1)~~. For the vaccine uptake analysis, the missing data points for vaccine uptake (n=6, 0.8%) were excluded since a binary logistic regression requires an outcome/dependent variable with binary outcomes. For the serological analysis, the missing data points for vaccine uptake (n=6, 0.8%) were included. The following variables also had missing data: sex (n=100, 13.4%) and education (n=54, 7.2%), which were included as

separate categories in all the regression analyses. Age had a single missing data point (n=1), which was not included in any of the models due to the small sample size. The resulting estimates were used to calculate adjusted Odds Ratios (aOR) and 95% confidence intervals (95% CI) which are all listed in the Supplemental Tables.

To visualize anti-SARS-CoV-2 WT and variant antibodies among vaccinated and unvaccinated study participants, raw median fluorescence intensity (MFI) and respective geometric mean and 95% CI were plotted. ~~(Figure 1 and Figure 2).~~ The geometric mean (vs. standard average) was applied to minimize the effect of outliers. Differences between vaccinated and unvaccinated groups for each SARS-CoV-2 WT and variants were assessed with the unpaired nonparametric Mann–Whitney U test (a.k.a. Wilcoxon rank-sum test~~; Figure 1~~). Note, while the graph in Figure ~~1~~ 2 is in Log(10) scale to visualize the MFI spread, the statistical test was applied to raw/non-transformed numbers.

We utilized two methods to calculate the seroprevalence of SARS-CoV-2 WT and variants: (1) The first approach was a traditionally applied method where the mean and standard deviation (STDV) of the negative controls is calculated and then the STDV multiplied by 3 and added to the mean (mean+3*STDV=3STDV threshold) and set as cut off. This method assumes the reads of the negative controls are normally distributed. Since the MFIs of our negative controls were skewed (i.e., not normally distributed data) we attempted to log transformed as previously described (22). However, log(10) transforming the MFI values of the negative controls for each antigen/isotype skewed the distribution even more, so the raw values were used to calculate the 3SDTV, likely underlining the inadequacy of this method for other data sets since negative controls will tend to accrue around zero or the lower limit of detection of the applied assay. Note, negative MFI values (artifact of the assay and not meaningful outcomes since there cannot be a negative value of antibodies in the samples) were set to 1.

Formatted: Not Highlight

Formatted: Indent: First line: 0"

Formatted: Indent: First line: 0"

Formatted: Indent: First line: 0"

Similarly, values of zero (n=6) were set to 1 to be able to show the spread of the data in log(10). See Supplemental Figure 1 for the raw MFI of the negative and positive controls, along with the calculated 3STDV thresholds. ~~See Supplemental Figure 1 for the raw MFI of the negative and positive controls, along with the calculated 3STDV thresholds.~~ (2) The second approach is a standardized open-source machine learning-based method that calculates 1-, 2-, and 3-dimensional classification boundaries based on confirmed positive and negative controls, as previously described (i.e., SeroNIST) (20–23). Each dimension corresponds to the number of antigens included, (i.e., equivalent to 1, 2, or 3 antigens). For example, 3 antigens covered WT RBD, N, and S to estimate SARS-CoV-2 seroprevalence, see Supplemental Figure 2. ~~(Table 4 and Supplemental Figure 2).~~ The resulting classification boundary is used as threshold, same as for the 3STDV method. For the SeroNIST method, raw MFI values were used (no adjustment for negative values or zeros necessary). The serological platform was evaluated, validated, and used for SARS-CoV-2 seroprevalence studies prior to this, see following references (23–25).

Results

Study Population and Demographics

A total of 745 participants were recruited from Owo in Ondo State (n=375) from October to November 2022 and from Ikorodu in Lagos State (n=370) from November 2022 to February 2023. ~~About~~ Over half of the participants were female (n=430, 57.7%), while 28.9% (n=215) were male, and 13.4% (n=100) did not report sex. Ages ranged from 0 to 84 years (~~average~~mean: 35.1 years, STDV: 14.7 years)~~, while the majority of participants were between 18 and 45 years of age (n=497, 66.7%)~~. The majority of participants had a secondary education (n=389, 52.2%), followed by those with university education (n=164, 22.0%), and then those with primary school education (n=130, 17.4%). A total of 54 individuals (7.2%) did not report education. Christianity was the dominant self-reported religion among our participants (n=570, 76.5%), followed by Islam (n=138, 18.5%), and those who practised other religions (n=36, 4.8%). Only one person reported not practicing any religion (0.1%). These

Formatted: Not Highlight

Formatted: Not Highlight

Formatted: Not Highlight

Formatted: Not Highlight

Formatted: Not Highlight

Formatted: Not Highlight

Formatted: Font: Bold

sociodemographic variables are also described in Supplemental Table 1 through Supplemental Table 5. -

Vaccine Uptake

Among those who reported vaccine status (n=739),The the majority (n=492, 66.6%)of participants reported having received either a single or double dose of the COVID-19 vaccine (n=492, 66.0%), while (n=247 (,33.24%) reported not being not vaccinated,) and. Note, six participants (0.8%) were missing vaccine responseopted out of reporting COVID-19 vaccine uptake, see Supplemental Table 6. The majority of the participants from Owo (peri-urban, Ondo State) were vaccinated (n=300, 80.2%, Supplemental Table 71). The vaccine uptake in Ikorodu (urban, Lagos State) was also relatively high (n=192, 52.6%) but lower than in Owo. The difference in regional vaccine uptake was statistically significant (Pearson's Chi-squared test with Yates' continuity correction), where peri-urban study participants from Owo were more likely to be vaccinated than the urban participants from Ikorodu (Chi-square statistic=62.1, df=1, p-value < 0.001). To determine whether sex, age, education, religion, state of residency, or being positive for anti-N SARS-CoV-2 antibodies independently increased or decreased the odds of having received a COVID-19 vaccine, we ran a multivariable analysis (i.e., logistic regression). To converge the model, religion and education categories with very small sample sizes (n=1 for no religion and n=8 for no formal education) were collapsed into larger categories for this analysis, see Supplemental Table 8 and Supplemental Table 9. Residency (Owo), age, and being positive for anti-N WT SARS-CoV-2 antibodies were all independently statistically significant and thus associated with vaccination status, see Figure 1 and Supplemental Table 10. Hence, individuals from Owo (aOR = 3.9, p-value < 0.0001) and older study participants (aOR = 1.1, p-value < 0.0001) had significantly higher odds of being vaccinated. Participants positive for anti-N SARS-CoV-2 antibodies (aOR = 0.5, p-value < 0.002) were much less likely to be vaccinated than participants negative for anti-N WT SARS-CoV-2 antibodies. We reran the analysis including age as categorial variable to confirm

Formatted: Font: Bold

Formatted: Not Highlight

Formatted: Not Highlight

these outcomes, see Supplemental Table 11. Residency (Owo), increased age, and being positive for anti-N WT SARS-CoV-2 antibodies were statistically significant again, see Supplemental Table 12. Individuals who were missing sex descriptors were also statistically significant, but the effect was barely significant (p-value = 0.043) and only observed when age was included as categorical variable. Thus, in summary, residents from Owo and study participants of older age had much higher odds of being vaccinated than residents from Ikorodu and study participants below the age 15. Further, individuals who had anti-N SARS-CoV-2 antibodies (reflecting natural infection) had lower odds of being vaccinated than to those who were negative for anti-N SARS-CoV-2 antibodies. Finally, sex, education, and religion did not influence vaccine uptake in our analysis. The difference in vaccine uptake by region was statistically significant (Pearson's Chi-squared test with Yates' continuity correction), where peri-urban study participants from Owo were more likely to be vaccinated than the urban participants from Ikorodu (Chi-square statistic=62.1, df=1, p value < 0.001). Among the individuals who reported sex, there was no statistically significant difference comparing the vaccinated and unvaccinated participants (Chi-square statistic = 3.1, df = 1, p value = 0.077). Among the individuals who reported education and religion, we found that there was a statistically significant difference comparing the vaccinated and unvaccinated participants, though marginally (education, Chi-square statistic = 8.5, df = 3, p value = 0.038); religion, Chi-square statistic = 8.0, df = 3, p value = 0.047). We then assessed whether there is an association between age and vaccine uptake. We found that, among our study population, increasing age was strongly associated with the likelihood of being vaccinated (logistic regression, age outcomes: β estimate = 0.047, p value < 0.001). For every 1-year increase in age, the odds of being vaccinated increased by ~4.8% (OR = 1.048, unadjusted for other factors). Note also that the percent of vaccinated individuals is higher in older age categories (Table 1). The association between vaccine uptake and age also remained when adjusting for region (logistic regression, age outcomes: β estimate = 0.050, p value < 0.001). Since the COVID-19 vaccine campaigns covering our study period focused mostly on adults,

Formatted: Not Highlight

we reran the analysis excluding children. The age-vaccine uptake association held true even when the analysis was restricted to those who were 18 years or older. Hence, increasing age was strongly associated with the likelihood of being vaccinated among adults (logistic regression, age outcomes: β estimate = 0.030, p value < 0.001), again not adjusting for other factors.

Figure 1. Adjusted odds ratios for predictors of COVID-19 vaccine uptake. The figure shows adjusted odds ratios (aORs) for vaccine uptake in log scale by evaluated predictors (state, age, sex, education, religion, and anti-N SARS-CoV-2 seropositivity). Owo residency and age were strongly associated with higher odds of vaccination (p-value < 0.0001), while seropositivity had lower odds of vaccination (p-value = 0.002). Sex, education, and religion were not significantly associated with vaccine uptake. Comparing the presence of anti-SARS-CoV-2 antibodies between those who reported being vaccinated and those who were not vaccinated revealed that vaccinated participants had significantly more anti-RBD antibodies against the WT, Beta, Gamma, Lambda, and Omicron variants, though some differences were marginal (Figure 1). The most significant difference between the vaccinated and unvaccinated group was for RBD Beta (p=<0.0001). Interestingly, the anti-N WT antibody measurements were similar comparing the vaccinated and unvaccinated groups, suggesting that both groups had been exposed equally to natural infections prior to study begin (likely covering 1-2 years prior to study based on previously published antibody decay estimates (26,27).

Formatted: Not Highlight
Formatted: Font: Bold, Not Highlight
Formatted: Font: Bold
Formatted: Font: Not Bold
Formatted: Font: Not Bold
Formatted: Font: Not Bold
Formatted: Font: Not Bold
Formatted: Font: Not Bold
Formatted: Font: Not Bold
Formatted: Font: Not Bold
Formatted: Font: Not Bold
Formatted: Font: Not Bold
Formatted: Font: Not Bold
Formatted: Font: Bold

Table 1. Percent and count of study participants by vaccination status in each sociodemographic and regional stratum.

Variable	aOR	95% CI (upper, lower)	p-value
State, Ondo	3.93	2.61, 6.00	1.0964E-10***
Age	1.05	1.04, 1.06	4.3563E-13***
Sex, Female	1.14	0.78, 1.66	0.4971
Sex, Missing	1.65	0.84, 3.37	0.1545
Education, Primary	0.79	0.38, 1.65	0.5363
Education, Secondary	0.92	0.49, 1.72	0.7860
Education, University	0.95	0.46, 1.95	0.8931

Formatted: Font: Not Bold

~~Religion, Islam~~	~~1.20~~	~~0.77, 1.89~~	~~0.4349~~
~~Religion, Other~~	~~0.87~~	~~0.40, 1.88~~	~~0.7133~~
~~N Positive~~	~~0.55~~	~~0.37, 0.80~~	~~0.0024**~~

~~aOR, adjusted odds ratio; ***, p-value < 0.0001; **, p-value <0.01~~

Comparing the presence of anti-RBD SARS-CoV-2 antibodies between those who reported being vaccinated and those who were not vaccinated revealed that vaccinated participants had significantly more anti-RBD antibodies against the WT, Beta, Gamma, Lambda, and Omicron variants (Figure 2). The most significant difference between the vaccinated and unvaccinated group was for RBD Beta (p-value < 0.0001).

Figure 2~~1~~. Anti-SARS-CoV-2 variant antibodies among vaccinated and unvaccinated study participants. The graph shows raw median fluorescence intensity (MFI) outcomes by variant and the respective geometric means and 95% confidence intervals (CI). Vaccinated participants had significantly higher antibody levels against the wildtype (WT) RBD (p-value = 0.013), RBD Beta (p-value =< 0.0001), RBD Gamma (p-value = 0.019), RBD Lambda (p-value = 0.001~~07~~), and RBD Omicron (p-value = 0.004) variants, but not RBD Alpha, RBD Delta, and WT Spike (S). ~~There was no difference between vaccinated and unvaccinated study participants for the anti-Nucleoprotein (N) wildtype (WT, Wuhan) antibodies.~~ Note, negative MFI values (artifact of the assay and not meaningful outcomes since there cannot be a negative value of antibodies in the samples) were set to 1 to plot the log(10) scale graph and better visualize the full spread of MFIs. Similarly, the few outcomes that ended up being 0 (n=3 study samples, Omicron only) were set to 1 for the same reasons. Vax, Vaccinated. MFI, median fluorescence intensity. RBD, receptor binding domain. Ns, not significant.

Formatted: Not Highlight

SARS-CoV-2~~CoV~~ Seroprevalence among Unvaccinated Study Participants

Next, we determined the SARS-CoV-2 seroprevalence among unvaccinated study participants using two different methods. We utilized two methods to calculate the seroprevalence of SARS-CoV-2 variants in Owo (Ondo) and Ikorodu (Lagos) in Nigeria. Thus, we compared two different strategies to calculate thresholds and determine which samples are considered positive or negative according to the calculated cut-offs: (1) The first approach was a traditionally applied method where the threshold is set at three standard deviations above the mean of the negative controls (3STDV). (2) The second approach was based on a standardized open-source machine learning-based method that calculates sensitive and specific classification boundaries based on population-specific pre-validated positive and negative controls and gives individual and group-based uncertainty estimates for the seroprevalence outcomes (i.e., SeroNIST) (22–25). We focused on calculating SARS-CoV-2 seroprevalence among the unvaccinated study population because we aimed to determine the proportion of participants who had been infected by SARS-CoV-2 (i.e., harbour infection-induced antibodies and not vaccine-induced antibodies).

Overall, the SARS-CoV-2 seroprevalence for anti-S and anti-RBD antibodies across all variants, except Omicron, was high (ranging from 89.1% to 100.0%, Table 12) and similar comparing the two methods among the unvaccinated study population. The seroprevalence for anti-RBD antibodies for the Omicron variant resulted in the most significant discrepancy and resulted in 82.2% (+/- 10.2 95% CI) for the SeroNIST method and 10.5% for the 3STDV method. Note that the SeroNIST method calculates thresholds by utilizing measurements from positive and negative controls (whereas the 3STDV approach is based on only the negative controls) and the positive controls were cases captured before Omicron circulated in the local population (see discussion for more details). The seroprevalence for anti-N WT antibodies resulted in 78.9% (+/- 7.7 95% CI) for the SeroNIST method and 66.4% for the 3STDV method.

Figure 2. Anti-SARS-CoV-2 variant antibodies among unvaccinated study participants (n=247). The graph shows raw median fluorescence intensity (MFI) outcomes by variant and

Formatted: Indent: First line: 0"

the respective geometric means and 95% confidence intervals (CI). Note, negative MFI values (artifact of the assay and not meaningful outcomes since there cannot be a negative value of antibodies in the samples) were set to 1 to plot the log(10) scale graph and better visualize the full spread of MFIs. Similarly, the few outcomes that ended up being 0 (n=3 study samples, Omicron only) were set to 1 for the same reasons. MFI, median fluorescence intensity. S, Spike. RBD, receptor binding domain. N, Nucleoprotein.

Table 12. SARS-CoV-2 Seroprevalence among unvaccinated participants (n=247) by threshold assessment method (3STDV vs. SeroNIST vs. 3STDV above the mean of negatives).

	3STDVSeroNIST	SeroNIST 3STDV		
	Seroprevalence %Seroprevalence % (95% CI)	Seroprevalence % (95% CI)Seroprevalence %	Sensitivity (95% CI)	Specificity (95% CI)
N WT	66.478.9 (+/- 7.7)	78.9 (+/- 7.7)66.4	100.0 (98.8, 100.0)	72.2 (59.6, 84.2)
S WT	91.9100 (+0/-3.4)	100.0 (+0/-3.4)91.9	100.0 (98.8, 100.0)	NA
RBD WT	95.1100 (+0/-8.9)	100.0 (+0/-8.9)95.1	100.0 (98.3, 100.0)	NA
RBD Alpha	89.1100 (+0/-7.0)	100.0 (+0/-7.0)89.1	100.0 (98.8, 100.0)	NA
RBD Beta	96.0100 (+0/-3.4)	100.0 (+0/-3.4)96.0	100.0 (98.8, 100.0)	NA
RBD Gamma	97.2100 (+0/-5.6)	100.0 (+0/-5.6)97.2	100.0 (98.8, 100.0)	NA
RBD Delta	99.699.6 (+/- 3.2)	99.6 (+/- 3.2)99.6	100.0 (98.8, 100.0)	NA
RBD Lambda	98.0100 (+0/-2.8)	100.0 (+0/-2.8)98.0	100.0 (98.8, 100.0)	NA
RBD Omicron	10.582.2 (+/- 10.2)	82.2 (+/- 10.2)10.5	100.0 (98.8, 100.0)	80.6 (68.2, 91.5)

Formatted: Centered
Formatted Table
Formatted: Font: (Default) Arial
Formatted: Font: (Default) Arial
Formatted: Font: (Default) Arial
Formatted: Font: (Default) Arial
Formatted: Font: (Default) Arial
Formatted: Font: (Default) Arial
Formatted: Font: (Default) Arial
Formatted: Font: (Default) Arial
Formatted: Font: (Default) Arial
Formatted: Font: (Default) Arial
Formatted: Font: (Default) Arial
Formatted: Font: (Default) Arial
Formatted: Font: (Default) Arial
Formatted: Font: (Default) Arial
Formatted: Font: (Default) Arial
Formatted: Font: (Default) Arial
Formatted: Font: (Default) Arial
Formatted: Font: (Default) Arial
Formatted: Font: (Default) Arial
Formatted: Font: (Default) Arial
Formatted: Font: (Default) Arial

NA, not applicable because there were too few negative outcomes to evaluate specificity for this analysis.

When breaking down N- and RBD WT-based seroprevalence by sociodemographic variables among unvaccinated study participants (using the SeroNIST method), the outcomes were similar comparing age groups, sex, education levels, and religious affiliation, see Table To determine whether sex, age, education, religion, city of residence, or vaccination status independently increased or decreased the odds of being positive for anti-N WT SARS-CoV-2 antibodies, we ran a multivariable analysis (i.e., logistic regression with binary seropositivity outcomes, see Supplemental Table 8, Supplemental Table 9, and Supplemental Table 13). We focused on the N-based seroprevalence because all the COVID-19 vaccines distributed in Nigeria covered S/RBD but not N antigens. Thus, the presence of anti-N SARS-CoV-2 antibodies were predictive of natural SARS-CoV-2 infection and not vaccination in this setting. Further, the RBD- and S-based seroprevalences were very high (ranged from 82.2% to 100%) and preclude a meaningful analysis (i.e., not enough negative outcomes, see Table 1). When running the multivariable analysis with age as continuous variable, 3. Hence, the risk of infection (reflected by N- and RBD WT-based seroprevalence among unvaccinated individuals) seemed even and high across the evaluated sociodemographic variables. being from Owo (aOR = 1.5, p-value = 0.045), being female (aOR = 1.4, p-value = 0.049), and being vaccinated (aOR = 0.5, p-value = 0.001) were independently statistically significant and were therefore associated with N WT SARS-CoV-2 serology, see Figure 3 and Supplemental Table 14. Thus, residents from Owo and female study participants had higher odds of being anti-N WT SARS-CoV-2 antibody positive, though those effects were barely statistically significant. Vaccinated individuals were much less likely to be N WT SARS-CoV-2 antibody positive (i.e., of being infected with SARS-CoV-2), and that effect was strong. There was also a strong and association between N WT SARS-CoV-2 seropositivity and not reporting sex (aOR = 7.0, p-value < 0.0001). We reran the analysis including age as categorial variable to confirm these outcomes. Residency (Owo), being female, being vaccinated, and missing a

sex descriptor were statistically significant again, see Supplemental Table 15. Thus, in summary, vaccinated individuals had much lower odds of being SARS-CoV-2 positive (likely indicating a protective effect of the vaccine), while there was a mild association between SARS-CoV-2 seropositivity and living in Owo or being female. Age, education, and religion did not influence serological positivity in our analysis. Finally, those who did not report sex were much more likely to be positive for anti-N WT SARS-CoV-2 antibodies, indicating a non-random pattern in missing data.

Figure 3. Adjusted odds ratios for predictors of anti-N SARS-CoV-2 seropositivity. The figure shows adjusted odds ratios (aORs) for anti-N wildtype (WT) SARS-CoV-2 seropositivity in log scale by evaluated predictors (state, age, sex, education, religion, and COVID-19 vaccine uptake). Vaccinated individuals had much lower odds of being positive (p-value = 0.001). Owo residency (p-value = 0.045) and being female (p-value = 0.049) were associated with higher odds of vaccination, though the effects were barely significant. Those who did not report sex were much more likely to be positive for anti-N WT SARS-CoV-2 antibodies (p-value < 0.0001). Age, education, and religion did not influence serological positivity.

Formatted: Font: Bold

~~**Table 3**. N- and RBD-based SARS-CoV-2 seroprevalence among unvaccinated study participants (n=247) by age, sex, education, and religion.~~

Age, education, and religion did not influence serological positivity. Category	Sub-group	Seroprevalence % (95 CI)	
-	-	N WT	RBD WT
Age group	0-17 years	88.4 (+/- 15.0)	100.0 (+0/- 20.6)
	18-30 years	75.0 (+/- 13.7)	100.0 (+0/- 14.9)
	31-45 years	76.0 (+/- 14.0)	100.0 (+0/- 16.0)
	>45 years	79.5 (+/- 21.6)	100.0 (+0/- 25.4)
-	-	-	-
Sex	Male	70.2 (+/- 13.6)	100.0 (+0/- 15.1)
	Female	82.1 (+/- 9.8)	100.0 (+0/- 11.8)
-	-	-	-
Education	Primary	80.5 (+/- 17.6)	100.0 (+0/- 20.9)
	Secondary	78.0 (+/- 10.5)	100.0 (+0/- 12.2)
	University	91.8 (+/- 16.2)	100.0 (+0/- 22.1)
-	-	-	-
Religion	Christianity	82.6 (+/- 8.8)	100.0 (+0/- 10.7)
	Islam	73.5 (+/- 17.1)	100.0 (+0/- 19.2)

Note: The sample size was too small for missing categories, and "other religion" so they were omitted for this table/analysis.

When comparing the two cities based on the SeroNIST method, the SARS-CoV-2 seroprevalence for anti-RBD WT was 100% for both (Table 4). For the anti-N WT (single antigen) and anti-RBD/S/N WT (three antigens combined) antibodies, the seroprevalence was higher in Owo (96.1% and 100.0% respectively) compared to Ikorodu (71.6% and 95.5% respectively).

Table 4. SARS-CoV-2 seroprevalence among unvaccinated individuals by city with SeroNIST method.

	Seroprevalence % (95 CI)	
	Ikorodu	Owo
N WT (1D)	71.6 (+/- 9.7)	96.1 (+/- 11.3)
RBD WT (1D)	100.0 (+0/-10.8)	100.0. (+0/-16.4)
RBD S N WT (3D)	95.5 (+/- 7.5)	100.0 (+0/-5.2)

Discussion

The COVID-19 pandemic, caused by SARS-CoV-2, rapidly spread across the globe in 2019/2020, resulting in millions of deaths, overwhelming healthcare systems, disrupting economies, and exposing vulnerabilities in global public health (27,28). Surprisingly, the number of cases and fatalities in Africa was low compared to the rest of the world (29). Nigeria, Africa's most populous country, accounted for less than 1% of the global cases (2). Whether these numbers reflect the true burden of SARS-CoV-2 is yet to be elucidated. The present study evaluated serosurvey-based SARS-CoV-2 exposure, compared serological threshold assessment approaches, determined the risk factors for SARS-CoV-2 infection, and evaluated the COVID-19 vaccine uptake among febrile Southern Nigerian study participants across urban and peri-urban settings in 2022/2023. These study results offer insights into sociodemographic and regional risk factors for SARS-CoV-2 infection, strategies to optimize serological study design, and approaches to improve COVID-19 vaccine uptake in West Africa.

Study Population

Formatted: Font: Bold

We found slightly higher female involvement and most of the participants to ~~fall into the young/middle-aged group (18–45 years), ~~be between 25 and 44 years old, which aligns with previous studies showing greater engagement in public health research by women and socially active populations (30,31). This was also reflected in the educational status of the participants, where most people had received post-primary formal education.

Vaccine Uptake

Formatted: Font: Bold

~~Among those who reported vaccine status, ~~T~~t~~he majority of study participants (66.06%) reported receiving a single or double dose of the COVID-19 vaccine, which is above the previously reported COVID-19 vaccine uptake in Nigeria, ranging from 20%-58% (32). While prior research indicates that sociodemographic factors significantly influence vaccine uptake

(33,34)(35,36), we did not find sex, education, or religion to be strong indicators for COVID-19 vaccine uptake in our study population. However, we found that peri-urban study participants from Owo were more likely to be vaccinated compared to urban counterparts from Ikorodu, indicating differential COVID-19 vaccine uptake across urban gradients in our study area. Since basic socioeconomic factors were not significant determinants of vaccine uptake in our study population, it may have been due to variation in COVID-19 vaccine distribution and accessibility or other factors our study did not capture. Furthermore, we found that older age was strongly associated with the likelihood of being vaccinated, likely reflecting the local vaccine campaign efforts during the study period, which focused on 60+ adults and 50+ adults with co-morbidities(37).

Interestingly, study participants who were anti-N SARS-CoV-2 antibody positive (reflecting natural infection) had much lower odds of being vaccinated. The statistically significant relationship between vaccine uptake and anti-N SARS-CoV-2 antibody positivity was confirmed through subsequent analyses (serology as dependent variable), where vaccinated individuals were much less likely to be N SARS-CoV-2 antibody positive. The effect in both models was strong and may indicate a protective effect of the vaccine, supported by finding higher anti-RBD WT antibodies among vaccinated study participants. The effect could also reflect or be compounded by behavioural modifications where cautious individuals were both more likely to get vaccinated and better at social distancing and hand-washing protocols, therefore preventing SARS-CoV-2 infection during the study period.

SARS-CoV-2 Serosurvey

Vaccinated participants had significantly more anti-RBD antibodies for the WT, Beta, Gamma, Lambda, and Omicron variants (Figure 2~~1~~). Detecting more anti-S/RBD antibodies in the vaccinated group likely validated the self-reported vaccination status since (i) being vaccinated has been described to elicit a stronger anti-RBD/S antibody response than natural infection, and (ii) being both vaccinated and infected is likely to induce a higher antibody response than

Formatted: Font: Bold

either alone (8,38). However, antibody decay should be taken into account (39,40), and we did not have information on time since vaccination or infection. ~~Interestingly, the anti-N WT antibody measurements were similar comparing the vaccinated and unvaccinated groups, suggesting that both groups were exposed equally to natural infections prior to our study.~~

SARS-CoV-2 Serosurvey Among Unvaccinated Study Participants

We assessed SARS-CoV-2 seroprevalence among unvaccinated study participants to evaluate SARS-CoV-2 exposure ~~and distinguish between vaccine- and natural infection-induced anti-RBD/S antibodies. Thus, the S/RBD and N-based seroprevalences likely represent natural infections since the analysis only included participants who reported not getting vaccinated.~~(vs. vaccination). Our study revealed high S/RBD-based seroprevalences for all variants except Omicron among the unvaccinated participants (ranging from 89.1% to 100%), indicating that the level of SARS-CoV-2 exposure was likely underestimated in Nigeria and confirming that reduced viral transmission was not likely the main reason the reported COVID-19 morbidity and mortality were low in Nigeria and the region (6). This is the highest seroprevalence rate for SARS-CoV-2 reported in Nigeria to date (11–16). The variation may reflect differences in sampling period and testing approach, advocating standardized serological methods. While most of the prior serosurveys were implemented between 2020 and mid-2022, our study happened later in the pandemic, between the end of 2022 and beginning of 2023. A similar high seroprevalence of over 90% was reported in Yemen in 2023 (41).

Furthermore, Nigerians may have been exposed to SARS-CoV-2 variants that were not previously reported in the country. Nigeria experienced four major SARS-CoV-2 waves dominated by the ancestral/WT, Alpha, Delta, Eta, and Omicron variants (17–20). Other important variants detected elsewhere, such as Gamma and Lambda variants, were not

Formatted: Font: Bold

Formatted: Indent: First line: 0"

reported in Nigeria (42), but we detected relatively high levels of anti-RBD antibody for both variants among our study population, although some of the signal may have been due to cross-reactivity. While the affinity between the specific variant antigens (i.e., antigens coupled to the beads in our serological assay) and respective human antibodies should be the highest/tightest, we could not preclude cross-reactivity between SARS-CoV-2 variants and also with endemic human CoVs (e.g., HKU1, NL63, OC43, and 229E) due to lack of positive controls (i.e., individuals who were exposed only to specific SARS-CoV-2 variants, HKU1, NL63, OC43, or 229E). The low seroprevalence and antibody levels for Omicron RBD detected in our study may have been due to low transmission or due to a distinct serological footprint (43). Further genomic epidemiological approaches and proactive serosurveillance will help unravel the true burden of all SARS-CoV-2 variants in Nigeria.

Risk Factors for SARS-CoV-2 Infection

Formatted: Font: Bold

As forWe evaluated risk factors for SARS-CoV-2 infection through an anti-N WT SARS-CoV-2 serosurvey-based multivariable analysis across all study participants (vaccinated and unvaccinated). Since vaccines distributed in Nigeria during the study period covered S/RBD but not N antigens), we focused on the N-based seroprevalence to estimate risk factors for natural SARS-CoV-2 infection (vs. vaccination). Further, the RBD- and S-based seroprevalences where high and ranged from 82.2% to 100.0%, precluding a meaningful risk factor analysis.

As mentioned above, vaccinated individuals were much less likely to be N WT SARS-CoV-2 antibody positive (p-value = 0.001). Female study participants had higher odds of being positive for anti-N WT SARS-CoV-2 antibodies, though the effect was barely statistically significant (p-value = 0.049). Age, education, and religion did not influence the serological outcome. Thus, we , we did not find compelling high-risk sociodemographic subpopulations based on age, sex, educational status, or religion, similar to previous studies (11,30). Finally, Owo residents were more likely to be N WT SARS-CoV-2 positive. Though the effect was

barely statistically significant (p-value = 0.045), study participants living in peri-urban settings were at higher risk of SARS-CoV-2 infection compared to urban settings (Ikorodu). When comparing RBD/S-based SARS-CoV-2 seroprevalence between Ikorodu and Owo, we also did not find a difference. When comparing N-based SARS-CoV-2 seroprevalences, however, we found higher seroprevalence for Owo (96.1%) compared to Ikorodu (71.6%), which may reflect the timing of the most recent wave prior to the study beginning since the anti-N antibodies have a shorter half-life than RBD/S (44). Hence, since the RBD/S- and overall RBD/S/N-based seroprevalence is similar in both cities, there is likely no real difference in risk of exposure to SARS-CoV-2. Since the N-based seroprevalence is higher in Owo, that region may have experienced a more recent wave compared to Ikorodu, where the anti-N-based antibodies may already have decayed below the detection threshold for parts of the local population when we collected the samples.

Serological Threshold Assessment

Our study compared two threshold calculation methods to evaluate the SARS-CoV-2 seroprevalence. There was concordance between the two approaches, except for anti-RBD Omicron and anti-N WT antibodies. The difference between the N-based seroprevalence was within ~10% when considering the 95%CI, but the Omicron-based seroprevalence differed widely (82.2% vs. 10.5%). The SeroNIST method calculates thresholds by utilizing measurements from positive and negative controls (whereas the 3STDV approach is based on only the negative controls) and the positive controls were cases captured before Omicron circulated in the local population. Hence, they were not good positive controls to determine the SeroNIST-based thresholds for Omicron. Note that the SeroNIST method calculates thresholds by utilizing measurements from positive and negative controls (whereas the 3STDV approach is based on only the negative controls) and the positive controls were cases captured before Omicron circulated in the local population (see discussion for more details). The seroprevalence for anti-N WT antibodies resulted in 78.9% (+/- 7.7 95% CI) for the SeroNIST method and 66.4% for the 3STDV method. This was likely due to the positive

Formatted: English (United States)

Formatted: Indent: First line: 0"

Formatted: Font: Bold

controls, which were collected pre-Omicron, so they were not good controls to determine the SeroNIST-based thresholds for Omicron, which is based on positive and negative controls (whereas the 3STDV is based only on negative controls). Overall, the two methods ended up resulting inestimated similar seroprevalence outcomes (except for Omicron, where no appropriate positive controls were available). However, , and the SeroNIST method represents a more rigorous and standardized approach. It automatically calculates seroprevalence and the associated uncertainty estimates (sensitivity, specificity, and accuracy) for the population-based and individual serological outcomes. based on an open-source tool that provides uncertainty estimates for population-based and individual serological outcomes. Using an open source, standardized, and automated approach across serosurveys (independent of the pathogen of interest) will make the seroprevalence calculations quicker, more informative, and comparable across studies, making the outcomes more suitable for metanalysis and increasing the public health impact of serosurveys overall. Hence, we considered the SeroNIST method superior to the 3SDTV approach.Using a standardized approach across serosurveys (independent of the pathogen of interest) will make results comparable and suitable for metanalysis, increasing the public health impact of serosurveys in general.

Limitations

Our study results should be viewed considering a few limitations.

For example, whileWhile we were able to enroll 745 study participants, increasing the limited sample size, including additional socioeconomic variables, and being able to access vaccination dates would have given a more nuanced picture of risk factors for SARS-CoV-2 exposure and COVID-19 vaccine uptake. Unfortunately, that information was not available to us. Further, the socio-demographic information and vaccine uptake were self-reported which may lead to recall bias or misrepresentations.

We had missing data for vaccine uptake (n=6, 0.8%), sex (n=100, 13.4%), education (n=54, 7.2%), and age (n=1/275). All the missing data was included as separate categories in the regression analyses, except for age. There were no strong and significant outcomes for the

Formatted: Font: Bold

missing data categories except for sex. Those who did not report sex were much more likely to be positive for anti-N SARS-CoV-2 antibodies, indicating a non-random pattern in missing data. Since anti-N SARS-CoV-2 antibody positivity was not a self-reported outcome however, it was not likely to be directly related. ~~and including additional socioeconomic variables would have given a more nuanced picture of risk factors for SARS-CoV-2 exposure and COVID-19 vaccine uptake.~~

~~Furthermore~~Importantly, our study only covered symptomatic participants who visited local healthcare centers. Hence, it does not represent the general population. Further, individuals who visit health care centers when sick may be more open to engage with the public health system and therefore may be more likely to get vaccinated compared to the general population.

Finally, t~~T~~he main methodological limitation of our study was the inability to account for potential serological cross-reactivity between SARS-CoV-2 variants and potentially other CoVs due to the lack of positive controls for each CoV subtype.

Conclusion and Recommendations

Formatted: Font: Bold

(i) The results of this study suggest that the SARS-CoV-2 exposure in Southern Nigeria was higher than previously reported. Hence, lack of SARS-CoV-2 transmission was not likely the reason that Nigeria, and potentially the region, experienced less COVID-19 disease and related deaths. Still, genomic surveillance is needed to track variance-based exposure patterns (44). (ii) The COVID-19 vaccine uptake was high, with peri-urban participants showing greater coverage than those in urban areas, and increasing age was a strong predictor of vaccination. Hence, the local vaccine uptake strategy implemented prior to 2022/2023 proved effective, resulting in high coverage rates and representing a significant public health success. Interestingly, our study found a strong correlation between being vaccinated and having lower odds of being positive for positive for anti-N WT SARS-CoV-2 antibodies (proxy for SARS-CoV-2 infection). Therefore, the vaccine may have protected study participants from SARS-CoV-2 infection prior to the study. Overall, ongoing public health

interventions should focus on improving vaccine uptake in urban populations and younger age groups while leveraging the observed success of peri-urban vaccine campaigns. (iii) The lack of standardized serological thresholding methods highlights the need for ~~standardized~~ robust analytical approaches. While there was concordance between the two approaches, prioritizing the use of validated, ~~and~~ standardized, and automated thresholding methods such as SeroNIST will enable reliable comparisons of serosurveys across studies and time, thereby increasing the public health impact of serosurveys.

Acknowledgements

We thank the study participants and their families for their valuable time and participation. Without them, this study would not have been possible.

Financial Support

Funding was provided by (1) the Centers for Disease Control and Prevention (CDC) through the U01GH002338-01 grant (RRD, CTH, and AMM) titled “Seroepidemiological Insight into COVID-19 Transmission in Africa (SICA)”, (2) the National Institute of Health (NIH) through the National Center for Advancing Translational Sciences KL2-TR001455 grant (RAB), and (3) the Bill and Melinda Gates Foundation through the GIISER Project ~~(~~ARISE-INV-036304 (CTH and CAU). The funders had no role in study design, data collection and analysis, decision to publish, or preparation of the manuscript.

Conflicts of Interest

The authors have declared that no competing interests exist.

References

1. Karcıoğlu O, Yüksel A, Baha A, Er AB, Esendağlı D, Gülhan PY, et al. Covid-19: The Biggest Threat of the 21st Century: In Respectful Memory of the Warriors All Over the World. Turk Thorac J. 2020 Dec 11;21(6):409–18.
2. WHO. COVID-19 Epidemiological Update. WHO; 2024.
3. Cabore JW, Karamagi HC, Kipruto HK, Mungatu JK, Asamani JA, Droti B, et al. COVID-19 in the 47 countries of the WHO African region: a modelling analysis of past trends and future patterns. Lancet Glob Health. 2022 Aug;10(8):e1099–114.
4. Maeda JM, Nkengasong JN. The puzzle of the COVID-19 pandemic in Africa. Science. 2021 Jan;371(6524):27–8.
5. Happi CT, Nkengasong JN. Two years of COVID-19 in Africa: lessons for the world.
6. Bejon P, Agweyu A, Oyier I, Hamaluba M, Kamuya D, Kinyanjui S, et al. Rethinking the Evidence on COVID in Africa. Lancet Infect Dis [Internet]. 2025 Apr 25 [cited 2025 June 21]; Available from: https://doi.org/10.1016/ S1473-3099(25)00071-4
7. Adams J, MacKenzie MJ, Amegah AK, Ezeh A, Gadanya MA, Omigbodun A, et al. The Conundrum of Low COVID-19 Mortality Burden in sub-Saharan Africa: Myth or Reality? Glob Health Sci Pract. 2021 Sept 30;9(3):433–43.
8. Ugwu CA, Alao O, John OG, Akinnawo B, Ajayi I, Odebode O, et al. Immunological insights into COVID-19 in Southern Nigeria. Front Immunol. 2024 Jan 23;15:1305586.
9. Ojo TO, Ojo AO, Ojo OE, Akinwalere BO, Akinwumi AF. Determinants of COVID-19 vaccine uptake among Nigerians: evidence from a cross-sectional national survey. Arch Public Health. 2023 May 26;81(1):95.

Formatted: Line spacing: Double

10. Ojeniyi T, Eguavoen A, Chinye-Nwoko F. Moving the needle for COVID-19 vaccinations in Nigeria through leadership, accountability, and transparency. Front Public Health. 2023 Sept 21;11:1199481.

11. Audu RA, Stafford KA, Steinhardt L, Musa ZA, Iriemenam N, Ilori E, et al. Seroprevalence of SARS-CoV-2 in four states of Nigeria in October 2020: A population-based household survey. Nelson MI, editor. PLOS Glob Public Health. 2022 June 17;2(6):e0000363.

12. Chechet GD, Kwaga JKP, Yahaya J, Noyes H, MacLeod A, Adamson WE. SARS-CoV-2 seroprevalence at urban and rural sites in Kaduna State, Nigeria, during October/November 2021, immediately prior to detection of the Omicron variant. Int J Epidemiol. 2022 Oct 13;51(5):1361–70.

13. Ige FA, Ohihoin GA, Osuolale K, Dada A, Onyia N, Johnson A, et al. Seroprevalence of SARS-CoV-2 IgG among healthcare workers in Lagos, Nigeria. Olu OO, editor. PLOS ONE. 2023 Oct 5;18(10):e0292440.

14. Kolawole OM, Tomori O, Agbonlahor D, Ekanem E, Bakare R, Abdulsalam N, et al. SARS CoV-2 Seroprevalence in Selected States of High and Low Disease Burden in Nigeria. JAMA Netw Open. 2022 Oct 11;5(10):e2236053.

15. Olayanju O, Bamidele O, Edem F, Eseile B, Amoo A, Nwaokenye J, et al. SARS-CoV-2 Seropositivity in Asymptomatic Frontline Health Workers in Ibadan, Nigeria. Am J Trop Med Hyg. 2021 Jan 6;104(1):91–4.

16. Akanmu S, Herrera BB, Chaplin B, Ogunsola S, Osibogun A, Onawoga F, et al. High SARS-CoV-2 seroprevalence in Lagos, Nigeria with robust antibody and cellular immune responses. J Clin Virol Plus. 2023 Aug;3(3):100156.

17. Olawoye IB, Oluniyi PE, Oguzie JU, Uwanibe JN, Kayode TA, Olumade TJ, et al. Emergence and spread of two SARS-CoV-2 variants of interest in Nigeria. Nat Commun. 2023 Feb 13;14(1):811.

18. Olorunfemi AB, Suliman SAR, Tran TT, Ayorinde B, Fowora MA, Iwalokun BA, et al. Whole genome sequencing and phylogenetic analysis of SARS-CoV-2 strains isolated during the COVID-19 pandemic in Nigeria. IJID Reg. 2024 Mar;10:174–8.

19. Kolawole DB, Okeke MI. Phylogenetic and genome-wide mutational analysis of SARS-CoV-2 strains circulating in Nigeria: no implications for attenuated COVID-19 outcomes. Osong Public Health Res Perspect. 2022 Apr 30;13(2):101–13.

20. Akande OW, Elimian KO, Igumbor E, Dunkwu L, Kaduru C, Olopha OO, et al. Epidemiological comparison of the first and second waves of the COVID-19 pandemic in Nigeria, February 2020–April 2021. BMJ Glob Health. 2021 Nov;6(11):e007076.

21. McConnell D, Hickey C, Bargary N, Trela-Larsen L, Walsh C, Barry M, et al. Understanding the Challenges and Uncertainties of Seroprevalence Studies for SARS-CoV-2. Int J Environ Res Public Health. 2021 Apr 27;18(9):4640.

22. Binder RA, Fujimor GF, Forconi CS, Reed GW, Silva LS, Lakshm P, et al. SARS-CoV-2 Serosurveys: How Antigen, Isotype and Threshold Choices Affect the Outcome. J Infect Dis. 2022 Oct 31;2023(227):371–80.

23. Binder RA, Matta AM, Forconi CS, Oduor CI, Bedekar P, Patrone PN, et al. Minding the margins: Evaluating the impact of COVID-19 among Latinx and Black communities with optimal qualitative serological assessment tools. Egorov AI, editor. PLOS ONE. 2024 July 25;19(7):e0307568.

24. Patrone PN, Binder RA, Forconi CS, Moormann AM, Kearsley AJ. Analysis of Diagnostics (Part I): Prevalence, Uncertainty Quantification, and Machine Learning

[Internet]. arXiv; 2024 [cited 2025 June 21]. Available from: http://arxiv.org/abs/2309.00645

25. Patrone PN, Binder RA, Forconi CS, Moormann AM, Kearsley AJ. Analysis of Diagnostics (Part II): Prevalence, Linear Independence, and Unsupervised Learning [Internet]. arXiv; 2024 [cited 2025 June 21]. Available from: http://arxiv.org/abs/2408.16035

26. Soetewey A. Binary logistic regression in R. Stats and R [Internet]. Stats and R. 2024. Available from: https://statsandr.com/blog/binary-logistic-regression-in-r/

27. Faramarzi A, Norouzi S, Dehdarirad H, Aghlmand S, Yusefzadeh H, Javan-Noughabi J. The global economic burden of COVID-19 disease: a comprehensive systematic review and meta-analysis. Syst Rev. 2024 Feb 16;13(1):68.

28. Nicola M, Alsafi Z, Sohrabi C, Kerwan A, Al-Jabir A, Iosifidis C, et al. The socio-economic implications of the coronavirus pandemic (COVID-19): A review. Int J Surg. 2020 June;78:185–93.

29. Joseph S, Kutty Narayanan A. Covid 19-the 21st Century Pandemic: The Novel Coronavirus Outbreak and the Treatment Strategies. Adv Pharm Bull. 2021 Jan 30;1.

30. Onifade AA, Fowotade A, Rahamon SK, Edem VF, Yaqub SA, Akande OK, et al. Seroprevalence of anti-SARS-CoV-2 specific antibodies in vaccinated and vaccine naïve adult Nigerians. Bhattacharya D, editor. PLOS ONE. 2023 Jan 23;18(1):e0280276.

31. Wu C, Qian Y. The gender peak effect: Women are most vulnerable to infections during COVID-19 peaks. Front Public Health. 2022 Aug 9;10:937179.

32. Olu-Abiodun O, Abiodun O, Okafor N. COVID-19 vaccination in Nigeria: A rapid review of vaccine acceptance rate and the associated factors. Elelu N, editor. PLOS ONE. 2022 May 11;17(5):e0267691.

33. Tchiasso D, Mendjime P, Njuwa Fai K, Nana Wandji BS, Yuya F, Youm É, et al. Dynamic factors associated with COVID-19 vaccine uptake in Cameroon between 2021 and 2022. J Public Health Afr [Internet]. 2024 Oct 24 [cited 2024 Dec 8];15(1). Available from: https://publichealthinafrica.org/index.php/jphia/article/view/578

34. White TM, Wyka K, Rabin K, El-Mohandes A. Trust in the science behind COVID-19 vaccines as a driver of vaccine acceptance in the United States, 2021–2023. Vaccine X. 2024 Dec;21:100576.

35. Costa JC, Weber AM, Darmstadt GL, Abdalla S, Victora CG. Religious affiliation and immunization coverage in 15 countries in Sub-Saharan Africa. Vaccine. 2020 Jan;38(5):1160–9.

36. Santos TM, Cata-Preta BO, Wendt A, Arroyave L, Hogan DR, Mengistu T, et al. Religious affiliation as a driver of immunization coverage: Analyses of zero-dose vaccine prevalence in 66 low- and middle-income countries. Front Public Health. 2022 Oct 25;10:977512.

37. Nomhwange T, Wariri O, Nkereuwem E, Olanrewaju S, Nwosu N, Adamu U, et al. COVID-19 vaccine hesitancy amongst healthcare workers: An assessment of its magnitude and determinants during the initial phase of national vaccine deployment in Nigeria. eClinicalMedicine. 2022 Aug;50:101499.

38. Kaplonek P, Deng Y, Shih-Lu Lee J, Zar HJ, Zavadska D, Johnson M, et al. Hybrid immunity expands the functional humoral footprint of both mRNA and vector-based SARS-CoV-2 vaccines. Cell Rep Med. 2023 May;4(5):101048.

39. Van Elslande J, Oyaert M, Lorent N, Vande Weygaerde Y, Van Pottelbergh G, Godderis L, et al. Lower persistence of anti-nucleocapsid compared to anti-spike antibodies up to one year after SARS-CoV-2 infection. Diagn Microbiol Infect Dis. 2022 May;103(1):115659.

40. Gallais F, Gantner P, Bruel T, Velay A, Planas D, Wendling MJ, et al. Evolution of antibody responses up to 13 months after SARS-CoV-2 infection and risk of reinfection. eBioMedicine. 2021 Sept;71:103561.

41. Taher WT, Bawazir AA, Sallam TA, Alsurimi K. Seroprevalence and factors associated with SARS-CoV-2 infection among healthcare workers: cross-sectional study. BMC Infect Dis. 2023 Nov 6;23(1):761.

42. Gozzi N, Chinazzi M, Davis JT, Mu K, Pastore Y Piontti A, Ajelli M, et al. Real-time estimates of the emergence and dynamics of SARS-CoV-2 variants of concern: A modeling approach. Epidemics. 2024 Dec;49:100805.

43. Qu P, Faraone JN, Evans JP, Zheng YM, Carlin C, Anghelina M, et al. Enhanced evasion of neutralizing antibody response by Omicron XBB.1.5, CH.1.1, and CA.3.1 variants. Cell Rep. 2023 May;42(5):112443.

44. Happi CT, Nkengasong JN. Two years of COVID-19 in Africa: lessons for the world.

Supplemental Figure 1. Controls and 3STDV Threshold. The graph shows raw median fluorescence intensity (MFI) outcomes of the positive controls (red dots) and the negative controls (blue dots) by SARS-CoV-2 variant. In green are the 3STDV thresholds (based on negative controls only), with respective values on top. Note, negative MFI values (artifact of the assay and not meaningful outcomes since there cannot be a negative value of antibodies in the samples) were set to 1 to plot the log(10) scale graph and better visualize the full spread of MFIs. MFI, median fluorescence intensity. S, Spike. RBD, receptor binding domain. N, Nucleoprotein.

Supplemental Figure 2. SeroNIST Classification Boundary based on three Antibodies. (A) This figure shows the classification boundary (i.e., three-dimensional threshold in rainbow

colours) created by the SeroNIST fool for wildtype (WT) RBD, S, and N antibodies based on the training data (i.e., negative control in blue dots and positive controls in red dots). (B) This figure shows where the data points (blue dots) fall for an example data set of participants. In this case, all the data points fall above the classification boundary, resulting in an estimated SARS-CoV-2 seroprevalence of ~100% based on RBD, S, and N antibodies.

Supplemental Table 1. Residency of study participants (n=745).

Supplemental Table 2. Sex distribution among study participants (n=745).

Supplemental Table 3. Age of study participants (by category, n=745).

Supplemental Table 4. Educational level of study participants (n=745).

Supplemental Table 5. Religion among study participants (n=745).

Supplemental Table 6. Vaccine uptake among study participants (n=745).

Supplemental Table 7. Vaccine uptake by region/state.

Supplemental Table 8. Education variable for multivariable analysis. No formal education (n=8) and primay education (n=130) were combined (n=138).

Formatted: Font: Not Bold

Supplemental Table 9. Religion variable for multivariable analysis. No religion (n=1) and Other (n=36) were combined (n=37).

Formatted: Font: Bold

Supplemental Table 10. Model outputs for vaccine uptake multivariable analysis (age as continuous variable).

Supplemental Table 11. Categorical age variable for vaccine uptake multivariable analysis.

Supplemental Table 12. Model outputs for vaccine uptake multivariable analysis (age as categorical variable).

Supplemental Table 13. N WT SARS-CoV-2 seropositivity among study participants.

Supplemental Table 14. Model outputs for N seropositivity multivariable analysis (age as continuous variable).

Supplemental Table 15. Model outputs for N seropositivity multivariable analysis (age as categorical variable).

Formatted: Font: Bold

Supplemental Figure 1. Controls and 3STDV Threshold. The graph shows raw median fluorescence intensity (MFI) outcomes of the positive controls (red dots) and the negative controls (blue dots) by SARS-CoV-2 variant. In green are the 3STDV thresholds (based on negative controls only), with respective values on top. Note, negative MFI values (artifact of the assay and not meaningful outcomes since there cannot be a negative value of antibodies in the samples) were set to 1 to plot the log(10) scale graph and better visualize the full spread of MFIs. MFI, median fluorescence intensity. S, Spike. RBD, receptor binding domain. N, Nucleoprotein.

Supplemental Figure 2. SeroNIST Classification Boundary based on three Antibodies. (A) This figure shows the classification boundary (i.e., three-dimensional threshold in rainbow colours) created by the SeroNIST fool for wildtype (WT) RBD, S, and N antibodies based on the training data (i.e., negative control in blue dots and positive controls in red dots). (B) This figure shows where the data points (blue dots) fall for an example data set of participants. In this case, all the data points fall above the classification boundary, resulting in an estimated SARS-CoV-2 seroprevalence of ~100% based on RBD, S, and N antibodies.

1. R Sa. Binary Logistic Regression in R 2024 [Available from: https://statsandr.com/blog/binary-logistic-regression-in-r/.

PGPH-D-25-02354

We thank the reviewers and editorial team for the feedback on our manuscript, "COVID19 Sero-epidemiology and Vaccine Uptake in Two Southern Nigerian Clinics" (PGPH-D-25-02354). The feedback has significantly improved the statistical approach, results description, formatting, and public health implication of the manuscript and applied analyses.

In response, we have substantially revised the manuscript. Please see below the integrated responses in blue. Note, the line numbers correspond to the manuscript in track changes. In summary, we improved the Methods section to provide more information on the serological method and its importance. We added extensive multivariable analyses to evaluate vaccine uptake and seropositivity in terms of adjusted odds ratios and included detailed outcomes in the Abstract, Methods, Results (including figures/forest plots), Discussion, and Supplemental Materials to give readers more details. We clarified the missing data and included it in the logistic regressions. Finally, we expanded the Discussion to address shortcomings, including the fact that our study population covered only symptomatic individuals.

We are grateful for the reviewers' insights, which have guided substantial improvements to both the scientific robustness and the practical significance of the paper.

Journal Requirements:

1. Please include a complete copy of PLOS' questionnaire on inclusivity in global research in your revised manuscript. The questionnaire has been filled out and included.

2. Please provide a detailed online Financial Disclosure statement. a. State the initials, alongside each funding source, of each author to receive each grant. For example: "This work was supported by the National Institutes of Health (####### to AM; ###### to CJ) and the National Science Foundation (###### to AM)." b. State what role the funders took in the study. The financial disclosure statement has been updated in the portal and in the manuscript.

3. Please ensure that the funders and grant numbers match between the Financial Disclosure field and the Funding Information tab in your submission form. Note that the funders must be provided in the same order in both places as well. This has been done.

4. We note that your Data Availability Statement is currently as follows: "The full database has been submitted along with the manuscript and figures." Please confirm at this time whether or not your submission contains all raw data required to replicate the results of your study. We confirm that the full database has been submitted.

5. Please provide separate main figure files in .tif or .eps format only and ensure that all files are under our size limit of 10MB. For more information about how to convert your figure files please see our guidelines: https://journals.plos.org/globalpublichealth/s/figures. We strongly recommend that you use PLOS's NAAS tool (https://ngplosjournals.pagemajik.ai/artanalysis) to test your figure files. The figures have been reformatted and checked with the PLOS's NAAS tool.

Editor Comments:

Please address the comments brought forth by the reviewers with a focus on including a more comprehensive multivariable analysis for factors associated with seroprevalence in the total study population and in the unvaccinated study population. The potential confounders included in the model should be justified based on literature and 95% CIs should be included in tables and in the text when referencing results from the regression analyses. Clear tables should be included in the manuscript that identify the results from the regression model(s). Also, stronger justification for excluding missing data should be included vs imputation. Where the patterns of missingness examined?

Thank you very much for the helpful feedback. To determine whether sex, age, education, religion, city of residence, or vaccination status independently increased or decreased the odds of being positive for anti-N WT SARS-CoV-2 antibodies, we now included a multivariable analysis (i.e., logistic regression with binary seropositivity outcomes, see Supplemental Table 8, Supplemental Table 9, Supplemental Table 13, and Figure 1). These variables and the associated literature are cited in the Discussion. We focused on the N-based seroprevalence because all the COVID-19 vaccines distributed in Nigeria covered S/RBD but not N antigens. Thus, the presence of anti-N SARS-CoV-2 antibodies were predictive of natural SARS-CoV-2 infection and not vaccination in this setting. Further, the RBD- and S-based seroprevalences were very high (ranged from 82.2% to 100%) and preclude a meaningful analysis (i.e., not enough negative outcomes, see Table 1). The outcomes now include adjusted odds ratios (aORs), respective 95% confidence intervals, and p-values. Further, to determine which of the included variables independently increase or decrease the odds of having received a COVID-19 vaccine, we ran a similar multivariable analysis. Those results and tables are also included in detail, see Figure 3, and Supplemental Table 14. The missing data was now analyzed and included as separate categories in the regressions, except for age, because a single participant was missing age.

Reviewer #1:
I have now completed my review of the paper and incorporated my comments directly into the text for your consideration. The main recommendations focus on:

Thank you very much for the detailed review and helpful feedback. The multivariable analysis and all the other suggestions were very helpful and added interesting and more robust results. All the comments have now been addressed, please see below in blue.

1. Inclusion criteria considering that sampling is limited to febrile outpatients, and so generalizability is reduced.

   Yes, unfortunately our study team only had access to febrile study participants which is not representative of the general population. The fact that our study population is febrile (i.e., symptomatic) is stated in the Introduction, Methods, Results, and discussed as limitation in the Discussion, see track changes and particularly lines 929-932.

2. The fact that vaccination data is self-reported. You may want to consider clarifying the risk of misclassification.

Thank you for that comment. We now included the following section in the Discussion on lines 890-895: "While we were able to enroll 745 study participants, increasing the limited sample size, including additional socioeconomic variables, and being able to access vaccination dates would have given a more nuanced picture of risk factors for SARS-CoV-2 exposure and COVID-19 vaccine uptake. Unfortunately, that information was not available to us. Further, the socio-demographic information and vaccine uptake were self-reported which may lead to recall bias or misrepresentations."

3. On the statistics, there is limited multivariate analysis. I recommend stronger modelling for uptake predictors.

Thank you for the comment. The manuscript now includes extensive multivariable analyses, adjusted odds ratios (aORs), respective 95% confidence intervals, and p-values in the main text, Figures, and Supplemental Materials.

4. No explicit herd immunity threshold or practical cold-chain/coverage was discussed. If possible, it should be reviewed.

Serum was separated onsite using portable centrifuges and stored -20 °C under cold-chain conditions. Each sample was labelled with a unique study identifier and linked to participant data through encrypted REDCap databases. Stored and processed samples were transported from the site using a dry shipper (pre-charged to -20 °C) to the ACEGID laboratory at Redeemer's University for bead-based multiplex serology (Luminex MagPix). This is now included in the Methods section from lines 210-215.

Comments:

1. Introduction - Could a clearer framing of why a sero-survey matters be incorporated? Is it possible to highlight the study's novelty such as variant-specific immunity, standardized thresholding and vaccine uptake linkage?

Thank you for the comment. We have now edited the introduction to clarify, see track changes throughout introduction.

1. Have some concerns with the inclusion criteria as it may not be fully representative of the general population and can introduce bias towards health-system users. Was there a reason for not using a not probability-based sampling.

Yes, unfortunately our study team only had access to febrile study participants which is not representative of the general population. The fact that our study population is febrile (i.e., symptomatic) is stated in the Introduction, Methods, Results, and discussed as limitation in the Discussion, see track changes and particularly lines 929-932.

2. There is clarity on the method described with antigen panels and controls. However, the performance metrics (sensitivity/specificity) has not been also clearly stated.

Thank you for that comment. We now include sensitivity and specificity in the seroprevalence

outcomes, see Table 1.

3. The assay workflow is well detailed, but was there a reason not to mention or not having a quality control procedure such as duplicate testing, external QC panels or inter-lab validation?

Thank you for the comment. In-depth validations, including across our teams in Nigeria and the US were done prior to the study. This included an in-person visit of Nigerian staff to the US for training purposes. Further, the serological platform was evaluated, validated, and used for SARS-CoV-2 seroprevalence studies prior to this, see references 23-25. This is now mentioned in the Methods.

4. Reported single/double doses and age focus, but no timing since vaccination, no booster details.

Thank you for that comment. Unfortunately, we did not have access to that information. We now included the following section in the Discussion on lines 890-895: "While we were able to enroll 745 study participants, increasing the limited sample size, including additional socioeconomic variables, and being able to access vaccination dates would have given a more nuanced picture of risk factors for SARS-CoV-2 exposure and COVID-19 vaccine uptake. Unfortunately, that information was not available to us. Further, the socio-demographic information and vaccine uptake were self-reported which may lead to recall bias or misrepresentations."

5. To access appropriately the risk factors/effectiveness could a multivariable logistic regression for vaccine uptake or infection risk be used beyond basic comparisons?

Thank you for the comment. The manuscript now includes extensive multivariable analyses, adjusted odds ratios (aORs), respective 95% confidence intervals, and p-values in the main text, Figures, and Supplemental Materials.

6. Reported single/double doses and age focus, but no timing since vaccination, no booster details. Also, the vaccine uptake model used here could have been made stronger if other social determinants data such as income, occupation, mobility or health literacy were not collected.

Thank you for that comment. Unfortunately, we did not have access to that information. We now included the following section in the Discussion on lines 890-895: "While we were able to enroll 745 study participants, increasing the limited sample size, including additional socioeconomic variables, and being able to access vaccination dates would have given a more nuanced picture of risk factors for SARS-CoV-2 exposure and COVID-19 vaccine uptake. Unfortunately, that information was not available to us."

7. Although linking higher anti-RBD levels to reported vaccination can strengthens internal validity, could the uptake be measured by card/register or other verification method as one might question the reliability of self-report sources

Thank you for that comment. We certainly agree but unfortunately, we did not have access to that information and that limitation is now mentioned in the Discussion section.

8. Yes, it discusses high exposure and vaccine uptake; however, it does not quantify herd immunity threshold or cold chain issues. Also, hesitancy not deeply analysed.

Serum was separated onsite using portable centrifuges and stored -20 °C under cold-chain conditions. Each sample was labelled with a unique study identifier and linked to participant data through encrypted REDCap databases. Stored and processed samples were transported from the site using a dry shipper (pre-charged to -20 °C) to the ACEGID laboratory at Redeemer's University for bead-based multiplex serology (Luminex MagPix). This is now included in the Methods section from lines 210-215.

Reviewer #2:

The study COVID-19 Sero-epidemiology and Vaccine Uptake in Two Southern Nigerian Clinics is certainly relevant and important. However, my main comment: Please ensure that the statistics reported in the results narrative correspond exactly to those in the tables. Use totals that exclude missing values (e.g., Table 1) and avoid referencing any statistics not presented in the tables. This consistency is critical for data accuracy.

Thank you very much for the detailed review and helpful feedback. The thoughtful review has helped improve the manuscript and streamline the results. All the comments have now been addressed, please see below in blue.

Suggestions for Authors

1. In the results, improve the paragraph flow, clarity, and consistency to separate the total number of participants and where they we recruited with the total in line 247 - 248.
   This has been adjusted generally throughout the text and detailed Supplemental Tables added to clarify.

2. Correct the phrasing in line 249, (n=430, 57.7%) is not about half. That's more than half or a clear majority.
   This has now been corrected to state: Over half of the participants were female (n=430, 57.7%), while 28.9% (n=215) were male, and 13.4% (n=100) did not report sex.

3. Also correct the n totals for both Female and Male as indicated in Table 1, line 149.
   The results and tables have now been streamlined, and Supplemental Tables added.

4. The n total for missing sex (unreported is not 100), correct the n and the percentage on line 250.
   The results and tables have now been streamlined, and Supplemental Tables added.

5. Please state the reported average Age as mean line 250; also, both mean and SD can be reflected in Table 1 as well for clarity.
   Average is now mean and Table 1 removed.

6. Instead of reporting this, "while the majority of participants were between 18 and 45 years of age (n=497, 66.7%)". For more clarity to this paper, you could re-categorize Age as Minors, Young, Middle-aged aged and Elderly per the WHO standardization. Also, correct the n total (497) line 252.
   We removed the sentence referring to the 18-45 age category. The most reliable WHO age category reference we could find (see below link) recommends using 11 age categories (separated by 5 years each), in which case a continuous age category makes more sense. We now included age both as continuous and categorical variable in the multivariable analysis and used 6 categories that align better with general health and life stages, see Supplemental Table 3.

   https://cdn.who.int/media/docs/default-source/documents/ddi/indicatorworkinggroup/glossary_of_terms_june-2024.pdf?sfvrsn=ac699779_1

7. Correct the statistics for Religion for all categories as indicated in Table 1, ie, Christianity, Islam, etc., line 255.
   The results and tables have now been streamlined.

8. Paraphrase the wording in line 259 for improved flow and clarity.
   This has been done.

9. Categorize the age variable and be consistent with the categories across the narrative, ie, line 412 has "young/middle-aged group (18–45 years)", which is not consistent with the earlier write-up. Follow guidance like in the earlier comment.
   This has been done, see above.

10. Pair p-values with effect size (if possible) instead of only saying "statistically significant ($p < 0.05$)"; you can include odds ratios (OR) or percentage point differences where applicable. (i.e., Line 265 - 266, you would say, "Participants from Owo were more likely to be vaccinated than those from Ikorodu (80.2% vs.52.6%, $\chi^2 = 62.1$, df = 1, $p < 0.001$)."
    This has been done.

11. Correct the result section, use "p = 0.038" instead of "p value = 0.038" for consistency with journal conventions, but not both interchangeably.
    We now use "p-value" throughout.

12. Keep consistent decimal formatting across line 289, 314.

This has been corrected (2 decimals throughout), except for the p-value of 0.0001 which needs 3 decimals to show significance/strength of result.

13. Some ORs are reported in the narrative but have no originality. Please add the Odds ratios in the Table and in narratives.
    This has been done, please see new Figures and Supplemental Tables.

14. Bold line 120, 142, 162, 172, 201, 246, 257, 324, 410, 417, 432, 443, 487, 503, 515 as a subheading.
    The sub header titles are now displayed in bold and italicized.

15. Please don't indent paragraphs. Kindly correct lines 86, 97, 108, 129, 135, 150, 205, 213, 285, 334, 459, 474. Consider triple spacing for new paragraphs.
    The indents have been removed throughout the manuscript.

16. Have the figures be displayed visibly in the write-up up than having links to figures.
    To comply with the journal submission guidelines, we uploaded the Figure files separately. We are glad to make that change if this format is preferred. PLOS GPH submission guidelines: (https://journals.plos.org/globalpublichealth/s/submission-guidelines).
