# Supplementary material for: COVID19 Sero-epidemiology and vaccine uptake in two Southern Nigerian clinics
Source: PLOS Glob Public Health. 2026 Mar 27;6(3):e0006126. doi: 10.1371/journal.pgph.0006126 (PMC13029786; doi:10.1371/journal.pgph.0006126)
Supplement: Attachment — Submitted filename: Review Comments_PGPH-D-25-02354_1.pdf [file pgph.0006126.s024.pdf]

Reviewer comments: (Geofrey Basalirwa)

- **Comment 1:** In the Author's list, write the name without diacritics in line 7 for consistency across.

- **Comment 2:** Check the decimal points used for the p-values. Maintain the same number of decimal points across the paper. Compare lines 45, 46, and 48,305,306, 307,313, 324, 325. Check for all p-values.

- **Comment 3:** The grammatical flow of the last sentence in lines 173 and 174 needs to be corrected. Adding "that" after ensured improves clarity and flow.

- **Comment 4:** For inclusion, be specific about the age or age range. Line 163 says, "Individuals across all ages," while in the results section, line 278 says, "Ages ranged from 0 to 84 years". Be consistent.

- **Comment 5:** The total for "No formal education" 8(1.1%) is not reported in the results section. Line 279 – 282. Making the Total number of education under-reported. This was combined in the supplemental table, but there was no mention of it. Correct this.

- **Comment 6:** Please only report the p-value in line 296.

- **Comment 7:** Bold line 347.

- **Comment 8:** Remove the "and" in the last sentence in line 389.

- **Comment 9:** Use "p = 0.001" instead of "p-value = 0.001" for consistency with journal conventions, but not both interchangeably in lines 404, 405, 407, and 408, 507,509, 513, also mindful of the decimal places (choose either 2 or 3), be consistent.

Comments and Suggestions for Authors

Dear Authors,

Thank you for considering and addressing the earlier comments. I have a few more minor suggestions:

1. In the Author's list, write the name without diacritics in line 7 for consistency across.

2. Check the decimal points used for the p-values. Maintain the same number of decimal points across the paper. Compare lines 45, 46, and 48,305,306, 307,313, 324, 325.

3. Improve the grammatical flow of the last sentence in lines 173 and 174. Adding "that" after ensured improves clarity and flow.

4. For inclusion, be specific about the age or age range. Line 163 says, “Individuals across all ages,” while in the results section, line 278 says, “Ages ranged from 0 to 84 years”. Be consistent.

5. The total for “No formal education” 8(1.1%) is not reported in the results section. Line 279 – 282. Making the Total number of education under-reported. This was combined in the supplemental table, but there was no mention of it. Correct this.

6. Please only report the p-value in line 296.

7. Bold line 347.

8. Remove the “and” in the last sentence in line 389.

9. Use “p = 0.001” instead of “p-value = 0.001” for consistency with journal conventions, but not both interchangeably in lines 404, 405, 407, and 408, 507,509, 513, also mindful of the decimal places (choose either 2 or 3), be consistent.

Date of this review
09 FEB 2026
