# Supplementary material for: COVID19 Sero-epidemiology and vaccine uptake in two Southern Nigerian clinics
Source: PLOS Glob Public Health. 2026 Mar 27;6(3):e0006126. doi: 10.1371/journal.pgph.0006126 (PMC13029786; doi:10.1371/journal.pgph.0006126)
Supplement: Attachment — Submitted filename: PLOS PGH Response_2 18 26.pdf [file pgph.0006126.s026.pdf]

We thank the reviewers and editorial team for the second round of feedback on our manuscript, "COVID19 Sero-epidemiology and Vaccine Uptake in Two Southern Nigerian Clinics" (PGPH-D-25-02354R1). The feedback has helped further improve and streamline the text and results.

In response, we have revised the manuscript and integrated the comments and suggestions sent by the reviewers. We did not make any changes to the figures or supplemental information. Please see below the integrated responses in blue. Note, the line numbers correspond to the manuscript in track changes.

We are grateful for the reviewers' insights, which have guided substantial improvements to both the scientific robustness and the practical significance of the paper.

Reviewer #1:

I believe that the authors have satisfactorily addressed the substantive scientific and methodological comments from the previous review round. The added multivariable analyses, clearer limitations, and strengthened interpretation make the manuscript suitable for publication. However, minor editorial and consistency issues remain:

1. Results narrative vs tables/figures: In the Results section, some percentages are described without explicitly stating that missing values are excluded, while the corresponding tables use reduced denominators (e.g., comparison between narrative descriptions of seroprevalence and Table 1 totals. Consider aligning text strictly with table denominators. Also, "Age was the strongest predictor…, while sex and education were…", with the corresponding adjusted odds ratios and confidence intervals could be explicitly linked to a table reference (e.g., "Table 3") in the narrative, which would strengthen transparency. Thank you for the detailed review. We went through the manuscript and aligned the results and tables, with focus on supplemental tables that list the missing data points. Similarly, we checked the results for Table 1 but could not find discrepancies. There is no Table 3 in the manuscript, so we are not sure what you mean. We checked Supplemental Table 3 and the results and Table align.

2. Terminology consistency: The assay threshold terminology (e.g., variants of the "3 SDTV" threshold notation) is not used consistently across the Methods and Results sections. Thank you for catching the typo. It is now 3STDV throughout.

3. Figure caption wording: Figure 3 caption contains a duplicated sentence and a phrase that appears inconsistent with the figure content (reference to "odds of vaccination" in a seropositivity figure). We checked the caption for Figure 3 and did not find a duplicate sentence or misalignment with the results.

4. Comparative numerical results: Please consider presenting results consistently from lowest to highest values when highlighting differences between methods, e.g., reporting the lower estimate first (3STDV: 10.5%) followed by the higher estimate (SeroNIST: 82.2%, 95% CI ±10.2) would make the contrast between methods easier to follow. Table 1 lists the seroprevalence results for each method. The results in the table are listed by antigens rather than by matching results to (i) outline the biological differences, (ii) underline that RBD is a subset of S (and N is separate/more distant), and

(iii) depict the RBD variants as they evolved over time (wildtype [WT] first, and end with Omicron). We hope that makes sense.

These are straightforward corrections that do not affect the validity of the findings. Assuming all underlying datasets and supporting information have been submitted as stated, and subject to these minor revisions, I support publication in PLOS Global Public Health.
Thank you very much for the detailed review and constructive feedback.

Reviewer #2:

1. In the Author's list, write the name without diacritics in line 7 for consistency across. The name Olayiwola is now written without diacritics.

2. Check the decimal points used for the p-values. Maintain the same number of decimal points across the paper. Compare lines 45, 46, and 48,305,306, 307,313, 324, 325. The p-values all have three decimal points, expect when the value was very small, then we used "< 0.0001" as is standard in epidemiological reporting to underling the strong effect of that outcome. We hope that makes sense.

3. Improve the grammatical flow of the last sentence in lines 173 and 174. Adding "that" after ensured improves clarity and flow. We went through the whole Methods section, covering those lines, and could not identify a missing "that". We are glad to adjust if you point us toward that sentence.

4. For inclusion, be specific about the age or age range. Line 163 says, "Individuals across all ages," while in the results section, line 278 says, "Ages ranged from 0 to 84 years". Be consistent. The inclusion criteria stated we were open to enroll participants across all ages, and we ended up enrolling participants with ages ranging from 0 to 84 years. I hope this shows that it is consistent and correct.

5. The total for "No formal education" 8(1.1%) is not reported in the results section. Line 279 – 282. Making the Total number of education under-reported. This was combined in the supplemental table, but there was no mention of it. Correct this. The following sentence is currently in the results (see line 288-289) and matches the supplemental Table 8: "A total of 54 individuals (7.2%) did not report education."

6. Please only report the p-value in line 296. Line 296 does not currently have relevant results.

7. Bold line 347. Thank you for catching inconsistencies in subtitle formatting. This has been fixed.

8. Remove the "and" in the last sentence in line 389. Thank you for catching that typo. It has been fixed.

9. Use "p = 0.001" instead of "p-value = 0.001" for consistency with journal conventions, but not both interchangeably in lines 404, 405, 407, and 408, 507,509, 513, also mindful of the decimal places (choose either 2 or 3), be consistent. We checked the full manuscript, and it states p-value throughout. The p-values all have three decimal points, expect when the value was very small, then

we used “< 0.0001” as is standard in epidemiological reporting to underling the strong effect of that outcome. We hope that makes sense.

Reviewer #3:

This manuscript presents a thorough and valuable investigation of SARS-CoV-2 seroprevalence, vaccine uptake, and methodological differences in serological threshold setting among febrile patients affiliated with peri-urban and urban clinics in Southern Nigeria. The comparison between peri-urban and urban patients seems to be a relatively novel consideration. Several vaccines that were in use during the relevant period were also accounted for in the study.
The study yields valuable insights into SARS-Cov-2 seroprevalence, vaccine uptake, and seroprevalence estimates in relation to several demographic factors. Some of these results are interesting and would be unexpected. In particular, the finding that basic socioeconomic factors were not significant determinants of vaccine uptake is of interest.
The proposed framework demonstrates potential applicability to other contexts as well.
The literature review is thorough and the methodology is robust.
Corrections suggested by the original reviewers have been carefully incorporated. These changes have significantly improved the manuscript.
Thank you very much for the detailed review and constructive feedback.

Please include in-text citations using brackets, not inside parentheses.
We are following the journal guidelines and applied the Vancouver citation style. However, we are glad to adjust as needed.

I have highlighted some words/phrases with comments for potential re-wording for clarity. Please hover over highlighted text (in light blue) to read the comments. Please carefully review that feedback and revise the sentences accordingly.
We went through the comments and integrated them. Thank you for catching all those small mistakes and typos.
