# Supplementary material for: COVID19 Sero-epidemiology and vaccine uptake in two Southern Nigerian clinics
Source: PLOS Glob Public Health. 2026 Mar 27;6(3):e0006126. doi: 10.1371/journal.pgph.0006126 (PMC13029786; doi:10.1371/journal.pgph.0006126)
Supplement: Attachment — Submitted filename: PLOS PGH Response_Dec 17 2025.docx [file pgph.0006126.s025.docx]

We thank the reviewers and editorial team for the feedback on our manuscript, “COVID19 Sero-epidemiology and Vaccine Uptake in Two Southern Nigerian Clinics” (PGPH-D-25-02354). The feedback has significantly improved the statistical approach, results description, formatting, and public health implication of the manuscript and applied analyses.

In response, we have substantially revised the manuscript. Please see below the integrated responses in blue. Note, the line numbers correspond to the manuscript in track changes. In summary, we improved the Methods section to provide more information on the serological method and its importance. We added extensive multivariable analyses to evaluate vaccine uptake and seropositivity in terms of adjusted odds ratios and included detailed outcomes in the Abstract, Methods, Results (including figures/forest plots), Discussion, and Supplemental Materials to give readers more details. We clarified the missing data and included it in the logistic regressions. Finally, we expanded the Discussion to address shortcomings, including the fact that our study population covered only symptomatic individuals.

We are grateful for the reviewers’ insights, which have guided substantial improvements to both the scientific robustness and the practical significance of the paper.

**Journal Requirements:**

1. Please include a complete copy of PLOS’ questionnaire on inclusivity in global research in your revised manuscript. The questionnaire has been filled out and included.
2. Please provide a detailed online Financial Disclosure statement. a. State the initials, alongside each funding source, of each author to receive each grant. For example: “This work was supported by the National Institutes of Health (####### to AM; ###### to CJ) and the National Science Foundation (###### to AM).” b. State what role the funders took in the study. The financial disclosure statement has been updated in the portal and in the manuscript.
3. Please ensure that the funders and grant numbers match between the Financial Disclosure field and the Funding Information tab in your submission form. Note that the funders must be provided in the same order in both places as well. This has been done.
4. We note that your Data Availability Statement is currently as follows: “The full database has been submitted along with the manuscript and figures.” Please confirm at this time whether or not your submission contains all raw data required to replicate the results of your study. We confirm that the full database has been submitted.
5. Please provide separate main figure files in .tif or .eps format only and ensure that all files are under our size limit of 10MB. For more information about how to convert your figure files please see our guidelines: <https://journals.plos.org/globalpublichealth/s/figures>. We strongly recommend that you use PLOS’s NAAS tool (https://ngplosjournals.pagemajik.ai/artanalysis) to test your figure files. The figures have been reformatted and checked with the PLOS’s NAAS tool.

**Editor Comments:**

Please address the comments brought forth by the reviewers with a focus on including a more comprehensive multivariable analysis for factors associated with seroprevalence in the total study population and in the unvaccinated study population. The potential confounders included in the model should be justified based on literature and 95% CIs should be included in tables and in the text when referencing results from the regression analyses. Clear tables should be included in the manuscript that identify the results from the regression model(s). Also, stronger justification for excluding missing data should be included vs imputation. Where the patterns of missingness examined?

Thank you very much for the helpful feedback. To determine whether sex, age, education, religion, city of residence, or vaccination status independently increased or decreased the odds of being positive for anti-N WT SARS-CoV-2 antibodies, we now included a multivariable analysis (i.e., logistic regression with binary seropositivity outcomes, see Supplemental Table 8, Supplemental Table 9, Supplemental Table 13, and Figure 1). These variables and the associated literature are cited in the Discussion. We focused on the N-based seroprevalence because all the COVID-19 vaccines distributed in Nigeria covered S/RBD but not N antigens. Thus, the presence of anti-N SARS-CoV-2 antibodies were predictive of natural SARS-CoV-2 infection and not vaccination in this setting. Further, the RBD- and S-based seroprevalences were very high (ranged from 82.2% to 100%) and preclude a meaningful analysis (i.e., not enough negative outcomes, see Table 1). The outcomes now include adjusted odds ratios (aORs), respective 95% confidence intervals, and p-values. Further, to determine which of the included variables independently increase or decrease the odds of having received a COVID-19 vaccine, we ran a similar multivariable analysis. Those results and tables are also included in detail, see Figure 3, and Supplemental Table 14. The missing data was now analyzed and included as separate categories in the regressions, except for age, because a single participant was missing age.

**Reviewer #1:**

I have now completed my review of the paper and incorporated my comments directly into the text for your consideration. The main recommendations focus on:

Thank you very much for the detailed review and helpful feedback. The multivariable analysis and all the other suggestions were very helpful and added interesting and more robust results. All the comments have now been addressed, please see below in blue.

1. Inclusion criteria considering that sampling is limited to febrile outpatients, and so generalizability is reduced.

Yes, unfortunately our study team only had access to febrile study participants which is not representative of the general population. The fact that our study population is febrile (i.e., symptomatic) is stated in the Introduction, Methods, Results, and discussed as limitation in the Discussion, see track changes and particularly lines 929-932.

2. The fact that vaccination data is self-reported. You may want to consider clarifying the risk of misclassification.

Thank you for that comment. We now included the following section in the Discussion on lines 890-895: “While we were able to enroll 745 study participants, increasing the limited sample size, including additional socioeconomic variables, and being able to access vaccination dates would have given a more nuanced picture of risk factors for SARS-CoV-2 exposure and COVID-19 vaccine uptake. Unfortunately, that information was not available to us. Further, the socio-demographic information and vaccine uptake were self-reported which may lead to recall bias or misrepresentations.”

3. On the statistics, there is limited multivariate analysis. I recommend stronger modelling for uptake predictors.

Thank you for the comment. The manuscript now includes extensive multivariable analyses,

adjusted odds ratios (aORs), respective 95% confidence intervals, and p-values in the main text,

Figures, and Supplemental Materials.

4. No explicit herd immunity threshold or practical cold-chain/coverage was discussed. If possible, it should be reviewed.

Serum was separated onsite using portable centrifuges and stored -20 °C under cold-chain conditions. Each sample was labelled with a unique study identifier and linked to participant data through encrypted REDCap databases. Stored and processed samples were transported from the site using a dry shipper (pre-charged to -20 °C) to the ACEGID laboratory at Redeemer’s University for bead-based multiplex serology (Luminex MagPix). This is now included in the Methods section from lines 210-215.

Comments:

1. Introduction - Could a clearer framing of why a sero-survey matters be incorporated? Is it possible to highlight the study’s novelty such as variant-specific immunity, standardized thresholding and vaccine uptake linkage?

Thank you for the comment. We have now edited the introduction to clarify, see track changes

throughout introduction.

1. Have some concerns with the inclusion criteria as it may not be fully representative of the general population and can introduce bias towards health-system users. Was there a reason for not using a not probability-based sampling.

Yes, unfortunately our study team only had access to febrile study participants which is not representative of the general population. The fact that our study population is febrile (i.e., symptomatic) is stated in the Introduction, Methods, Results, and discussed as limitation in the Discussion, see track changes and particularly lines 929-932.

2. There is clarity on the method described with antigen panels and controls. However, the performance metrics (sensitivity/specificity) has not been also clearly stated.

Thank you for that comment. We now include sensitivity and specificity in the seroprevalence

outcomes, see Table 1.

3. The assay workflow is well detailed, but was there a reason not to mention or not having a quality control procedure such as duplicate testing, external QC panels or inter-lab validation?

Thank you for the comment. In-depth validations, including across our teams in Nigeria and the US were done prior to the study. This included an in-person visit of Nigerian staff to the US for training purposes. Further, the serological platform was evaluated, validated, and used for SARS-CoV-2 seroprevalence studies prior to this, see references 23-25. This is now mentioned in the Methods.

4. Reported single/double doses and age focus, but no timing since vaccination, no booster details.

Thank you for that comment. Unfortunately, we did not have access to that information. We now included the following section in the Discussion on lines 890-895: “While we were able to enroll 745 study participants, increasing the limited sample size, including additional socioeconomic variables, and being able to access vaccination dates would have given a more nuanced picture of risk factors for SARS-CoV-2 exposure and COVID-19 vaccine uptake. Unfortunately, that information was not available to us. Further, the socio-demographic information and vaccine uptake were self-reported which may lead to recall bias or misrepresentations.”

5. To access appropriately the risk factors/effectiveness could a multivariable logistic regression for vaccine uptake or infection risk be used beyond basic comparisons?

Thank you for the comment. The manuscript now includes extensive multivariable analyses,

adjusted odds ratios (aORs), respective 95% confidence intervals, and p-values in the main text,

Figures, and Supplemental Materials.

6. Reported single/double doses and age focus, but no timing since vaccination, no booster details. Also, the vaccine uptake model used here could have been made stronger if other social determinants data such as income, occupation, mobility or health literacy were not collected.

Thank you for that comment. Unfortunately, we did not have access to that information. We now included the following section in the Discussion on lines 890-895: “While we were able to enroll 745 study participants, increasing the limited sample size, including additional socioeconomic variables, and being able to access vaccination dates would have given a more nuanced picture of risk factors for SARS-CoV-2 exposure and COVID-19 vaccine uptake. Unfortunately, that information was not available to us.”

7. Although linking higher anti-RBD levels to reported vaccination can strengthens internal validity, could the uptake be measured by card/register or other verification method as one might question the reliability of self-report sources

Thank you for that comment. We certainly agree but unfortunately, we did not have access to that information and that limitation is now mentioned in the Discussion section.

8. Yes, it discusses high exposure and vaccine uptake; however, it does not quantify herd immunity threshold or cold chain issues. Also, hesitancy not deeply analysed.

Serum was separated onsite using portable centrifuges and stored -20 °C under cold-chain conditions. Each sample was labelled with a unique study identifier and linked to participant data through encrypted REDCap databases. Stored and processed samples were transported from the site using a dry shipper (pre-charged to -20 °C) to the ACEGID laboratory at Redeemer’s University for bead-based multiplex serology (Luminex MagPix). This is now included in the Methods section from lines 210-215.

**Reviewer #2:**

The study COVID-19 Sero-epidemiology and Vaccine Uptake in Two Southern Nigerian Clinics is certainly relevant and important. However, my main comment: Please ensure that the statistics reported in the results narrative correspond exactly to those in the tables. Use totals that exclude missing values (e.g., Table 1) and avoid referencing any statistics not presented in the tables. This consistency is critical for data accuracy.

Thank you very much for the detailed review and helpful feedback. The thoughtful review has helped improve the manuscript and streamline the results. All the comments have now been addressed, please see below in blue.

Suggestions for Authors

1. In the results, improve the paragraph flow, clarity, and consistency to separate the total number of participants and where they we recruited with the total in line 247 - 248.

This has been adjusted generally throughout the text and detailed Supplemental Tables added to clarify.

2. Correct the phrasing in line 249, (n=430, 57.7%) is not about half. That’s more than half or a clear majority.

This has now been corrected to state: Over half of the participants were female (n=430, 57.7%), while 28.9% (n=215) were male, and 13.4% (n=100) did not report sex.

3. Also correct the n totals for both Female and Male as indicated in Table 1, line 149.

The results and tables have now been streamlined, and Supplemental Tables added.

4. The n total for missing sex (unreported is not 100), correct the n and the percentage on line 250.

The results and tables have now been streamlined, and Supplemental Tables added.

5. Please state the reported average Age as mean line 250; also, both mean and SD can be reflected in Table 1 as well for clarity.

Average is now mean and Table 1 removed.

6. Instead of reporting this, “while the majority of participants were between 18 and 45 years of age (n=497, 66.7%)”. For more clarity to this paper, you could re-categorize Age as Minors, Young, Middle-aged aged and Elderly per the WHO standardization. Also, correct the n total (497) line 252.

We removed the sentence referring to the 18-45 age category. The most reliable WHO age category reference we could find (see below link) recommends using 11 age categories (separated by 5 years each), in which case a continuous age category makes more sense. We now included age both as continuous and categorical variable in the multivariable analysis and used 6 categories that align better with general health and life stages, see Supplemental Table 3.

https://cdn.who.int/media/docs/default-source/documents/ddi/indicatorworkinggroup/glossary_of_terms_june-2024.pdf?sfvrsn=ac699779_1

7. Correct the statistics for Religion for all categories as indicated in Table 1, ie, Christianity, Islam, etc., line 255.

The results and tables have now been streamlined.

8. Paraphrase the wording in line 259 for improved flow and clarity.

This has been done.

9. Categorize the age variable and be consistent with the categories across the narrative, ie, line 412 has “young/middle-aged group (18–45 years)”, which is not consistent with the earlier write-up. Follow guidance like in the earlier comment.

This has been done, see above.

10. Pair p-values with effect size (if possible) instead of only saying “statistically significant (p < 0.05)”; you can include odds ratios (OR) or percentage point differences where applicable. (i.e., Line 265 - 266, you would say, “Participants from Owo were more likely to be vaccinated than those from Ikorodu (80.2% vs.52.6%, χ² = 62.1, df = 1, p < 0.001).”

This has been done.

11. Correct the result section, use “p = 0.038” instead of “p value = 0.038” for consistency with journal conventions, but not both interchangeably.

We now use “p-value” throughout.

12. Keep consistent decimal formatting across line 289, 314.

This has been corrected (2 decimals throughout), except for the p-value of 0.0001 which needs 3 decimals to show significance/strength of result.

13. Some ORs are reported in the narrative but have no originality. Please add the Odds ratios in the Table and in narratives.

This has been done, please see new Figures and Supplemental Tables.

14. Bold line 120, 142, 162, 172, 201, 246, 257, 324, 410, 417, 432, 443, 487, 503, 515 as a subheading.

The sub header titles are now displayed in bold and italicized.

15. Please don’t indent paragraphs. Kindly correct lines 86, 97, 108, 129, 135, 150, 205, 213, 285, 334, 459, 474. Consider triple spacing for new paragraphs.

The indents have been removed throughout the manuscript.

16. Have the figures be displayed visibly in the write-up up than having links to figures.

To comply with the journal submission guidelines, we uploaded the Figure files separately. We are glad to make that change if this format is preferred. PLOS GPH submission guidelines: (<https://journals.plos.org/globalpublichealth/s/submission-guidelines>).
